# Estimation methods based on ranked set sampling for the power logarithmic distribution

**DOI:** 10.1038/s41598-024-67693-4

**Published:** 2024-07-31

**Authors:** Najwan Alsadat, Amal S. Hassan, Mohammed Elgarhy, Arne Johannssen, Ahmed M. Gemeay

**Affiliations:** 1 Department of Quantitative Analysis, College of Business Administration, King Saud University, P.O. Box 71115, 11587 Riyadh, Saudi Arabia; 2 Faculty of Graduate Studies for Statistical Research, Cairo University, 5 Dr. Ahmed Zewail Street, Giza, 12613 Egypt; 3 Mathematics and Computer Science Department, Faculty of Science, Beni-Suef University, Beni-Suef, 62521 Egypt; 4 Department of Basic Sciences, Higher Institute of Administrative Sciences, Belbeis, Al Sharkia Egypt; 5 Faculty of Business Administration, University of Hamburg, 20146 Hamburg, Germany; 6 Department of Mathematics, Faculty of Science, Tanta University, Tanta, 31527 Egypt

**Keywords:** Power logarithmic distribution, Ranked set sampling, Minimum spacing Linex distance, Minimum spacing square log distance, Average squared absolute error, Engineering, Mathematics and computing

## Abstract

The sample strategy employed in statistical parameter estimation issues has a major impact on the accuracy of the parameter estimates. Ranked set sampling (RSS) is a highly helpful technique for gathering data when it is difficult or impossible to quantify the units in a population. A bounded power logarithmic distribution (PLD) has been proposed recently, and it may be used to describe many real-world bounded data sets. In the current work, the three parameters of the PLD are estimated using the RSS technique. A number of conventional estimators using maximum likelihood, minimum spacing absolute log-distance, minimum spacing square distance, Anderson-Darling, minimum spacing absolute distance, maximum product of spacings, least squares, Cramer-von-Mises, minimum spacing square log distance, and minimum spacing Linex distance are investigated. The different estimates via RSS are compared with their simple random sampling (SRS) counterparts. We found that the maximum product spacing estimate appears to be the best option based on our simulation results for the SRS and RSS data sets. Estimates generated from SRS data sets are less efficient than those derived from RSS data sets. The usefulness of the RSS estimators is also investigated by means of a real data example.

## Introduction

In many domains, data analysis has been made simpler, and the margin of error has decreased with the discovery of new probability distributions. New unit distributions are typically created by converting certain well-known continuous distributions, which are more adaptable than the originals, without the need to include extra parameters. A number of distributions has been developed for the purpose of modeling data sets in numerous field, such as finance, risk management, engineering, actuarial sciences, biology, and economics. These distributions include the unit-Birnbaum-Saunders distribution^1^, the unit Weibull distribution^2^, the unit Gompertz distribution^3^, the unit-inverse Gaussian distribution^4^, the unit Burr-XII distribution^5^, the unit-Chen distribution^6^, the unit half-logistic geometric distribution^7^, the unit exponentiated Frechet distribution^8^, the unit exponentiated Lomax distribution^9^, the unit inverse exponentiated Weibull distribution^10^, the unit power Burr X distribution^11^, among others.

The power logarithmic distribution (PLD), which combines logarithmic and power function distributions, was introduced by Abd El-Bar et al.^12^. They mentioned that, compared to the power function distribution, the PLD is more flexible. The probability density function (PDF) of the PLD, with shape parameter $a > 0$ and scale parameters $b > 0$ and $c > 0$, is given by:

1 $$\begin{aligned} f(x;\omega ) = \frac{{{{(a + 1)}^2}{x^a}(b - c\ln (x))}}{{b + c + ab}},\,\,\,\,0\,< x < 1,\, \end{aligned}$$

where $\omega \equiv (a,b,c)$ is the set of parameters. The cumulative distribution function (CDF) of the PLD is:

2 $$\begin{aligned} F(x) = \frac{{{x^{a + 1}}\left[ {c + (a + 1)(b - c\ln (x))} \right] }}{{b + c + ab}},\,\,\,\,\,0< x < 1. \end{aligned}$$

According to PDF (1), the distributions mentioned below are considered as submodels of the PLD:

- For $c = 0$, the PDF (1) provides the power function distribution with parameter *a*.
- For $a = 0$, the PDF (1) provides the logarithmic distribution with parameters *b* and *c*.
- For $a + 1 = \vartheta$ and $c = 1$, the PDF (1) provides the Log-Lindley distribution with parameters $\vartheta$ and *b*.
- For $a + 1 = \vartheta$, $b = 0$, and $c = 1$, the PDF (1) provides the transformed gamma distribution with parameter $\vartheta$.

The hazard rate function (HF) of the PLD is:

$$\begin{aligned} h(x;\omega ) = \frac{{{{(a + 1)}^2}(b - c\ln (x))}}{{x\left[ {(a + 1)c\ln (x) - (b(a + 1) + c)(1 - {x^{ - a - 1}})} \right] }}.\, \end{aligned}$$

**Figure 1:**
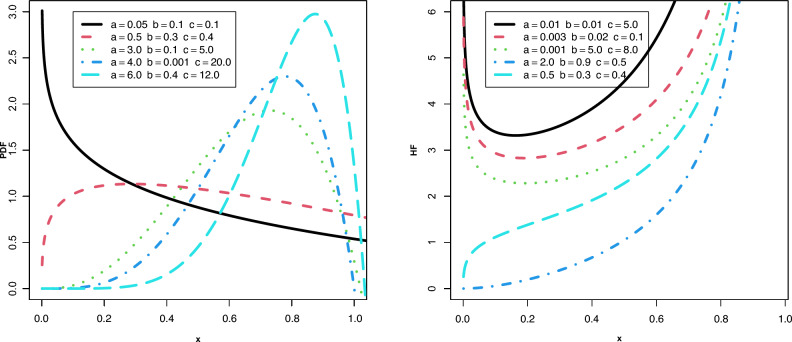
Plots of PDF and HF for the PLD.

The PDF and HF plots of the PLD are represented in Fig. 1. From Fig. 1, we can see that the PDF plot takes various shapes, such as growing, decreasing, constant, skewed to the right or left, and upside-down bathtub-shaped. The HF plots can be increasing, *U*-shaped, bathtub or *j*-shaped.

The study of economical sampling techniques is one of the major and fascinating areas of statistics. The field’s motivation stems from its exceptional ability to streamline the process of gathering data, particularly in situations when gathering relevant data is costly or time-consuming. In order to obtain accurate and cost-effective findings, researchers have developed a variety of sampling techniques over the past few decades. Ranked set sampling (RSS) is a useful technique for attaining observational economy in terms of the precision attained per sample unit. In the beginning, McIntyre^13^ presented the idea of RSS as a method for improving the sample mean’s accuracy as a population mean estimate. Ranking can be done without actually quantifying the observations by using expert opinion, visual examination, or any other method. Takahasi and Wakimoto^14^ provided the mathematical framework for RSS. Dell and Clutter^15^ demonstrated that, even in the presence of ranking errors, RSS outperforms simple random sampling (SRS). The RSS is extensively used in the fields of environmental monitoring^16^, entomology^17^, engineering applications^18^, forestry^19^, and information theory^20^.

The following is a description of the RSS design: Initially, $s^2$ randomly selected units are taken from the population and divided into *s* groups of *s* units each. Without using any measures, the *s* units in each set are ranked. The unit that ranks lowest among the first *s* units is selected for actual quantification. The unit that ranks second lowest among the second set of *s* units is measured. The procedure is carried out again until the largest unit is determined from the *s*th group of *s* units. Hence, $X_{\left( h \right) h} = {\text { }}\left( {{X_{\left( 1 \right) 1}}, {X_{\left( 2 \right) 2}},{\text { }}.{\text { }}.{\text { }}.{\text { }},{X_{\left( s \right) s}}} \right)$, $h = 1, \ldots , s$, represents the one-cycle RSS. The process can be repeated *l* times to produce a sample of size ${s^ \cdot } = sl$ if a larger number of samples is needed. The *l*- cycle RSS is represented as $X_{\left( h \right) hv} = {\text { }}\left( {{X_{\left( 1 \right) 11}}, {X_{\left( 2 \right) 22}},{\text { }}.{\text { }}.{\text { }}.{\text { }},{X_{\left( s \right) sl}}} \right)$, $h = 1, \ldots , s$ and $v = 1, \ldots , l$. In the present work, we write ${X_{hv}}$ instead of ${X_{(h)hv}}$. Wolfe^21^ mentioned that set sizes (*s*) larger than five would undoubtedly result in an excessive number of ranking errors and so could not likely considerably increase the efficacy of the RSS. Suppose that ${X_{hv}}$ represents the order statistics of the *h*th sample, with $h = 1, \ldots , s$ in the *v*th cycle. Assuming perfect ranking, the PDF of ${X_{hv}}$, is given by

3 $$\begin{aligned} f({x_{hv}}) = \frac{{s!}}{{(h - 1)!(s - h)!}}{\left[ {F({x_{hv}})} \right] ^{h - 1}}{\left[ {1 - F({x_{hv}})} \right] ^{s - h}}f({x_{hv}}),\,\,\,{x_{hv}} \in {\mathbb {R}}. \end{aligned}$$

The issue of RSS-based estimation for a variety of parametric models has been the subject of several studies recently. The location-scale family distributions’ parameter estimator was examined by Stokes^22^. Bhoj^23^ investigated the scale and location parameter estimates for the extreme value distribution. Abu-Dayyeh et al.^24^ used SRS, RSS and a modification of RSS to investigate various estimators for the location and scale parameters of the logistic distribution. Under RSS, median RSS (MRSS), and multistage MRSS in case of imperfect ranking, Lesitha and Yageen^25^ investigated the scale parameter of a log-logistic distribution. Inference of the log-logistic distribution parameters, based on moving extremes RSS, was discussed by He et al.^26^. Using RSS and SRS, Yousef and Al-Subh^27^ obtained the maximum likelihood estimators (MLEs), moment estimators, and regression estimators of the Gumbel distribution parameters. Regarding SRS, RSS, MRSS, and extreme RSS (ERSS), Qian et al.^28^ derived a number of estimators for the Pareto distribution parameters in the case where one parameter is known and both are unknown. The MLEs for the generalized Rayleigh distribution parameters were derived by Esemen and Gurler^29^, using SRS, RSS, MRSS and ERSS. In the framework of SRS, RSS, MRSS, and ERSS, Samuh et al.^30^ presented the MLEs of the parameters pertaining to the new Weibull-Pareto distribution. Yang et al.^31^ explored the Fisher information matrix of the log-extended exponential-geometric distribution parameters based on SRS, RSS, MRSS, and ERSS. Al-Omari et al.^32^ investigated the generalized quasi-Lindley distribution parameters using the following estimators: MLEs, maximum product of spacings (MXPS) estimators, weighted least squares estimators, least squares estimators (LSEs), Cramer-von-Mises (CRM) estimators, and Anderson-Darling (AD) estimators based on RSS. Further, Al-Omari et al.^33^ considered similar procedures discussed as in Al-Omari et al.^32^ to examine estimators of the x-gamma distribution. Under stratified RSS, Bhushan and Kumar^34^ examined the effectiveness of combined and separate log type class population mean estimators. The suggested estimators’ mean square error and bias expressions were determined. The efficiency criteria were provided and a theoretical comparison between the proposed and current estimators was conducted. For more recent studies, see^35–42^.

The statistical literature proposes different estimation techniques since parameter estimation is important in real-world applications. Parameter estimation frequently involves the use of conventional estimation techniques like the LSE and MLE approaches. Both of them have advantages and disadvantages, but the most often used estimation technique is the ML method. The parameters of the PLD may be estimated using eight other methods of estimation in addition to the widely used MLE and LSE. These eight methods are AD, minimum spacing absolute distance (SPAD), MXPS, minimum spacing absolute log distance (SPALoD), minimum spacing square distance (MSSD), CRM, minimum spacing square log distance (MSSLD), and minimum spacing Linex distance (MSLND). It is difficult to compare the theoretical performance of different techniques, hence, extensive simulation studies are carried out under various sample sizes and parameter values to assess the performance of different estimators. Using a simulation scheme, the various PLD estimators based on the RSS design are then contrasted with those offered by the SRS approach. In this regard, six evaluation criteria are employed to assess the effectiveness of the estimating techniques. As far as the authors are aware, no attempt has been made to compare all of these estimators under RSS for the PLD. This fact served as the novelty and motivation for this study as we compare all of these estimators under RSS for the PLD.

The following sections provide a rough outline of the article. The various estimation methods for the PLD under RSS are provided in “Estimation methods based on RSS”. Several PLD estimators under SRS are given in “Estimation methods based on SRS”. The Monte Carlo simulation analysis that compares the effectiveness of the RSS-based estimators is examined in “Numerical simulation”. In “Real data analysis”, data analysis on milk production is conducted to demonstrate the practical applicability of the recommended estimate techniques. Some closing thoughts are included in “Concluding remarks”.

## Estimation methods based on RSS

This section discusses ten different estimators for the PLD based on RSS. The suggested estimators are the MLE, AD estimator (ADE), CRM estimator (CRME), MXPS estimator (MXPSE), LSE, SPAD estimator (SPADE), SPALoD estimator (SPALoDE), MSSD estimator (MSSDE), MSSLD estimator (MSSLDE), and MSLND estimator (MSLNDE).

### Maximum likelihood method

In the following, the MLEs ${{\hat{a}}^{{\text {ML}}}}$ of *a*, ${{\hat{b}}^{{\text {ML}}}}$ of *b*, and ${{\hat{c}}^{{\text {ML}}}}$ of *c* for the PLD are obtained based on RSS. To get these estimators let ${X_{hv}} = \left\{ {{X_{hv}},\,\,h = 1,\ldots ,s,v = 1,\ldots ,l} \right\}$ be an RSS of size ${s^ \cdot } = sl$ with PDF (1) and CDF (2), where *l* is cycles count and *s* is the set size. The likelihood function (LF) of the PLD is obtained by inserting Eqs. (1) and (2) into Eq. (3) as follows:

$$\begin{aligned} L(\omega ) \propto \prod \limits _{v = 1}^l {\prod \limits _{h = 1}^s {{{\left[ {\frac{{x_{vh}^{a + 1}\left[ {c + \Lambda ({x_{vh}},\omega )} \right] }}{{b + c + ab}}} \right] }^{h - 1}}} } {\left[ {1 - \frac{{x_{vh}^{a + 1}\left[ {c + \Lambda ({x_{vh}},\omega )} \right] }}{{b + c + ab}}} \right] ^{s - h}}\frac{{(a + 1)^2x_{vh}^a\Lambda ({x_{vh}},\omega )}}{{b + c + ab}}, \end{aligned}$$

where $\Lambda ({x_{vh}},\omega ) = (a + 1)(b - c\ln ({x_{vh}})).$ The log-LF of the PLD, denoted by ${L^{RSS}},$ is as follows:

4 $$\begin{aligned} \begin{aligned} {L^{RSS}} \propto \sum \limits _{v = 1}^l {\sum \limits _{h = 1}^s {\left\{ {(h - 1)(a + 1)\log {x_{vh}} + (h - 1)\log \left[ {c + \Lambda ({x_{vh}},\omega )} \right] } \right\} } } - {s^ \cdot }h\log (b + c + ab) + 2{s^ \cdot }\log (a + 1) \\ \,\,\,\,\,\,\,\,\,\,\,\,\,\,\, + \sum \limits _{v = 1}^l {\sum \limits _{h = 1}^s {\log } } \Lambda ({x_{vh}},\omega ) + a\sum \limits _{v = 1}^l {\sum \limits _{h = 1}^s {\log } } ({x_{vh}}) + \sum \limits _{v = 1}^l {\sum \limits _{h = 1}^s {(s - h)\log \left[ {1 - \frac{{x_{vh}^{a + 1}\left[ {c + \Lambda ({x_{vh}},\omega )} \right] }}{{b + c + ab}}} \right] } }. \\ \end{aligned} \end{aligned}$$

The MLEs ${{\hat{a}}^{{\text {ML}}}},{{\hat{b}}^{{\text {ML}}}},{{\hat{c}}^{{\text {ML}}}}$ of *a*, *b*, *c* are obtained by maximizing (4) with respect to *a*, *b*, and *c*, as follows:

5 $$\begin{aligned}{} & {} \begin{aligned}{}&\frac{{\partial {L^{RSS}}}}{{\partial a}} = \sum \limits _{v = 1}^l {\sum \limits _{h = 1}^s {(h - 1)\left[ {\log ({x_{vh}}) + \frac{{{{\Lambda '}_a}({x_{vh}},\omega )}}{{\left[ {c + \Lambda ({x_{vh}},\omega )} \right] }}} \right] } } - \frac{{{s^ \cdot }hb}}{b+c+ab} + \frac{{{2s^ \cdot }}}{{a + 1}}\, \\&\quad + \sum \limits _{v = 1}^l {\sum \limits _{h = 1}^s {\frac{{{{\Lambda '}_a}({x_{vh}},\omega )}}{{\Lambda ({x_{vh}},\omega )}}} } + \sum \limits _{v = 1}^l {\sum \limits _{h = 1}^s {\log } } ({x_{vh}}) \\&\quad - {\sum \limits _{v = 1}^l {\sum \limits _{h = 1}^s {(s - h)\left[ {1 - \frac{{x_{vh}^{a + 1}\left[ {c + \Lambda ({x_{vh}},\omega )} \right] }}{{b + c + ab}}} \right] } } ^{ - 1}}\left[ {\frac{{x_{vh}^{a + 1}{{\Lambda '}_a}({x_{vh}},\omega ) + \left[ {c + \Lambda ({x_{vh}},\omega )} \right] x_{vh}^{a + 1}\ln ({x_{vh}})}}{{b + c + ab}}} \right] \\&\quad + {\sum \limits _{v = 1}^l {\sum \limits _{h = 1}^s {(s - h)\left[ {1 - \frac{{x_{vh}^{a + 1}\left[ {c + \Lambda ({x_{vh}},\omega )} \right] }}{{b + c + ab}}} \right] } } ^{ - 1}}\left[ {\frac{{bx_{vh}^{a + 1}\left[ {c + \Lambda ({x_{vh}},\omega )} \right] }}{{{{(b + c + ab)}^2}}}} \right] , \\ \end{aligned} \end{aligned}$$

6 $$\begin{aligned}{} & {} \begin{aligned}{}&\frac{{\partial {L^{RSS}}}}{{\partial b}} = \sum \limits _{v = 1}^l {\sum \limits _{h = 1}^s {\frac{{(h - 1)(a + 1)}}{{c + \Lambda ({x_{vh}},\omega )}}} } - \,\frac{{{s^ \cdot }h{(1+a)}}}{{b + c+ab}} + \sum \limits _{v = 1}^l {\sum \limits _{h = 1}^s {\frac{{a + 1}}{{\Lambda ({x_{vh}},\omega )}}\,} } \\&\quad - {\sum \limits _{v = 1}^l {\sum \limits _{h = 1}^s {(s - h)\left[ {1 - \frac{{x_{vh}^{a + 1}\left[ {c + \Lambda ({x_{vh}},\omega )} \right] }}{{b + c + ab}}} \right] } } ^{ - 1}}\left[ {\frac{{(a + 1)x_{vh}^{a + 1}}}{{(b + c + ab)}} - \frac{{\left[ {c + \Lambda ({x_{vh}},\omega )} \right] x_{vh}^{a + 1}(1 + a)}}{{{{(b + c + ab)}^2}}}} \right] , \\ \end{aligned} \end{aligned}$$

and

7 $$\begin{aligned} \begin{aligned} \frac{{\partial {L^{RSS}}}}{{\partial c}} = - \frac{{{s^ \cdot }h}}{b+c+ab} - \,\sum \limits _{v = 1}^l {\sum \limits _{h = 1}^s {\frac{{(a + 1)\ln ({x_{vh}})}}{{\Lambda ({x_{vh}},\omega )}}} } \,\, + \sum \limits _{v = 1}^l {\sum \limits _{h = 1}^s {\frac{{(h - 1)(1 - (a + 1)\ln ({x_{vh}}))}}{{c + \Lambda ({x_{vh}},\omega )}}} } + \\ \,\,\,\,\,\,\,\,\,\,\,\,\, - {\sum \limits _{v = 1}^l {\sum \limits _{h = 1}^s {(s - h)\left[ {1 - \frac{{x_{vh}^{a + 1}\left[ {c + \Lambda ({x_{vh}},\omega )} \right] }}{{b + c + ab}}} \right] } } ^{ - 1}}\left[ {\frac{{x_{vh}^{a + 1}\left[ {1 - (a + 1)\ln ({x_{vh}})} \right] }}{{(b + c + ab)}} - \frac{{x_{vh}^{a + 1}\left[ {c + \Lambda ({x_{vh}},\omega )} \right] }}{{{{(b + c + ab)}^2}}}} \right] , \\ \end{aligned} \end{aligned}$$

where $${\Lambda '_a}({x_{vh}},\omega ) = \frac{{\partial {\Lambda _a}({x_{vh}},\omega )}}{{\partial a}} = (b - c\ln ({x_{vh}})).$$

We can get the MLEs ${{\hat{a}}^{{\text {ML}}}},{{\hat{b}}^{{\text {ML}}}},{{\hat{c}}^{{\text {ML}}}}$ of *a*, *b*, *c* by setting Eqs. (5)–(7) equal to zero and solving them simultaneously. Note that nonlinear optimization techniques such as the quasi-Newton algorithm are often more effective for solving these equations.

### Anderson–Darling method

The class of minimal distance approaches includes the AD method. In this subsection, the ADEs of *a*, *b*, *c*, say ${{\hat{a}}^{{\text {AD}}}}$, ${{\hat{b}}^{{\text {AD}}}}$, ${{\hat{c}}^{{\text {AD}}}}$ of the PLD are obtained using RSS.

Suppose that ${X_{(1:{s^ \cdot })}},{X_{(2:{s^ \cdot })}},\ldots ,{X_{({s^ \cdot }:{s^ \cdot })}}$ are ordered RSS items taken from the PLD with sample size ${s^ \cdot } = sl,$ where *s* is set size and *l* is the cycle number. The ADEs ${{\hat{a}}^{{\text {AD}}}},{{\hat{b}}^{{\text {AD}}}},{{\hat{c}}^{{\text {AD}}}}$ of *a*, *b*, and *c* are derived by minimizing the function

8 $$\begin{aligned} {\text {A(}}\omega {\text {)}} &= - {s^ \cdot } - \frac{1}{{{s^ \cdot }}}\sum \limits _{i = 1}^{{s^ \cdot }} {(2i - 1)\left\{ {\log F\left( {{x_{(i:{s^ \cdot })}}\left| \omega \right. } \right) + \log {\bar{F}}\left( {{x_{({s^ \cdot } - i + 1:{s^ \cdot })}}\left| \omega \right. } \right) } \right\} } \, \\ & = - {s^ \cdot } - \frac{1}{{{s^ \cdot }}}\sum \limits _{i = 1}^{{s^ \cdot }} {(2i - 1)\left\{ {\log \left[ {\frac{{x_{(i:{s^ \cdot })}^{a + 1}\left[ {c + \Lambda ({x_{(i:{s^ \cdot })}},\omega )} \right] }}{{b + c + ab}}} \right] + \log \left[ {1 - \frac{{x_{(i:{s^ \cdot })}^{a + 1}\left[ {c + \Lambda ({x_{({s^ \cdot } - i + 1:{s^ \cdot })}},\omega )} \right] }}{{b + c + ab}}} \right] } \right\} }, \\ \end{aligned}$$

where ${\bar{F}}\left( {.\left| \omega \right. } \right)$ is the survival function. Alternatively, the ADEs ${{\hat{a}}^{{\text {AD}}}},{{\hat{b}}^{{\text {AD}}}}$, and ${{\hat{c}}^{{\text {AD}}}}$ of the PLD can be obtained by solving the subsequent non-linear equations in place of Eq. (8):

$$\begin{aligned}{} & {} \frac{{\partial {\text {A(}}\omega {\text {)}}}}{{\partial a}} = \sum \limits _{i = 1}^{{s^ \cdot }} {(2i - 1)\left\{ {\frac{{\,{\zeta _1}\left( {{x_{(i:{s^ \cdot })}}\left| \omega \right. } \right) }}{{F\left( {{x_{(i:{s^ \cdot })}}\left| \omega \right. } \right) }} - \frac{{{\zeta _1}\left( {{x_{({s^ \cdot } - i + 1:{s^ \cdot })}}\left| \omega \right. } \right) }}{{{\bar{F}}\left( {{x_{({s^ \cdot } - i + 1:{s^ \cdot })}}\left| \omega \right. } \right) }}} \right\} = 0,} \\{} & {} \quad \frac{{\partial {\text {A(}}\omega {\text {)}}}}{{\partial b}} = \sum \limits _{i = 1}^{{s^ \cdot }} {(2i - 1)\left\{ {\frac{{\,{\zeta _2}\left( {{x_{(i:{s^ \cdot })}}\left| \omega \right. } \right) }}{{F\left( {{x_{(i:{s^ \cdot })}}\left| \omega \right. } \right) }} - \frac{{{\zeta _2}\left( {{x_{({s^ \cdot } - i + 1:{s^ \cdot })}}\left| \omega \right. } \right) }}{{{\bar{F}}\left( {{x_{({s^ \cdot } - i + 1:{s^ \cdot })}}\left| \omega \right. } \right) }}} \right\} = 0,} \end{aligned}$$

and

$$\begin{aligned} \frac{{\partial {\text {A(}}\omega {\text {)}}}}{{\partial c}} = \sum \limits _{i = 1}^{{s^ \cdot }} {(2i - 1)\left\{ {\frac{{\,{\zeta _3}\left( {{x_{(i:{s^ \cdot })}}\left| \omega \right. } \right) }}{{F\left( {{x_{(i:{s^ \cdot })}}\left| \omega \right. } \right) }} - \frac{{{\zeta _3}\left( {{x_{({s^ \cdot } - i + 1:{s^ \cdot })}}\left| \omega \right. } \right) }}{{{\bar{F}}\left( {{x_{({s^ \cdot } - i + 1:{s^ \cdot })}}\left| \omega \right. } \right) }}} \right\} = 0,} \end{aligned}$$

where

9 $$\begin{aligned}{} & {} \begin{aligned}{}&{\zeta _1}\left( {{x_{(i:{s^ \cdot })}}\left| \omega \right. } \right) = \frac{\partial }{{\partial a}}\left[ {F\left( {{x_{(i:{s^ \cdot })}}\left| \omega \right. } \right) } \right] \\&\quad = \frac{{ - x_{(i:{s^ \cdot })}^{a + 1}c\ln ({x_{(i:{s^ \cdot })}}) + \left[ b+{\left[ {c + \Lambda ({x_{(i:{s^ \cdot })}},\omega )} \right] }{\ln x_{(i:{s^ \cdot })}} \right] x_{(i:{s^ \cdot })}^{a + 1}}}{{b + c + ab}} - \frac{ {x_{(i:{s^ \cdot })}^{a + 1}} {b\left[ {c + \Lambda ({x_{(i:{s^ \cdot })}},\omega )} \right] }}{{{{(b + c + ab)}^2}}}, \\ \end{aligned} \end{aligned}$$

10 $$\begin{aligned}{} & {} {\zeta _2}\left( {{x_{(i:{s^ \cdot })}}\left| \omega \right. } \right) = \frac{\partial }{{\partial b}}\left[ {F\left( {{x_{(i:{s^ \cdot })}}\left| \omega \right. } \right) } \right] = \frac{{x_{(i:{s^ \cdot })}^{a + 1}(a + 1)}}{{b + c + ab}} - \frac{{(1 + a)x_{(i:{s^ \cdot })}^{a + 1}\left[ {c + \Lambda ({x_{(i:{s^ \cdot })}},\omega )} \right] }}{{{{(b + c + ab)}^2}}}, \end{aligned}$$

and

11 $$\begin{aligned} {\zeta _3}\left( {{x_{(i:{s^ \cdot })}}\left| \omega \right. } \right) = \frac{\partial }{{\partial c}}\left[ {F\left( {{x_{(i:{s^ \cdot })}}\left| \omega \right. } \right) } \right] = \frac{{x_{(i:{s^ \cdot })}^{a + 1}\left[ {1 - (a + 1)\ln ({x_{(i:{s^ \cdot })}})} \right] }}{{b + c + ab}} - \frac{{x_{(i:{s^ \cdot })}^{a + 1}\left[ {c + \Lambda ({x_{(i:{s^ \cdot })}},\omega )} \right] }}{{{{(b + c + ab)}^2}}}. \end{aligned}$$

Also, ${\zeta _1}\left( {{x_{({s^ \cdot } - i + 1:{s^ \cdot })}}\left| \omega \right. } \right) ,$ ${\zeta _2}\left( {{x_{({s^ \cdot } - i + 1:{s^ \cdot })}}\left| \omega \right. } \right) ,$ and ${\zeta _3}\left( {{x_{({s^ \cdot } - i + 1:{s^ \cdot })}}\left| \omega \right. } \right)$ have the same expressions as (9), (10) and (11) by replacing the ordered sample ${x_{(i:{s^ \cdot })}}$ by the ordered sample ${x_{({s^ \cdot } - i + 1:{s^ \cdot })}}$.

### Cramer–von-Mises method

The CRM method is a member of the minimal distance method class. This subsection provides the CRME of the parameter *a*, denoted by ${{\hat{a}}^{{\text {CR}}}}$, the CRME of parameter *b*, denoted by ${{\hat{b}}^{{\text {CR}}}}$, and the CRME of parameter *c*, denoted by ${{\hat{c}}^{{\text {CR}}}}$, of the PLD using RSS.

Let ${X_{(1:{s^ \cdot })}},{X_{(2:{s^ \cdot })}},\ldots ,{X_{({s^ \cdot }:{s^ \cdot })}}$ are ordered RSS items taken from the PLD with sample size ${s^ \cdot } = sl,$ where *s* is set size and *l* is the cycle number. The CRMEs ${{\hat{a}}^{{\text {CR}}}},{{\hat{b}}^{{\text {CR}}}},{{\hat{c}}^{{\text {CR}}}}$ of *a*, *b*, and *c* are derived by minimizing the function

12 $$\begin{aligned} C(\omega ) = \frac{1}{{12{s^ \cdot }}} + \sum \limits _{i = 1}^{{s^ \cdot }} {{{\left\{ {F\left( {{x_{(i:{s^ \cdot })}}\left| \omega \right. } \right) - \frac{{2i - 1}}{{2{s^ \cdot }}}} \right\} }^2}} . \end{aligned}$$

Rather than using Eq. (12), these estimators can be obtained by solving the non-linear equations

$$\begin{aligned}{} & {} \frac{{\partial C(\omega )}}{{\partial a}} = \sum \limits _{i = 1}^{{s^ \cdot }} {\left\{ {F\left( {{x_{(i:{s^ \cdot })}}\left| \omega \right. } \right) - \frac{{2i - 1}}{{2{s^ \cdot }}}} \right\} {\zeta _1}\left( {{x_{(i:{s^ \cdot })}}\left| \omega \right. } \right) = 0,} \\{} & {} \quad \frac{{\partial C(\omega )}}{{\partial b}} = \sum \limits _{i = 1}^{{s^ \cdot }} {\left\{ {F\left( {{x_{(i:{s^ \cdot })}}\left| \omega \right. } \right) - \frac{{2i - 1}}{{2{s^ \cdot }}}} \right\} {\zeta _2}\left( {{x_{(i:{s^ \cdot })}}\left| \omega \right. } \right) = 0,} \end{aligned}$$

and

$$\begin{aligned} \frac{{\partial C(\omega )}}{{\partial c}} = \sum \limits _{i = 1}^{{s^ \cdot }} {\left\{ {F\left( {{x_{(i:{s^ \cdot })}}\left| \omega \right. } \right) - \frac{{2i - 1}}{{2{s^ \cdot }}}} \right\} {\zeta _3}\left( {{x_{(i:{s^ \cdot })}}\left| \omega \right. } \right) = 0,} \end{aligned}$$

where ${\zeta _1}\left( {{x_{(i:{s^ \cdot })}}\left| \omega \right. } \right) ,$ ${\zeta _2}\left( {{x_{(i:{s^ \cdot })}}\left| \omega \right. } \right) ,$ and ${\zeta _3}\left( {{x_{(i:{s^ \cdot })}}\left| \omega \right. } \right)$ are given in Eqs. (9), (10), and (11).

### Maximum product of spacings method

This subsection provides the MXPSE of the parameter *a*, denoted by ${{\hat{a}}^{{\text {MP}}}}$, the MXPSE of parameter *b*, denoted by ${{\hat{b}}^{{\text {MP}}}}$, and the MXPSE of parameter *c*, denoted by ${{\hat{c}}^{{\text {MP}}}}$, of the PLD using RSS.

Let ${X_{(1:{s^ \cdot })}},{X_{(2:{s^ \cdot })}},\ldots ,{X_{({s^ \cdot }:{s^ \cdot })}}$ are ordered RSS items from the PLD with sample size ${s^ \cdot } = sl,$ where *s* is set size and *l* is the cycle number. The uniform spacings are defined as the differences

$$\begin{aligned} {D_i}(\omega ) = F\left( {{x_{(i:{s^ \cdot })}}\left| \omega \right. } \right) - F\left( {{x_{(i - 1:{s^ \cdot })}}\left| \omega \right. } \right) ,\,\,\,\,\,i = 1,2,\ldots ,{s^ \cdot } + 1, \end{aligned}$$

where $F\left( {{x_{(0:{s^ \cdot })}}\left| \omega \right. } \right) = 0,\,\,\,\,F\left( {{x_{({s^ \cdot } + 1:{s^ \cdot })}}\left| \omega \right. } \right) = 1,$ such that $\sum \limits _{i = 1}^{{s^ \cdot } + 1} {{D_i}(\omega )} = 1.$

The MXPSEs ${{\hat{a}}^{{\text {MP}}}},{{\hat{b}}^{{\text {MP}}}},{{\hat{c}}^{{\text {MP}}}}$ of *a*, *b*, and *c* are found by maximizing the geometric mean of the spacing, which is obtained by maximizing the following function

$$\begin{aligned} {\text {M}} = \frac{1}{{{s^ \cdot } + 1}}\sum \limits _{i = 1}^{{s^ \cdot } + 1} {\ln \left[ {{D_i}(\omega )} \right] }. \end{aligned}$$

The MXPSEs ${{\hat{a}}^{{\text {MP}}}},{{\hat{b}}^{{\text {MP}}}},{{\hat{c}}^{{\text {MP}}}}$ are provided by solving numerically the equations

$$\begin{aligned}{} & {} \frac{{\partial {\text {M}}}}{{\partial a}} = \frac{1}{{1 + {s^ \cdot }}}\sum \limits _{i = 1}^{{s^ \cdot } + 1} {\frac{1}{{{D_i}(\omega )}}} \left[ {{\zeta _1}\left( {{x_{(i:{s^ \cdot })}}\left| \omega \right. } \right) - {\zeta _1}\left( {{x_{(i - 1:{s^ \cdot })}}\left| \omega \right. } \right) } \right] = 0, \\{} & {} \quad \frac{{\partial {\text {M}}}}{{\partial b}} = \frac{1}{{1 + {s^ \cdot }}}\sum \limits _{i = 1}^{{s^ \cdot } + 1} {\frac{1}{{{D_i}(\omega )}}} \left[ {{\zeta _2}\left( {{x_{(i:{s^ \cdot })}}\left| \omega \right. } \right) - {\zeta _2}\left( {{x_{(i - 1:{s^ \cdot })}}\left| \omega \right. } \right) } \right] = 0, \end{aligned}$$

and

$$\begin{aligned} \frac{{\partial {\text {M}}}}{{\partial c}} = \frac{1}{{1 + {s^ \cdot }}}\sum \limits _{i = 1}^{{s^ \cdot } + 1} {\frac{1}{{{D_i}(\omega )}}} \left[ {{\zeta _3}\left( {{x_{(i:{s^ \cdot })}}\left| \omega \right. } \right) - {\zeta _3}\left( {{x_{(i - 1:{s^ \cdot })}}\left| \omega \right. } \right) } \right] = 0, \end{aligned}$$

where ${\zeta _1}\left( {.\left| \omega \right. } \right) ,$ ${\zeta _2}\left( {.\left| \omega \right. } \right) ,$ and ${\zeta _3}\left( {.\left| \omega \right. } \right)$ are given in Eqs. (9), (10), and (11).

### Least squares method

Here, the LSE of the parameter *a*, denoted by ${{\hat{a}}^{{\text {LS}}}}$, the LSE of parameter *b*, denoted by ${{\hat{b}}^{{\text {LS}}}}$, and the LSE of parameter *c*, denoted by ${{\hat{c}}^{{\text {LS}}}}$, of the PLD are covered using RSS.

Suppose that ${X_{(1:{s^ \cdot })}},{X_{(2:{s^ \cdot })}},\ldots ,{X_{({s^ \cdot }:{s^ \cdot })}}$ are ordered RSS items from the PLD with sample size ${s^ \cdot } = sl,$ where *s* is set size and *l* is the cycle number. The LSEs ${{\hat{a}}^{{\text {LS}}}},{{\hat{b}}^{{\text {LS}}}}$ and ${{\hat{c}}^{{\text {LS}}}}$ , are obtained after minimizing the function

13 $$\begin{aligned} {\text {L}} = \sum \limits _{i = 1}^{{s^ \cdot }} {{{\left[ {F\left( {{x_{(i:{s^ \cdot })}}\left| \omega \right. } \right) - \frac{i}{{{s^ \cdot } + 1}}} \right] }^2}}, \end{aligned}$$

with respect to *a*, *b*, and *c*. The LSEs ${{\hat{a}}^{{\text {LS}}}},{{\hat{b}}^{{\text {LS}}}}$, and ${{\hat{c}}^{{\text {LS}}}}$ can be produced by solving the following non-linear equations

$$\begin{aligned}{} & {} \frac{{\partial {\text {L}}}}{{\partial a}} = \sum \limits _{i = 1}^{{s^ \cdot }} {\left[ {F\left( {{x_{(i:{s^ \cdot })}}\left| \omega \right. } \right) - \frac{i}{{{s^ \cdot } + 1}}} \right] } \,\,\,{\zeta _1}\left( {{x_{(i:{s^ \cdot })}}\left| \omega \right. } \right) = 0, \\{} & {} \quad \frac{{\partial {\text {L}}}}{{\partial b}} = \sum \limits _{i = 1}^{{s^ \cdot }} {\left[ {F\left( {{x_{(i:{s^ \cdot })}}\left| \omega \right. } \right) - \frac{i}{{{s^ \cdot } + 1}}} \right] } \,\,\,{\zeta _2}\left( {{x_{(i:{s^ \cdot })}}\left| \omega \right. } \right) = 0, \end{aligned}$$

and

$$\begin{aligned} \frac{{\partial {\text {L}}}}{{\partial c}} = \sum \limits _{i = 1}^{{s^ \cdot }} {\left[ {F\left( {{x_{(i:{s^ \cdot })}}\left| \omega \right. } \right) - \frac{i}{{{s^ \cdot } + 1}}} \right] } \,\,\,{\zeta _3}\left( {{x_{(i:{s^ \cdot })}}\left| \omega \right. } \right) = 0, \end{aligned}$$

where ${\zeta _1}\left( {.\left| \omega \right. } \right) ,$ ${\zeta _2}\left( {.\left| \omega \right. } \right) ,$ and ${\zeta _3}\left( {.\left| \omega \right. } \right)$ are given in (9), (10), and (11).

### Minimum spacing absolute distance

In the following, we obtain the SPADE of the parameter *a*, denoted by ${{\hat{a}}^{{\text {SPA}}}}$, the SPADE of parameter *b*, denoted by ${{\hat{b}}^{{\text {SPA}}}}$, and the SPADE of parameter *c*, denoted by ${{\hat{c}}^{{\text {SPA}}}}$, of the PLD using RSS.

Suppose that ${X_{(1:{s^ \cdot })}},{X_{(2:{s^ \cdot })}},\ldots ,{X_{({s^ \cdot }:{s^ \cdot })}}$ are ordered RSS items from the PLD with sample size ${s^ \cdot } = sl,$ where *s* is set size and *l* is the cycle number. The SPADEs ${{\hat{a}}^{{\text {SPA}}}},{{\hat{b}}^{{\text {SPA}}}}$, and ${{\hat{c}}^{{\text {SPA}}}}$ are obtained after minimizing the following function with respect to *a*, *b*, and *c*:

14 $$\begin{aligned} \rho (\omega ) = \sum \limits _{i = 1}^{{s^ \cdot } + 1} {\left| {{D_i}(\omega ) - \frac{1}{{{s^ \cdot } + 1}}} \right| }. \end{aligned}$$

The SPADEs ${{\hat{a}}^{{\text {SPA}}}},{{\hat{b}}^{{\text {SPA}}}}$, and ${{\hat{c}}^{{\text {SPA}}}}$ are obtained by solving the nonlinear equations

$$\begin{aligned}{} & {} \frac{{\partial \rho (\omega )}}{{\partial a}} = \sum \limits _{i = 1}^{{s^ \cdot } + 1} {\frac{{{D_i}(\omega ) - \frac{1}{{{s^ \cdot } + 1}}}}{{\left| {{D_i}(\omega ) - \frac{1}{{{s^ \cdot } + 1}}} \right| }}} \left[ {{\zeta _1}\left( {{x_{(i:{s^ \cdot })}}\left| \omega \right. } \right) - {\zeta _1}\left( {{x_{(i - 1:{s^ \cdot })}}\left| \omega \right. } \right) } \right] = 0, \\{} & {} \quad \frac{{\partial \rho (\omega )}}{{\partial b}} = \sum \limits _{i = 1}^{{s^ \cdot } + 1} {\frac{{{D_i}(\omega ) - \frac{1}{{{s^ \cdot } + 1}}}}{{\left| {{D_i}(\omega ) - \frac{1}{{{s^ \cdot } + 1}}} \right| }}} \left[ {{\zeta _2}\left( {{x_{(i:{s^ \cdot })}}\left| \omega \right. } \right) - {\zeta _2}\left( {{x_{(i - 1:{s^ \cdot })}}\left| \omega \right. } \right) } \right] = 0, \end{aligned}$$

and

$$\begin{aligned} \frac{{\partial \rho (\omega )}}{{\partial c}} = \sum \limits _{i = 1}^{{s^ \cdot } + 1} {\frac{{{D_i}(\omega ) - \frac{1}{{{s^ \cdot } + 1}}}}{{\left| {{D_i}(\omega ) - \frac{1}{{{s^ \cdot } + 1}}} \right| }}} \left[ {{\zeta _3}\left( {{x_{(i:{s^ \cdot })}}\left| \omega \right. } \right) - {\zeta _3}\left( {{x_{(i - 1:{s^ \cdot })}}\left| \omega \right. } \right) } \right] = 0, \end{aligned}$$

numerically, where ${\zeta _1}\left( {.\left| \omega \right. } \right) ,$ ${\zeta _2}\left( {.\left| \omega \right. } \right) ,$ and ${\zeta _3}\left( {.\left| \omega \right. } \right)$ are given in Eqs. (9), (10), and (11).

### Minimum spacing absolute-log distance

This subsection provides the SPALoDE of the unknown parameter *a*, represented by ${{\hat{a}}^{{\text {LD}}}}$, the SPALoDE of the unknown parameter *b*, represented by ${{\hat{b}}^{{\text {LD}}}}$, and the SPALoDE of the unknown parameter *c*, represented by ${{\hat{c}}^{{\text {LD}}}}$ of the PLD based on the RSS method.

Let ${X_{(1:{s^ \cdot })}},{X_{(2:{s^ \cdot })}},\ldots ,{X_{({s^ \cdot }:{s^ \cdot })}}$ are ordered RSS items from the PLD with sample size ${s^ \cdot } = sl,$ where *s* is set size and *l* is the cycle number. The SPALoDEs ${{\hat{a}}^{{\text {LD}}}},{{\hat{b}}^{{\text {LD}}}}$, and ${{\hat{c}}^{{\text {LD}}}}$ are obtained after minimizing the following function with respect to *a*, *b*, and *c*:

15 $$\begin{aligned} {\rho _1}(\omega ) = \sum \limits _{i = 1}^{{s^ \cdot } + 1} {\left| {\log \left( {{D_i}(\omega )} \right) - \log \left( {\frac{1}{{{s^ \cdot } + 1}}} \right) } \right| }. \end{aligned}$$

The following nonlinear equations can be numerically solved to obtain ${{\hat{a}}^{{\text {LD}}}},{{\hat{b}}^{{\text {LD}}}}$, and ${{\hat{c}}^{{\text {LD}}}}$ rather of using Eq. (15),

$$\begin{aligned}{} & {} \frac{{\partial {\rho _1}(\omega )}}{{\partial a}} = \sum \limits _{i = 1}^{{s^ \cdot } + 1} {\frac{{\log \left( {{D_i}(\omega )} \right) - \log \left( {\frac{1}{{{s^ \cdot } + 1}}} \right) }}{{\left| {\log \left( {{D_i}(\omega )} \right) - \log \left( {\frac{1}{{{s^ \cdot } + 1}}} \right) } \right| }}\left[ {{\zeta _1}\left( {{x_{(i:{s^ \cdot })}}\left| \omega \right. } \right) - {\zeta _1}\left( {{x_{(i - 1:{s^ \cdot })}}\left| \omega \right. } \right) } \right] } = 0, \\{} & {} \quad \frac{{\partial {\rho _1}(\omega )}}{{\partial b}} = \sum \limits _{i = 1}^{{s^ \cdot } + 1} {\frac{{\log \left( {{D_i}(\omega )} \right) - \log \left( {\frac{1}{{{s^ \cdot } + 1}}} \right) }}{{\left| {\log \left( {{D_i}(\omega )} \right) - \log \left( {\frac{1}{{{s^ \cdot } + 1}}} \right) } \right| }}\left[ {{\zeta _2}\left( {{x_{(i:{s^ \cdot })}}\left| \omega \right. } \right) - {\zeta _2}\left( {{x_{(i - 1:{s^ \cdot })}}\left| \omega \right. } \right) } \right] } = 0, \end{aligned}$$

and

$$\begin{aligned} \frac{{\partial {\rho _1}(\omega )}}{{\partial c}} = \sum \limits _{i = 1}^{{s^ \cdot } + 1} {\frac{{\log \left( {{D_i}(\omega )} \right) - \log \left( {\frac{1}{{{s^ \cdot } + 1}}} \right) }}{{\left| {\log \left( {{D_i}(\omega )} \right) - \log \left( {\frac{1}{{{s^ \cdot } + 1}}} \right) } \right| }}\left[ {{\zeta _3}\left( {{x_{(i:{s^ \cdot })}}\left| \omega \right. } \right) - {\zeta _3}\left( {{x_{(i - 1:{s^ \cdot })}}\left| \omega \right. } \right) } \right] } = 0, \end{aligned}$$

where ${\zeta _1}\left( {.\left| \omega \right. } \right) ,$ ${\zeta _2}\left( {.\left| \omega \right. } \right) ,$ and ${\zeta _3}\left( {.\left| \omega \right. } \right)$ are given in Eqs. (9), (10), and (11).

### Minimum spacing square distance

In this subsection we are concerned with the MSSDE of parameter *a*, represented by ${{\hat{a}}^{{\text {SD}}}}$, the MSSDE of parameter *b*, represented by ${{\hat{b}}^{{\text {SD}}}}$, and the MSSDE of the parameter *c*, represented by ${{\hat{c}}^{{\text {SD}}}}$ of the PLD based on RSS.

Let ${X_{(1:{s^ \cdot })}},{X_{(2:{s^ \cdot })}},\ldots ,{X_{({s^ \cdot }:{s^ \cdot })}}$ are ordered RSS items from the PLD with sample size ${s^ \cdot } = sl,$ where *s* is set size and *l* is the cycle number. The MSSDEs ${{\hat{a}}^{{\text {SD}}}},{{\hat{b}}^{{\text {SD}}}}$, and ${{\hat{c}}^{{\text {SD}}}}$ are obtained after minimizing the following function with respect to *a*, *b*, and *c*:

16 $$\begin{aligned} {\rho _2}(\omega ) = {\sum \limits _{i = 1}^{{s^ \cdot } + 1} {\left[ {{D_i}(\omega ) - \frac{1}{{{s^ \cdot } + 1}}} \right] } ^2}. \end{aligned}$$

The following nonlinear equations can be numerically solved to obtain ${{\hat{a}}^{{\text {SD}}}},{{\hat{b}}^{{\text {SD}}}}$, and ${{\hat{c}}^{{\text {SD}}}}$ rather of using Eq. (16),

$$\begin{aligned}{} & {} \frac{{\partial {\rho _2}(\omega )}}{{\partial a}} = \sum \limits _{i = 1}^{{s^ \cdot } + 1} {\left[ {{D_i}(\omega ) - \frac{1}{{{s^ \cdot } + 1}}} \right] } \left[ {{\zeta _1}\left( {{x_{(i:{s^ \cdot })}}\left| \omega \right. } \right) - {\zeta _1}\left( {{x_{(i - 1:{s^ \cdot })}}\left| \omega \right. } \right) } \right] = 0, \\{} & {} \quad \frac{{\partial {\rho _2}(\omega )}}{{\partial b}} = \sum \limits _{i = 1}^{{s^ \cdot } + 1} {\left[ {{D_i}(\omega ) - \frac{1}{{{s^ \cdot } + 1}}} \right] } \left[ {{\zeta _2}\left( {{x_{(i:{s^ \cdot })}}\left| \omega \right. } \right) - {\zeta _2}\left( {{x_{(i - 1:{s^ \cdot })}}\left| \omega \right. } \right) } \right] = 0, \end{aligned}$$

and

$$\begin{aligned} \frac{{\partial {\rho _2}(\omega )}}{{\partial c}} = \sum \limits _{i = 1}^{{s^ \cdot } + 1} {\left[ {{D_i}(\omega ) - \frac{1}{{{s^ \cdot } + 1}}} \right] } \left[ {{\zeta _3}\left( {{x_{(i:{s^ \cdot })}}\left| \omega \right. } \right) - {\zeta _3}\left( {{x_{(i - 1:{s^ \cdot })}}\left| \omega \right. } \right) } \right] = 0, \end{aligned}$$

where ${\zeta _1}\left( {.\left| \omega \right. } \right) ,$ ${\zeta _2}\left( {.\left| \omega \right. } \right) ,$ and ${\zeta _3}\left( {.\left| \omega \right. } \right)$ are given in Eqs. (9), (10), and (11).

### Minimum spacing square-log distance

Here, the MSSLDE of parameter *a*, represented by ${{\hat{a}}^{{\text {SLo}}}}$, the MSSLDE of parameter *b*, represented by ${{\hat{b}}^{{\text {SLo}}}}$, and the MSSLDE of the parameter *c*, represented by ${{\hat{c}}^{{\text {SLo}}}}$ of the PLD are determined based on RSS.

Let ${X_{(1:{s^ \cdot })}},{X_{(2:{s^ \cdot })}},\ldots ,{X_{({s^ \cdot }:{s^ \cdot })}}$ are ordered RSS items from the PLD with sample size ${s^ \cdot } = sl,$ where *s* is set size and *l* is the cycle number. The MSSLDEs ${{\hat{a}}^{{\text {SLo}}}},{{\hat{b}}^{{\text {SLo}}}}$, and ${{\hat{c}}^{{\text {SLo}}}}$ are obtained after minimizing the following function with respect to *a*, *b*, and *c*:

17 $$\begin{aligned} {\rho _3}(\omega ) = {\sum \limits _{i = 1}^{{s^ \cdot } + 1} {\left[ {\log {D_i}(\omega ) - \log \left( {\frac{1}{{{s^ \cdot } + 1}}} \right) } \right] } ^2}. \end{aligned}$$

The following nonlinear equations can be numerically solved to obtain ${{\hat{a}}^{{\text {SLo}}}}$, ${{\hat{b}}^{{\text {SLo}}}}$, and ${{\hat{c}}^{{\text {SLo}}}}$ rather of using Eq. (17),

$$\begin{aligned}{} & {} \frac{{\partial {\rho _3}(\omega )}}{{\partial a}} = \sum \limits _{i = 1}^{{s^ \cdot } + 1} {\left[ {\log {D_i}(\omega ) - \log \left( {\frac{1}{{{s^ \cdot } + 1}}} \right) } \right] \frac{1}{{{D_i}(\omega )}}\left[ {{\zeta _1}\left( {{x_{(i:{s^ \cdot })}}\left| \omega \right. } \right) - {\zeta _1}\left( {{x_{(i - 1:{s^ \cdot })}}\left| \omega \right. } \right) } \right] = 0,} \\{} & {} \quad \frac{{\partial {\rho _3}(\omega )}}{{\partial b}} = \sum \limits _{i = 1}^{{s^ \cdot } + 1} {\left[ {\log {D_i}(\omega ) - \log \left( {\frac{1}{{{s^ \cdot } + 1}}} \right) } \right] \frac{1}{{{D_i}(\omega )}}\left[ {{\zeta _2}\left( {{x_{(i:{s^ \cdot })}}\left| \omega \right. } \right) - {\zeta _2}\left( {{x_{(i - 1:{s^ \cdot })}}\left| \omega \right. } \right) } \right] = 0,} \end{aligned}$$

and

$$\begin{aligned} \frac{{\partial {\rho _3}(\omega )}}{{\partial c}} = \sum \limits _{i = 1}^{{s^ \cdot } + 1} {\left[ {\log {D_i}(\omega ) - \log \left( {\frac{1}{{{s^ \cdot } + 1}}} \right) } \right] \frac{1}{{{D_i}(\omega )}}\left[ {{\zeta _3}\left( {{x_{(i:{s^ \cdot })}}\left| \omega \right. } \right) - {\zeta _3}\left( {{x_{(i - 1:{s^ \cdot })}}\left| \omega \right. } \right) } \right] = 0,} \end{aligned}$$

where ${\zeta _1}\left( {.\left| \omega \right. } \right) ,$ ${\zeta _2}\left( {.\left| \omega \right. } \right) ,$ and ${\zeta _3}\left( {.\left| \omega \right. } \right)$ are given in Eqs. (9), (10), and (11).

### Minimum spacing Linex distance

This subsection provies the MSLNDE of parameter *a*, say ${{\hat{a}}^{{\text {SLx}}}}$, the MSLNDE of parameter *b*, say ${{\hat{b}}^{{\text {SLx}}}}$, and the MSLNDE of the parameter *c*, say ${{\hat{c}}^{{\text {SLx}}}}$ of the PLD based on RSS.

Let ${X_{(1:{s^ \cdot })}},{X_{(2:{s^ \cdot })}},\ldots ,{X_{({s^ \cdot }:{s^ \cdot })}}$ are ordered RSS items from the PLD with sample size ${s^ \cdot } = sl,$ where *s* is set size and *l* is the cycle number. The MSLNDEs ${{\hat{a}}^{{\text {SLx}}}},{{\hat{b}}^{{\text {SLx}}}}$, and ${{\hat{c}}^{{\text {SLx}}}}$ are yielded after minimizing the following function with respect to *a*, *b*, and *c*:

18 $$\begin{aligned} {\rho _4}(\omega ) = {\sum \limits _{i = 1}^{{s^ \cdot } + 1} {\left[ {{e^{{D_i}(\omega ) - \frac{1}{{{s^ \cdot } + 1}}}} - \left( {{D_i}(\omega ) - \frac{1}{{{s^ \cdot } + 1}}} \right) - 1} \right] } ^2}. \end{aligned}$$

The following nonlinear equations can be numerically solved to obtain ${{\hat{a}}^{{\text {SLx}}}}$, ${{\hat{b}}^{{\text {SLx}}}}$, and ${{\hat{c}}^{{\text {SLx}}}}$ rather of using Eq. (18),

$$\begin{aligned}{} & {} \frac{{\partial {\rho _4}(\omega )}}{{\partial a}} = {\sum \limits _{i = 1}^{{s^ \cdot } + 1} {\left[ {{e^{{D_i}(\omega ) - \frac{1}{{{s^ \cdot } + 1}}}} - \left( {{D_i}(\omega ) - \frac{1}{{{s^ \cdot } + 1}}} \right) - 1} \right] } }\\{} & {} \quad \left[ {{\zeta _1}\left( {{x_{(i:{s^ \cdot })}}\left| \omega \right. } \right) - {\zeta _1}\left( {{x_{(i - 1:{s^ \cdot })}}\left| \omega \right. } \right) } \right] \left[ {{e^{{D_i}(\omega ) - \frac{1}{{{s^ \cdot } + 1}}}} - 1} \right] = 0, \\{} & {} \quad \frac{{\partial {\rho _4}(\omega )}}{{\partial b}} = {\sum \limits _{i = 1}^{{s^ \cdot } + 1} {\left[ {{e^{{D_i}(\omega ) - \frac{1}{{{s^ \cdot } + 1}}}} - \left( {{D_i}(\omega ) - \frac{1}{{{s^ \cdot } + 1}}} \right) - 1} \right] } }\\{} & {} \quad \left[ {{\zeta _2}\left( {{x_{(i:{s^ \cdot })}}\left| \omega \right. } \right) - {\zeta _2}\left( {{x_{(i - 1:{s^ \cdot })}}\left| \omega \right. } \right) } \right] \left[ {{e^{{D_i}(\omega ) - \frac{1}{{{s^ \cdot } + 1}}}} - 1} \right] = 0, \end{aligned}$$

and

$$\begin{aligned}{} & {} \frac{{\partial {\rho _4}(\omega )}}{{\partial c}} = {\sum \limits _{i = 1}^{{s^ \cdot } + 1} {\left[ {{e^{{D_i}(\omega ) - \frac{1}{{{s^ \cdot } + 1}}}} - \left( {{D_i}(\omega ) - \frac{1}{{{s^ \cdot } + 1}}} \right) - 1} \right] } }\\{} & {} \quad \left[ {{\zeta _3}\left( {{x_{(i:{s^ \cdot })}}\left| \omega \right. } \right) - {\zeta _3}\left( {{x_{(i - 1:{s^ \cdot })}}\left| \omega \right. } \right) } \right] \left[ {{e^{{D_i}(\omega ) - \frac{1}{{{s^ \cdot } + 1}}}} - 1} \right] = 0, \end{aligned}$$

where ${\zeta _1}\left( {.\left| \omega \right. } \right) ,$ ${\zeta _2}\left( {.\left| \omega \right. } \right) ,$ and ${\zeta _3}\left( {.\left| \omega \right. } \right)$ are given in (9), (10), and (11).

## Estimation methods based on SRS

This section provides the MLE, ADE, CRME, MXPS, LSE, SPADE, SPALoDE, MSSDE, MSSLDE, and MSLNDE for the parameters *a*, *b* and *c* for the PLD based on SRS.

### Maximum likelihood estimators

Here, the MLEs ${{{\tilde{a}}}^{{\text {ML}}}}$ of *a*, ${{{\tilde{b}}}^{{\text {ML}}}}$ of *b*, and ${{{\tilde{c}}}^{{\text {ML}}}}$ of *c* for the PLD are obtained based on SRS. To get these estimators, suppose that ${x_1},{x_2},\ldots ,{x_{{s^ \cdot }}}$ is an observed SRS of size ${s^ \cdot }$ from the PLD with PDF (1). The log-LF of *a*, *b* and *c*, is given by:

$$\begin{aligned} {\ell ^{SRS}} = \sum \limits _{j = 1}^{{s^ \cdot }} {a\log \left( {{x_j}} \right) } + 2{s^ \cdot }\log (a + 1) + \sum \limits _{j = 1}^{{s^ \cdot }} {\log (b - c\ln ({x_j})) - {s^ \cdot }\log } (b + c + ab). \end{aligned}$$

When differentiating ${\ell ^{SRS}}$ with respect to *a*, *b* and *c*, we obtain the following equations:

19 $$\begin{aligned}{} & {} \frac{{\partial {\ell ^{SRS}}}}{{\partial a}} = \sum \limits _{j = 1}^{{s^ \cdot }} {\log {x_j}} + \frac{{2{s^ \cdot }}}{{a + 1}} - \frac{{{s^ \cdot }b}}{{b + c + ab}}, \end{aligned}$$

20 $$\begin{aligned}{} & {} \frac{{\partial {\ell ^{SRS}}}}{{\partial b}} = \sum \limits _{j = 1}^{{s^ \cdot }} {\frac{1}{{b - c\ln ({x_j})}} - } \frac{{{s^ \cdot }(1 + a)}}{{b + c + ab}}, \end{aligned}$$

and

21 $$\begin{aligned} \frac{{\partial {\ell ^{SRS}}}}{{\partial c}} = \sum \limits _{j = 1}^{{s^ \cdot }} {\frac{{ - \ln ({x_j})}}{{b - c\ln ({x_j})}} - \frac{{{s^ \cdot }}}{{b + c + ab}}}. \end{aligned}$$

Using the statistical software Mathematica, the nonlinear equations (19)–(21) may be solved numerically after setting them equal to zero, to obtain the MLEs ${{{\tilde{a}}}^{{\text {ML}}}},{{{\tilde{b}}}^{{\text {ML}}}},$ and ${{{\tilde{c}}}^{{\text {ML}}}}$ of *a*, *b*, and *c*, respectively.

### Anderson–Darling estimators

In this subsection, the ADE of parameter *a*, say ${{{\tilde{a}}}^{{\text {AD}}}}$, ADE of parameter *b*, say ${{{\tilde{b}}}^{{\text {AD}}}}$, and the ADE of parameter *c*, say ${{{\tilde{c}}}^{{\text {AD}}}}$ of the PLD are obtained using SRS.

Let ${X_{(1)}},{X_{(2)}},\ldots ,{X_{({s^ \cdot })}}$ be ordered items from SRS following PLD with sample size ${s^ \cdot }$. Thus, the ADEs ${{{\tilde{a}}}^{{\text {AD}}}},{{{\tilde{b}}}^{{\text {AD}}}},{{{\tilde{c}}}^{{\text {AD}}}}$ of *a*, *b*, and *c* are derived by minimizing the function

22 $$\begin{aligned} {{\text {A}}_{\text {1}}}{\text {(}}\omega {\text {)}} = - {s^ \cdot } - \frac{1}{{{s^ \cdot }}}\sum \limits _{j = 1}^{{s^ \cdot }} {(2j - 1)\left\{ {\log F\left( {{x_{(j)}}\left| \omega \right. } \right) + \log {\bar{F}}\left( {{x_{({s^ \cdot } - j + 1)}}\left| \omega \right. } \right) } \right\} } . \end{aligned}$$

Alternatively, the ADEs of the PLD can be obtained by solving the subsequent non-linear equations in place of Eq. (22):

$$\begin{aligned}{} & {} \frac{{\partial {{\text {A}}_{\text {1}}}{\text {(}}\omega {\text {)}}}}{{\partial a}} = \sum \limits _{j = 1}^{{s^ \cdot }} {(2j - 1)\left\{ {\frac{{\,{\zeta _1}\left( {{x_{(j)}}\left| \omega \right. } \right) }}{{F\left( {{x_{(j)}}\left| \omega \right. } \right) }} - \frac{{{\zeta _1}\left( {{x_{({s^ \cdot } - j + 1)}}\left| \omega \right. } \right) }}{{{\bar{F}}\left( {{x_{({s^ \cdot } - j + 1)}}\left| \omega \right. } \right) }}} \right\} = 0,} \\{} & {} \quad \frac{{\partial {{\text {A}}_{\text {1}}}{\text {(}}\omega {\text {)}}}}{{\partial b}} = \sum \limits _{j = 1}^{{s^ \cdot }} {(2j - 1)\left\{ {\frac{{\,{\zeta _2}\left( {{x_{(j)}}\left| \omega \right. } \right) }}{{F\left( {{x_{(j)}}\left| \omega \right. } \right) }} - \frac{{{\zeta _2}\left( {{x_{({s^ \cdot } - j + 1)}}\left| \omega \right. } \right) }}{{{\bar{F}}\left( {{x_{({s^ \cdot } - j + 1)}}\left| \omega \right. } \right) }}} \right\} = 0,} \end{aligned}$$

and

$$\begin{aligned} \frac{{\partial {{\text {A}}_{\text {1}}}{\text {(}}\omega {\text {)}}}}{{\partial c}} = \sum \limits _{j = 1}^{{s^ \cdot }} {(2j - 1)\left\{ {\frac{{\,{\zeta _3}\left( {{x_{(j)}}\left| \omega \right. } \right) }}{{F\left( {{x_{(j)}}\left| \omega \right. } \right) }} - \frac{{{\zeta _3}\left( {{x_{({s^ \cdot } - j + 1)}}\left| \omega \right. } \right) }}{{{\bar{F}}\left( {{x_{({s^ \cdot } - j + 1)}}\left| \omega \right. } \right) }}} \right\} = 0,} \end{aligned}$$

where ${\zeta _1}\left( {.\left| \omega \right. } \right) ,$ ${\zeta _2}\left( {.\left| \omega \right. } \right) ,$ and ${\zeta _3}\left( {.\left| \omega \right. } \right)$ are given in Eqs. (9), (10), and (11) with ordered sample ${x_{(j)}}$ and ${x_{({s^ \cdot } - j + 1)}}.$

### Cramer–von-Mises estimators

Here, we get the CRME of the parameter *a*, denoted by ${{{\tilde{a}}}^{{\text {CR}}}}$, the CRME of parameter *b*, denoted by ${{{\tilde{b}}}^{{\text {CR}}}}$, and the CRME of parameter *c*, denoted by ${{{\tilde{c}}}^{{\text {CR}}}}$, of the PLD are covered using SRS method.

Let ${X_{(1)}},{X_{(2)}},\ldots ,{X_{({s^ \cdot })}}$ are ordered SRS items taken from the PLD with sample size ${s^ \cdot }$. The CRMEs ${{{\tilde{a}}}^{{\text {CR}}}}$, ${{{\tilde{b}}}^{{\text {CR}}}}$, and ${{{\tilde{c}}}^{{\text {CR}}}}$ of *a*, *b*, and *c* are derived by minimizing the function

23 $$\begin{aligned} {C_1}(\omega ) = \frac{1}{{12{s^ \cdot }}} + \sum \limits _{j = 1}^{{s^ \cdot }} {{{\left\{ {F\left( {{x_{(j)}}\left| \omega \right. } \right) - \frac{{2j - 1}}{{2{s^ \cdot }}}} \right\} }^2}}. \end{aligned}$$

The following non-linear equations can be solved to get the CRMEs instead of using Eq. (23)

$$\begin{aligned}{} & {} \frac{{\partial {C_1}(\omega )}}{{\partial a}} = \sum \limits _{j = 1}^{{s^ \cdot }} {\left\{ {F\left( {{x_{(j)}}\left| \omega \right. } \right) - \frac{{2j - 1}}{{2{s^ \cdot }}}} \right\} {\zeta _1}\left( {{x_{(j)}}\left| \omega \right. } \right) = 0,} \\{} & {} \quad \frac{{\partial {C_1}(\omega )}}{{\partial b}} = \sum \limits _{j = 1}^{{s^ \cdot }} {\left\{ {F\left( {{x_{(j)}}\left| \omega \right. } \right) - \frac{{2j - 1}}{{2{s^ \cdot }}}} \right\} {\zeta _2}\left( {{x_{(j)}}\left| \omega \right. } \right) = 0,} \end{aligned}$$

and

$$\begin{aligned} \frac{{\partial {C_1}(\omega )}}{{\partial c}} = \sum \limits _{j = 1}^{{s^ \cdot }} {\left\{ {F\left( {{x_{(j)}}\left| \omega \right. } \right) - \frac{{2j - 1}}{{2{s^ \cdot }}}} \right\} {\zeta _3}\left( {{x_{(j)}}\left| \omega \right. } \right) = 0,} \end{aligned}$$

where ${\zeta _1}\left( {.\left| \omega \right. } \right) ,$ ${\zeta _2}\left( {.\left| \omega \right. } \right) ,$ and ${\zeta _3}\left( {.\left| \omega \right. } \right)$ are given in Eqs. (9), (10), and (11) with ordered sample ${x_{(j)}}$.

### Maximum product of spacings estimators

This subsection presents the MXPSE of the PLD using SRS for parameter *a*, indicated by ${{{\tilde{a}}}^{{\text {MP}}}}$, the MXPSE of parameter *b*, indicated by ${{{\tilde{b}}}^{{\text {MP}}}}$, and the MXPSE of parameter *c*, indicated by ${{{\tilde{c}}}^{{\text {MP}}}}$.

Let ${X_{(1)}},{X_{(2)}},\ldots ,{X_{({s^ \cdot })}}$ are ordered SRS items taken from the PLD with sample size ${s^ \cdot }$. The MXPSEs ${{{\tilde{a}}}^{{\text {MP}}}},{{{\tilde{b}}}^{{\text {MP}}}}$, and ${{{\tilde{c}}}^{{\text {MP}}}}$ of *a*, *b*, and *c* are found by maximizing the geometric mean of the spacing, which is obtained by maximizing

$$\begin{aligned} {{\text {M}}_{\text {1}}} = \frac{1}{{{s^ \cdot } + 1}}\sum \limits _{j = 1}^{{s^ \cdot } + 1} {\ln \left[ {D_j^ * (\omega )} \right] }, \end{aligned}$$

where $D_j^ * (\omega ) = F\left( {{x_{(j)}}\left| \omega \right. } \right) - F\left( {{x_{(j - 1)}}\left| \omega \right. } \right) ,\,\,\,\,\,j = 1,2,\ldots ,{s^ \cdot } + 1,$ $F\left( {{x_{(0)}}\left| \omega \right. } \right) = 0,\,\,\,\,F\left( {{x_{({s^ \cdot } + 1)}}\left| \omega \right. } \right) = 1,$ such that $\sum \limits _{j = 1}^{{s^ \cdot } + 1} {D_j^ * (\omega )} = 1.$ The MXPSEs ${{{\tilde{a}}}^{{\text {MP}}}},{{{\tilde{b}}}^{{\text {MP}}}},{{{\tilde{c}}}^{{\text {MP}}}}$ are provided by numerically solving the equations

$$\begin{aligned}{} & {} \frac{{\partial {{\text {M}}_{\text {1}}}}}{{\partial a}} = \frac{1}{{1 + {s^ \cdot }}}\sum \limits _{j = 1}^{{s^ \cdot } + 1} {\frac{1}{{D_j^ * (\omega )}}} \left[ {{\zeta _1}\left( {{x_{(j)}}\left| \omega \right. } \right) - {\zeta _1}\left( {{x_{(j - 1)}}\left| \omega \right. } \right) } \right] = 0, \\{} & {} \quad \frac{{\partial {{\text {M}}_{\text {1}}}}}{{\partial b}} = \frac{1}{{1 + {s^ \cdot }}}\sum \limits _{j = 1}^{{s^ \cdot } + 1} {\frac{1}{{D_j^ * (\omega )}}} \left[ {{\zeta _2}\left( {{x_{(j)}}\left| \omega \right. } \right) - {\zeta _2}\left( {{x_{(j - 1)}}\left| \omega \right. } \right) } \right] = 0, \end{aligned}$$

and

$$\begin{aligned} \frac{{\partial {{\text {M}}_{\text {1}}}}}{{\partial c}} = \frac{1}{{1 + {s^ \cdot }}}\sum \limits _{j = 1}^{{s^ \cdot } + 1} {\frac{1}{{D_j^ * (\omega )}}} \left[ {{\zeta _3}\left( {{x_{(j)}}\left| \omega \right. } \right) - {\zeta _3}\left( {{x_{(j - 1)}}\left| \omega \right. } \right) } \right] = 0, \end{aligned}$$

where ${\zeta _1}\left( {.\left| \omega \right. } \right) ,$ ${\zeta _2}\left( {.\left| \omega \right. } \right) ,$ and ${\zeta _3}\left( {.\left| \omega \right. } \right)$ are given in Eqs. (9), (10), and (11).

### Least squares estimators

Here, we use the SRS method to produce the LSE of the parameter *a*, denoted by ${{{\tilde{a}}}^{{\text {LS}}}}$, the LSE of parameter *b*, denoted by ${{{\tilde{b}}}^{{\text {LS}}}}$, and the LSE of parameter *c*, denoted by ${{{\tilde{c}}}^{{\text {LS}}}}$, of the PLD.

Let ${X_{(1)}},{X_{(2)}},\ldots ,{X_{({s^ \cdot })}}$ be ordered SRS items taken from the PLD with sample size ${s^ \cdot }$. The LSEs ${{{\tilde{a}}}^{{\text {LS}}}},{{{\tilde{b}}}^{{\text {LS}}}}$, and ${{{\tilde{c}}}^{{\text {LS}}}}$ are obtained after minimizing the following function with respect to the unknown parameters *a*, *b*, and *c*:

$$\begin{aligned} {{\text {L}}_{\text {1}}} = \sum \limits _{j = 1}^{{s^ \cdot }} {{{\left[ {F\left( {{x_{(j)}}\left| \omega \right. } \right) - \frac{j}{{{s^ \cdot } + 1}}} \right] }^2}}. \end{aligned}$$

Alternately, the LSEs ${{{\tilde{a}}}^{{\text {LS}}}},{{{\tilde{b}}}^{{\text {LS}}}}$, and ${{{\tilde{c}}}^{{\text {LS}}}}$ are acquired by minimizing the following equations:

$$\begin{aligned}{} & {} \frac{{\partial {{\text {L}}_{\text {1}}}}}{{\partial a}} = \sum \limits _{j = 1}^{{s^ \cdot }} {\left[ {F\left( {{x_{(j)}}\left| \omega \right. } \right) - \frac{j}{{{s^ \cdot } + 1}}} \right] } \,\,\,{\zeta _1}\left( {{x_{(j)}}\left| \omega \right. } \right) = 0, \\{} & {} \quad \frac{{\partial {{\text {L}}_{\text {1}}}}}{{\partial b}} = \sum \limits _{j = 1}^{{s^ \cdot }} {\left[ {F\left( {{x_{(j)}}\left| \omega \right. } \right) - \frac{j}{{{s^ \cdot } + 1}}} \right] } \,\,\,{\zeta _2}\left( {{x_{(j)}}\left| \omega \right. } \right) = 0, \end{aligned}$$

and

$$\begin{aligned} \frac{{\partial {{\text {L}}_{\text {1}}}}}{{\partial c}} = \sum \limits _{j = 1}^{{s^ \cdot }} {\left[ {F\left( {{x_{(j)}}\left| \omega \right. } \right) - \frac{j}{{{s^ \cdot } + 1}}} \right] } \,\,\,{\zeta _3}\left( {{x_{(j)}}\left| \omega \right. } \right) = 0, \end{aligned}$$

where ${\zeta _1}\left( {.\left| \omega \right. } \right) ,$ ${\zeta _2}\left( {.\left| \omega \right. } \right) ,$ and ${\zeta _3}\left( {.\left| \omega \right. } \right)$ are given in (9), (10), and (11).

### Minimum spacing absolute distance estimators

This subsection provides the SPADE of the unknown parameter *a*, represented by ${{{\tilde{a}}}^{{\text {SPA}}}}$, the SPADE of the unknown parameter *b*, represented by ${{{\tilde{b}}}^{{\text {SPA}}}}$, and the SPADE of the unknown parameter *c*, represented by ${{{\tilde{c}}}^{{\text {SPA}}}}$ of the PLD based on the SRS method.

Let ${X_{(1)}},{X_{(2)}},\ldots ,{X_{({s^ \cdot })}}$ be ordered SRS items taken from the PLD with sample size ${s^ \cdot }$. The SPADEs ${{{\tilde{a}}}^{{\text {SPA}}}},{{{\tilde{b}}}^{{\text {SPA}}}}$, and ${{{\tilde{c}}}^{{\text {SPA}}}}$ are obtained after minimizing the following function with respect to *a*, *b*, and *c*:

$$\begin{aligned} {\rho ^ * }(\omega ) = \sum \limits _{j = 1}^{{s^ \cdot } + 1} {\left| {D_j^ * (\omega ) - \frac{1}{{{s^ \cdot } + 1}}} \right| }. \end{aligned}$$

Alternately, the following nonlinear equations can be solved numerically to yield the SPADEs ${{{\tilde{a}}}^{{\text {SPA}}}},{{{\tilde{b}}}^{{\text {SPA}}}}$, and ${{{\tilde{c}}}^{{\text {SPA}}}}$

$$\begin{aligned}{} & {} \frac{{\partial {\rho ^ * }(\omega )}}{{\partial a}} = \sum \limits _{j = 1}^{{s^ \cdot } + 1} {\frac{{D_j^ * (\omega ) - \frac{1}{{{s^ \cdot } + 1}}}}{{\left| {D_j^ * (\omega ) - \frac{1}{{{s^ \cdot } + 1}}} \right| }}} \left[ {{\zeta _1}\left( {{x_{(j)}}\left| \omega \right. } \right) - {\zeta _1}\left( {{x_{(j - 1)}}\left| \omega \right. } \right) } \right] = 0, \\{} & {} \quad \frac{{\partial {\rho ^ * }(\omega )}}{{\partial b}} = \sum \limits _{j = 1}^{{s^ \cdot } + 1} {\frac{{D_j^ * (\omega ) - \frac{1}{{{s^ \cdot } + 1}}}}{{\left| {D_j^ * (\omega ) - \frac{1}{{{s^ \cdot } + 1}}} \right| }}} \left[ {{\zeta _2}\left( {{x_{(j)}}\left| \omega \right. } \right) - {\zeta _2}\left( {{x_{(j - 1)}}\left| \omega \right. } \right) } \right] = 0, \end{aligned}$$

and

$$\begin{aligned} \frac{{\partial {\rho ^ * }(\omega )}}{{\partial c}} = \sum \limits _{j = 1}^{{s^ \cdot } + 1} {\frac{{D_j^ * (\omega ) - \frac{1}{{{s^ \cdot } + 1}}}}{{\left| {D_j^ * (\omega ) - \frac{1}{{{s^ \cdot } + 1}}} \right| }}} \left[ {{\zeta _3}\left( {{x_{(j)}}\left| \omega \right. } \right) - {\zeta _3}\left( {{x_{(j - 1)}}\left| \omega \right. } \right) } \right] = 0, \end{aligned}$$

where ${\zeta _1}\left( {.\left| \omega \right. } \right) ,$ ${\zeta _2}\left( {.\left| \omega \right. } \right) ,$ and ${\zeta _3}\left( {.\left| \omega \right. } \right)$ are given in Eqs. (9), (10), and (11).

### Minimum spacing absolute-log distance estimators

Here, we determine the SPALoDE of the unknown parameter *a*, represented by ${{{\tilde{a}}}^{{\text {LD}}}}$, the SPALoDE of the unknown parameter *b*, represented by ${{{\tilde{b}}}^{{\text {LD}}}}$, and the SPALoDE of the unknown parameter *c*, represented by ${{{\tilde{c}}}^{{\text {LD}}}}$ of the PLD based on the SRS technique.

Let ${X_{(1)}},{X_{(2)}},\ldots ,{X_{({s^ \cdot })}}$ are ordered SRS items taken from the PLD with sample size ${s^ \cdot }$. The SPALoDEs ${{{\tilde{a}}}^{{\text {LD}}}},{{{\tilde{b}}}^{{\text {LD}}}}$, and ${{{\tilde{c}}}^{{\text {LD}}}}$ are obtained after minimizing the following function with respect to *a*, *b*, and *c*:

24 $$\begin{aligned} \rho _{_1}^ * (\omega ) = \sum \limits _{j = 1}^{{s^ \cdot } + 1} {\left| {\log \left( {D_{_j}^ * (\omega )} \right) - \log \left( {\frac{1}{{{s^ \cdot } + 1}}} \right) } \right| }. \end{aligned}$$

The following nonlinear equations can be numerically solved to obtain ${{{\tilde{a}}}^{{\text {LD}}}},{{{\tilde{b}}}^{{\text {LD}}}}$, and ${{{\tilde{c}}}^{{\text {LD}}}}$ rather of using Eq. (24),

$$\begin{aligned}{} & {} \frac{{\partial \rho _{_1}^ * (\omega )}}{{\partial a}} = \sum \limits _{j = 1}^{{s^ \cdot } + 1} {\frac{{\log \left( {D_j^ * (\omega )} \right) - \log \left( {\frac{1}{{{s^ \cdot } + 1}}} \right) }}{{\left| {\log \left( {D_j^ * (\omega )} \right) - \log \left( {\frac{1}{{{s^ \cdot } + 1}}} \right) } \right| }}\left[ {{\zeta _1}\left( {{x_{(j)}}\left| \omega \right. } \right) - {\zeta _1}\left( {{x_{(j - 1)}}\left| \omega \right. } \right) } \right] } = 0, \\{} & {} \quad \frac{{\partial \rho _{_1}^ * (\omega )}}{{\partial b}} = \sum \limits _{j = 1}^{{s^ \cdot } + 1} {\frac{{\log \left( {D_j^ * (\omega )} \right) - \log \left( {\frac{1}{{{s^ \cdot } + 1}}} \right) }}{{\left| {\log \left( {D_j^ * (\omega )} \right) - \log \left( {\frac{1}{{{s^ \cdot } + 1}}} \right) } \right| }}\left[ {{\zeta _2}\left( {{x_{(j)}}\left| \omega \right. } \right) - {\zeta _2}\left( {{x_{(j - 1)}}\left| \omega \right. } \right) } \right] } = 0, \end{aligned}$$

and

$$\begin{aligned} \frac{{\partial \rho _{_1}^ * (\omega )}}{{\partial c}} = \sum \limits _{j = 1}^{{s^ \cdot } + 1} {\frac{{\log \left( {D_j^ * (\omega )} \right) - \log \left( {\frac{1}{{{s^ \cdot } + 1}}} \right) }}{{\left| {\log \left( {D_j^ * (\omega )} \right) - \log \left( {\frac{1}{{{s^ \cdot } + 1}}} \right) } \right| }}\left[ {{\zeta _3}\left( {{x_{(j)}}\left| \omega \right. } \right) - {\zeta _3}\left( {{x_{(j - 1)}}\left| \omega \right. } \right) } \right] } = 0, \end{aligned}$$

where ${\zeta _1}\left( {.\left| \omega \right. } \right) ,$ ${\zeta _2}\left( {.\left| \omega \right. } \right) ,$ and ${\zeta _3}\left( {.\left| \omega \right. } \right)$ are given in Eqs. (9), (10), and (11).

### Minimum spacing square distance estimators

Here, we determine the MSSDE of the unknown parameter *a*, represented by ${{{\tilde{a}}}^{{\text {SD}}}}$, the MSSDE of the unknown parameter *b*, represented by ${{{\tilde{b}}}^{{\text {SD}}}}$, and the MSSDE of the unknown parameter *c*, represented by ${{{\tilde{c}}}^{{\text {SD}}}}$ of the PLD based on SRS.

Let ${X_{(1)}},{X_{(2)}},\ldots ,{X_{({s^ \cdot })}}$ are ordered SRS items taken from the PLD with sample size ${s^ \cdot }$. The MSSDEs ${{{\tilde{a}}}^{{\text {SD}}}},{{{\tilde{b}}}^{{\text {SD}}}}$, and ${{{\tilde{c}}}^{{\text {SD}}}}$ are obtained after minimizing the following function with respect to *a*, *b*, and *c*:

25 $$\begin{aligned} \rho _{_2}^ * (\omega ) = {\sum \limits _{j = 1}^{{s^ \cdot } + 1} {\left[ {D_j^ * (\omega ) - \frac{1}{{{s^ \cdot } + 1}}} \right] } ^2}. \end{aligned}$$

The following nonlinear equations can be numerically solved to obtain ${{{\tilde{a}}}^{{\text {SD}}}},{{{\tilde{b}}}^{{\text {SD}}}}$, and ${{{\tilde{c}}}^{{\text {SD}}}}$ rather of use Eq. (25),

$$\begin{aligned}{} & {} \frac{{\partial \rho _{_2}^ * (\omega )}}{{\partial a}} = \sum \limits _{j = 1}^{{s^ \cdot } + 1} {\left[ {D_j^ * (\omega ) - \frac{1}{{{s^ \cdot } + 1}}} \right] } \left[ {{\zeta _1}\left( {{x_{(j)}}\left| \omega \right. } \right) - {\zeta _1}\left( {{x_{(j - 1)}}\left| \omega \right. } \right) } \right] = 0, \\{} & {} \quad \frac{{\partial \rho _{_2}^ * (\omega )}}{{\partial b}} = \sum \limits _{j = 1}^{{s^ \cdot } + 1} {\left[ {D_j^ * (\omega ) - \frac{1}{{{s^ \cdot } + 1}}} \right] } \left[ {{\zeta _2}\left( {{x_{(j)}}\left| \omega \right. } \right) - {\zeta _2}\left( {{x_{(j - 1)}}\left| \omega \right. } \right) } \right] = 0, \end{aligned}$$

and

$$\begin{aligned} \frac{{\partial \rho _{_2}^ * (\omega )}}{{\partial c}} = \sum \limits _{j = 1}^{{s^ \cdot } + 1} {\left[ {D_j^ * (\omega ) - \frac{1}{{{s^ \cdot } + 1}}} \right] } \left[ {{\zeta _3}\left( {{x_{(j)}}\left| \omega \right. } \right) - {\zeta _3}\left( {{x_{(j - 1)}}\left| \omega \right. } \right) } \right] = 0, \end{aligned}$$

where ${\zeta _1}\left( {.\left| \omega \right. } \right) ,$ ${\zeta _2}\left( {.\left| \omega \right. } \right) ,$ and ${\zeta _3}\left( {.\left| \omega \right. } \right)$ are given in Eqs. (9), (10), and (11).

### Minimum spacing square-log distance estimators

Here, the MSSLDE of parameter *a*, represented by ${{{\tilde{a}}}^{{\text {SLo}}}}$, the MSSLDE of parameter *b*, represented by ${{{\tilde{b}}}^{{\text {SLo}}}}$, and the MSSLDE of parameter *c*, represented by ${{{\tilde{c}}}^{{\text {SLo}}}}$ of the PLD are determined based on SRS.

Let ${X_{(1)}},{X_{(2)}},\ldots ,{X_{({s^ \cdot })}}$ are ordered SRS items taken from the PLD with sample size ${s^ \cdot }$. The MSSLDEs ${{{\tilde{a}}}^{{\text {SLo}}}},{{{\tilde{b}}}^{{\text {SLo}}}}$, and ${{{\tilde{c}}}^{{\text {SLo}}}}$ are obtained after minimizing the following function with respect to *a*, *b*, and *c*:

26 $$\begin{aligned} \rho _{_3}^ * (\omega ) = {\sum \limits _{j = 1}^{{s^ \cdot } + 1} {\left[ {\log D_j^ * (\omega ) - \log \left( {\frac{1}{{{s^ \cdot } + 1}}} \right) } \right] } ^2}. \end{aligned}$$

The following nonlinear equations can be numerically solved to obtain ${{{\tilde{a}}}^{{\text {SLo}}}},{{{\tilde{b}}}^{{\text {SLo}}}}$, and ${{{\tilde{c}}}^{{\text {SLo}}}}$ rather of using Eq. (26),

$$\begin{aligned}{} & {} \frac{{\partial \rho _{_3}^ * (\omega )}}{{\partial a}} = \sum \limits _{j = 1}^{{s^ \cdot } + 1} {\left[ {\log D_j^ * (\omega ) - \log \left( {\frac{1}{{{s^ \cdot } + 1}}} \right) } \right] } \frac{1}{{D_j^ * (\omega )}}\left[ {{\zeta _1}\left( {{x_{(j)}}\left| \omega \right. } \right) - {\zeta _1}\left( {{x_{(j - 1)}}\left| \omega \right. } \right) } \right] = 0, \\{} & {} \quad \frac{{\partial \rho _{_3}^ * (\omega )}}{{\partial b}} = \sum \limits _{j = 1}^{{s^ \cdot } + 1} {\left[ {\log D_j^ * (\omega ) - \log \left( {\frac{1}{{{s^ \cdot } + 1}}} \right) } \right] } \frac{1}{{D_j^ * (\omega )}}\left[ {{\zeta _2}\left( {{x_{(j)}}\left| \omega \right. } \right) - {\zeta _2}\left( {{x_{(j - 1)}}\left| \omega \right. } \right) } \right] = 0, \end{aligned}$$

and

$$\begin{aligned} \frac{{\partial \rho _{_3}^ * (\omega )}}{{\partial c}} = \sum \limits _{j = 1}^{{s^ \cdot } + 1} {\left[ {\log D_j^ * (\omega ) - \log \left( {\frac{1}{{{s^ \cdot } + 1}}} \right) } \right] } \frac{1}{{D_j^ * (\omega )}}\left[ {{\zeta _3}\left( {{x_{(j)}}\left| \omega \right. } \right) - {\zeta _3}\left( {{x_{(j - 1)}}\left| \omega \right. } \right) } \right] = 0, \end{aligned}$$

where ${\zeta _1}\left( {.\left| \omega \right. } \right) ,$ ${\zeta _2}\left( {.\left| \omega \right. } \right) ,$ and ${\zeta _3}\left( {.\left| \omega \right. } \right)$ are given in Eqs. (9), (10), and (11).

### Minimum spacing Linex distance estimators

This subsection provides the MSLNDE of parameter *a*, say ${{{\tilde{a}}}^{{\text {SLx}}}}$, the MSLNDE of parameter *b*, say ${{{\tilde{b}}}^{{\text {SLx}}}}$, and the MSLNDE of parameter *c*, say ${{{\tilde{c}}}^{{\text {SLx}}}}$ of the PLD based on SRS.

Let ${X_{(1)}},{X_{(2)}},\ldots ,{X_{({s^ \cdot })}}$ are ordered SRS items taken from the PLD with sample size ${s^ \cdot }$. The MSLNDEs ${{{\tilde{a}}}^{{\text {SLx}}}},{{{\tilde{b}}}^{{\text {SLx}}}}$, and ${{{\tilde{c}}}^{{\text {SLx}}}}$ are obtained after minimizing the following function with respect to *a*, *b*, and *c*:

27 $$\begin{aligned} \rho _4^ * (\omega ) = {\sum \limits _{j = 1}^{{s^ \cdot } + 1} {\left[ {{e^{D_j^ * (\omega ) - \frac{1}{{{s^ \cdot } + 1}}}} - \left( {D_j^ * (\omega ) - \frac{1}{{{s^ \cdot } + 1}}} \right) - 1} \right] } ^2}. \end{aligned}$$

The following nonlinear equations can be numerically solved to obtain ${{\hat{a}}^{{\text {SLx}}}},{{\hat{b}}^{{\text {SLx}}}}$, and ${{\hat{c}}^{{\text {SLx}}}}$ rather of using Eq. (27),

$$\begin{aligned}{} & {} \frac{{\partial \rho _4^ * (\omega )}}{{\partial a}} = \sum \limits _{j = 1}^{{s^ \cdot } + 1} {\left[ {{e^{D_j^ * (\omega ) - \frac{1}{{{s^ \cdot } + 1}}}} - \left( {D_j^ * (\omega ) - \frac{1}{{{s^ \cdot } + 1}}} \right) - 1} \right] } \left[ {{\zeta _1}\left( {{x_{(j)}}\left| \omega \right. } \right) - {\zeta _1}\left( {{x_{(j - 1)}}\left| \omega \right. } \right) } \right] \left[ {{e^{D_j^ * (\omega ) - \frac{1}{{{s^ \cdot } + 1}}}} - 1} \right] = 0, \\{} & {} \quad \frac{{\partial \rho _4^ * (\omega )}}{{\partial b}} = \sum \limits _{j = 1}^{{s^ \cdot } + 1} {\left[ {{e^{D_j^ * (\omega ) - \frac{1}{{{s^ \cdot } + 1}}}} - \left( {D_j^ * (\omega ) - \frac{1}{{{s^ \cdot } + 1}}} \right) - 1} \right] } \left[ {{\zeta _2}\left( {{x_{(j)}}\left| \omega \right. } \right) - {\zeta _2}\left( {{x_{(j - 1)}}\left| \omega \right. } \right) } \right] \left[ {{e^{D_j^ * (\omega ) - \frac{1}{{{s^ \cdot } + 1}}}} - 1} \right] = 0, \end{aligned}$$

and

$$\begin{aligned} \frac{{\partial \rho _4^ * (\omega )}}{{\partial c}} = \sum \limits _{j = 1}^{{s^ \cdot } + 1} {\left[ {{e^{D_j^ * (\omega ) - \frac{1}{{{s^ \cdot } + 1}}}} - \left( {D_j^ * (\omega ) - \frac{1}{{{s^ \cdot } + 1}}} \right) - 1} \right] } \left[ {{\zeta _3}\left( {{x_{(j)}}\left| \omega \right. } \right) - {\zeta _3}\left( {{x_{(j - 1)}}\left| \omega \right. } \right) } \right] \left[ {{e^{D_j^ * (\omega ) - \frac{1}{{{s^ \cdot } + 1}}}} - 1} \right] = 0, \end{aligned}$$

where ${\zeta _1}\left( {.\left| \omega \right. } \right) ,$ ${\zeta _2}\left( {.\left| \omega \right. } \right) ,$ and ${\zeta _3}\left( {.\left| \omega \right. } \right)$ are given in Eqs. (9), (10), and (11).

## Numerical simulation

The variety of estimation techniques described in this study is examined in this section. By creating random data sets produced from the proposed model, the effectiveness of these methods in identifying model parameters is evaluated. After that, these data sets go through ranking processes, and the estimation techniques are used to identify which one is the best. The simulation operates on the assumption of a flawless ranking, as elaborated below:

- We compute the corresponding sample sizes $s^ \cdot = sl$, resulting in $s^ \cdot = sl = 30, 75, 150, 250, 400$. This allows us to create an RSS from the suggested model with a fixed set size of $s = 5$ and changing cycle numbers $l = 6, 15, 30, 50, 80$.
- We generate SRS from the suggested model using the specified sample sizes $s^ \cdot = 15, 50, 120, 200, 300,450$.
- Using the actual parameter values (*a*, *b*, *c*), we derive a set of estimates for each sample size.
- To assess the efficacy of the estimation methods, six metrics are utilized, comprising:
  - The average of absolute bias (BIAS), computed by the formula: $|\text {Bias}(\widehat{\pmb \omega })| = \frac{1}{H}\sum _{i=1}^{H}|\widehat{\pmb \omega }-\pmb \omega |$.
  - The mean squared error (MSE), determined as follows: $\text {MSE} = \frac{1}{H}\sum _{i=1}^{H}(\widehat{\pmb \omega }-\pmb \omega )^2$.
  - The mean absolute relative error (MRE), evaluated using the expression: $\text {MRE} = \frac{1}{H}\sum _{i=1}^{H}|\widehat{\pmb \omega }-\pmb \omega |/\pmb \omega$.
  - The average absolute difference, denoted as $D_{\text {abs}}$, calculated by: $D_{\text {abs}} = \frac{1}{s^ \cdot H}\sum _{i=1}^{H}\sum _{j=1}^{s^ \cdot }|F(x_{ij}; \pmb \omega )-F(x_{ij};\widehat{ \pmb \omega })|$, where $F(x;\pmb \omega )=F(x)$ and $x_{ij}$ represent values obtained at the *i*-th iteration sample and *j*-th component of this sample.
  - The maximum absolute difference, represented by $D_{\text {max}}$, obtained from: $D_{\text {max}} = \frac{1}{H}\sum _{i=1}^{H}\max \limits _{j=1,\ldots ,n} |F(x_{ij}; \pmb \omega )-F(x_{ij};\widehat{ \pmb \omega })|$.
  - The average squared absolute error (ASAE), computed as: $\text {ASAE} = \frac{1}{H}\sum _{i=1}^{H}\frac{|x_{(i)}-\hat{x}_{(i)}|}{x_{(n)}-x_{(1)}}$, where $x_{(i)}$ denotes the ascending ordered observations, and $\pmb \omega =(a,b,c)$.
- The metrics delineated in the preceding step function as impartial standards for appraising the precision and dependability of the estimated parameters. Employing these assessment criteria facilitates a thorough evaluation of the efficacy of the estimation methods. This evaluative procedure yields significant insights into the effectiveness and suitability of these methods for the specific model in question.
- This approach can be repeated several times to provide a solid and trustworthy assessment of the estimation methods. By ensuring consistency and clarity in the performance findings, this repeated assessment improves our comprehension of how successful these strategies are in parameter estimation for the model.
- The assessment metrics related to RSS and SRS are shown in Suppl Tables 1–10 (see Suppl Appendix). These tables provide a thorough summary of the outcomes attained. The numbers in these tables represent the relative effectiveness of each strategy out of all the estimation techniques that were looked at. Reduced values indicate better performance than the examined estimation methods. These tables are crucial for evaluating the relative merits and efficacy of the various estimation methods.
- The MSE ratio of SRS to RSS is shown in Suppl Table 11, which facilitates the evaluation of the MSE performance of different sampling techniques and provides information on their efficiency.
- Suppl Tables 12 and 13 for SRS and RSS (see Suppl Appendix), respectively, give comprehensive rankings, including partial and total ranks. These ranking tables thoroughly analyze each estimation technique’s relative efficacy and performance, facilitating a greater comprehension of its advantages and disadvantages.

After a meticulous examination of the simulation outcomes and the rankings depicted in the tables, several deductions emerge:

- Notably, our model estimates demonstrate consistency for both SRS and RSS data sets. This consistency implies that the estimates progressively approach to the true parameter values as the sample size expands.
- Every metric used shows a similar trend: a decline with increasing sample size. This trend implies that more accurate and precise parameter estimations are produced with larger sample numbers.
- Our simulation findings for the SRS and RSS data sets suggest that MXPSE is the best technique when assessing the precision of our calculations.
- Estimates from RSS data sets show more efficiency than estimates from SRS data sets, as seen in Suppl Table 11. This result suggests that RSS is a more effective sampling technique, producing estimates with a lower MSE.

## Real data analysis

This section emphasizes the usefulness of the suggested estimation techniques by thoroughly elaborating on a real data set. This analysis clarifies how these estimation methods may be applied to real data, demonstrating their usefulness and applicability in real-world research and decision-making scenarios. The data set under consideration features the total milk production during the initial birth of 107 cows from the SINDI. This data set was investigated by Abd El-Bar et al.^12^, and its values are as follows: 0.4365, 0.4260, 0.5140, 0.6907, 0.7471, 0.2605, 0.6196, 0.8781, 0.4990, 0.6058, 0.6891, 0.5770, 0.5394, 0.1479, 0.2356, 0.6012, 0.1525, 0.5483, 0.6927, 0.7261, 0.3323, 0.0671, 0.2361, 0.4800, 0.5707, 0.7131, 0.5853, 0.6768, 0.5350, 0.4151, 0.6789, 0.4576, 0.3259, 0.2303, 0.7687, 0.4371, 0.3383, 0.6114, 0.3480, 0.4564, 0.7804, 0.3406, 0.4823, 0.5912, 0.5744, 0.5481, 0.1131, 0.7290, 0.0168, 0.5529, 0.4530, 0.3891, 0.4752, 0.3134, 0.3175, 0.1167, 0.6750, 0.5113, 0.5447, 0.4143, 0.5627, 0.5150, 0.0776, 0.3945, 0.4553, 0.4470, 0.5285, 0.5232, 0.6465, 0.0650, 0.8492, 0.8147, 0.3627, 0.3906, 0.4438, 0.4612, 0.3188, 0.2160, 0.6707, 0.6220, 0.5629, 0.4675, 0.6844, 0.3413, 0.4332, 0.0854, 0.3821, 0.4694, 0.3635, 0.4111, 0.5349, 0.3751, 0.1546, 0.4517, 0.2681, 0.4049, 0.5553, 0.5878, 0.4741, 0.3598, 0.7629, 0.5941, 0.6174, 0.6860, 0.0609, 0.6488, 0.2747.

**Figure 2:**
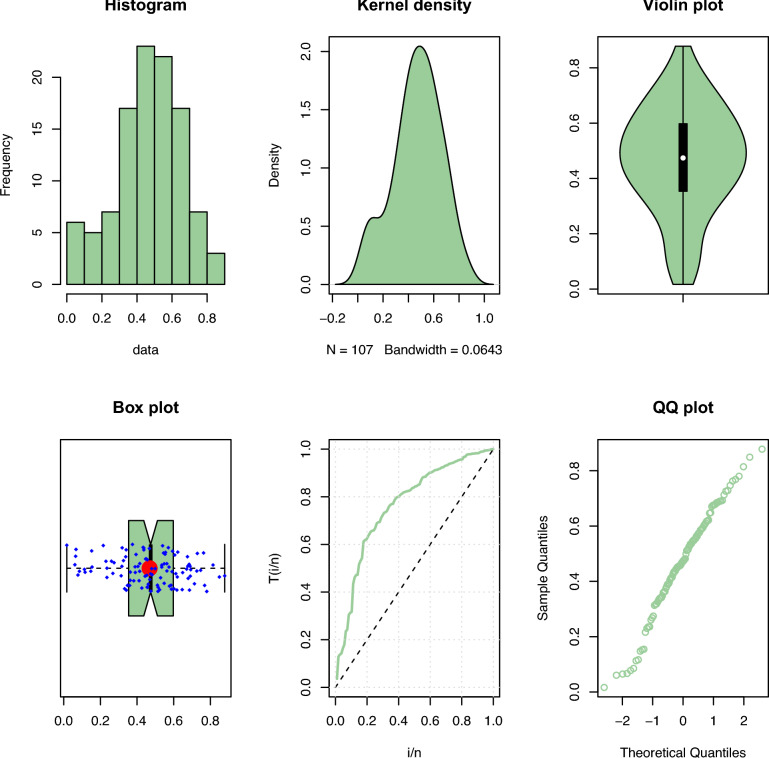
Some plots for the real data set.

**Figure 3:**
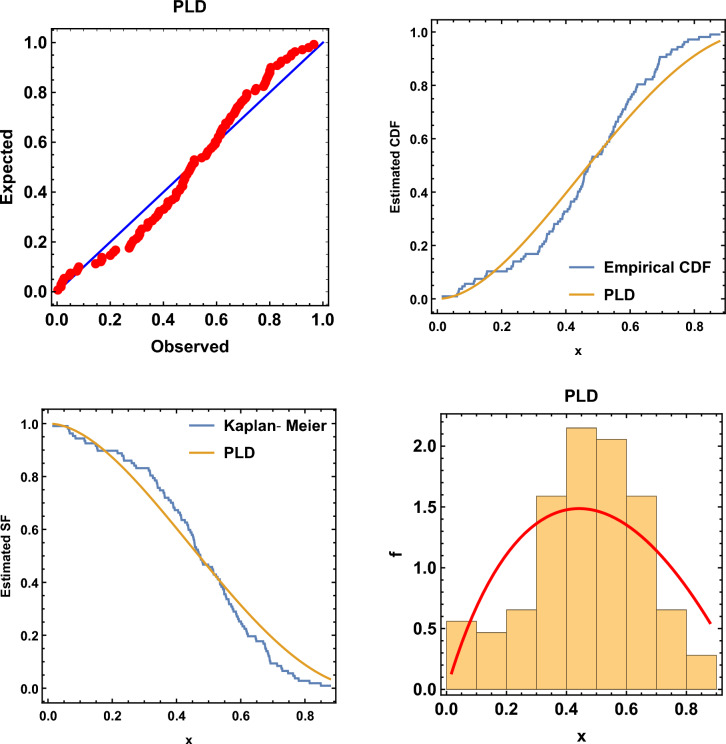
P–P plot, estimated CDF, estimated survival function, and histogram for the PLD.

**Figure 4:**
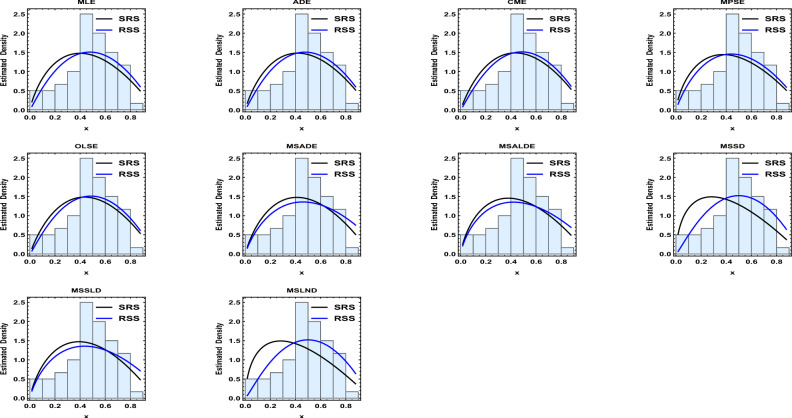
Plots of the estimated PDFs of the PLD with histogram for the two sampling methods when $s^ \cdot =60$.

**Figure 5:**
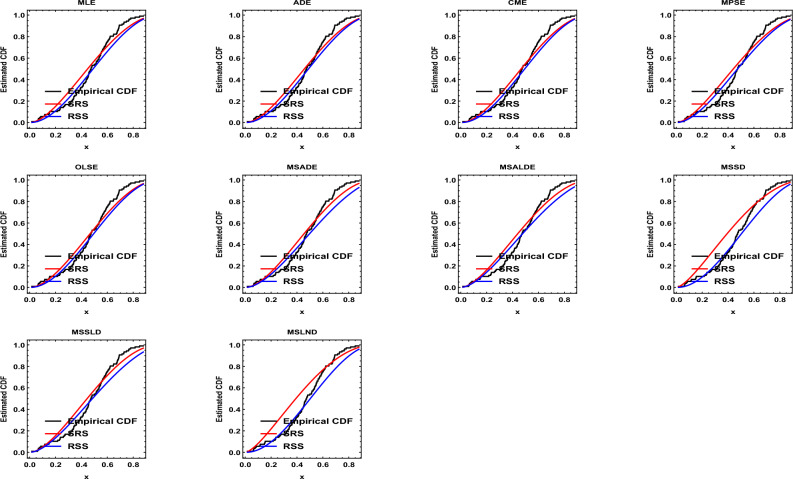
Plots of the estimated CDFs of the PLD for the SRS and RSS for the two sampling methods when $s^ \cdot =60$.

Results of the descriptive analysis can be found in Table 1. A variety of graphical representations is shown in Fig. 2, including histograms, quantile-quantile (Q-Q) plots, violin plots, box plots, total time on test (TTT) plots, and kernel density plots. The probability-probability (P-P) plot, estimated CDF, estimated survival function, and a histogram with the estimated PDF, are given in Fig. 3. The SRS and RSS estimates obtained from the PLD are shown in Tables 2 and 3, respectively. Several goodness-of-fit statistics, namely from the Anderson–Darling test (AT), the Cramer–von-Mises test (WT), and the Kolmogorov–Smirnov test (KST), are used to assess the models, see Table 4. These values (the smaller, the better) demonstrate that RSS is better than SRS for various estimation techniques. Moreover, it is evident from Figs. 4 and 5 how well the models fit the data.

**Table 1:** Descriptive statistics.

$s^ \cdot$	Mean	Median	Skewness	Kurtosis	Range	Minimum	Maximum	Sum
107	0.0168	0.4741	$-0.3306$	$-0.3638$	0.8613	0.0168	0.8781	50.1671

**Table 2:** Values for various estimators based on SRS for different values of $s^ \cdot$.

$s^ \cdot$	Est.	ML	AD	CRM	MXPS	LS	SPAD	SPALoD	MSSD	MSSLD	MSLND
20	$\hat{a}$	1.43048	1.40753	1.43026	1.30113	1.424	0.904541	1.30113	0.0964535	1.30843	0.104748
	$\hat{b}$	$1.1654\times 10^{-9}$	0.394726	$1.08697\times 10^-7$	874.363	0.40385	0.151353	16.6021	14747.9	2.12035	3.88514
	$\hat{c}$	1.20641	429831.	0.771405	90497.5	$2.85\times 10^8$	2.29242	1718.37	0.590897	$1.36352\times 10^6$	$5.91478\times 10^{-7}$
40	$\hat{a}$	1.31084	1.24601	1.25287	1.25454	1.25012	1.19105	1.25456	1.07241	1.36415	1.07274
	$\hat{b}$	3.0246	2.3941	0.603904	0.157244	1.10931	0.100354	4.05452	2.98036	0.360713	$1.75939\times 10^{-7}$
	$\hat{c}$	$5.70623\times 10^7$	$4.91932\times 10^6$	$2.3094\times 10^7$	16124.2	506978.	7565.71	$2.36535\times 10^6$	146431	$1.09889\times 10^6$	1.11529
60	$\hat{a}$	1.08963	1.14461	1.20208	0.999184	1.20225	1.12833	1.01889	0.793657	1.06579	0.788221
	$\hat{b}$	0.100069	0.04876	2.21269	3942.18	0.290438	0.100006	153509.	0.405077	0.12035	$7.47944\times 10^{-8}$
	$\hat{c}$	$2.08944\times 10^6$	325788.	$1.69354\times 10^7$	183870	$2.58634\times 10^7$	1673.94	$1.39044\times 10^7$	$1.34382\times 10^6$	86613.8	1.57541
80	$\hat{a}$	1.10546	0.841786	1.19126	1.06851	1.19085	1.25216	1.03718	1.58182	0.967496	1.58736
	b	12675.6	203042.	5.74555	0.854431	8.10385	0.105775	260980.	9.00435	10.6876	18.8045
	$\hat{c}$	$2.17605\times 10^{13}$	$1.31206\times 10^{6}$	$1.05383\times 10^{8}$	750809	958042.	14745.1	$1.71404\times 10^{7}$	63885.3	$1.75025\times 10^{7}$	237949
100	$\hat{a}$	1.1924	0.625184	1.26893	1.15834	1.26852	1.23451	1.16938	1.51738	1.06558	1.50024
	$\hat{b}$	29.1788	16059.3	10.0274	135.732	15.1774	79027.5	361.761	33.0389	3.50684	224.215
	$\hat{c}$	$2.30892\times 10^6$	34917.3	454385.	27468.8	$2.16066\times 10^7$	$1.57814\times 10^6$	$1.09782\times 10^7$	$1.30206\times 10^6$	942615	68799

**Table 3:** Values for various estimators based on RSS for different values of $s^ \cdot$.

$s^ \cdot$	Est.	ML	AD	CRME	MXPS	LS	SPAD	SPALoD	MSSD	MSSLD	MSLND
20	$\hat{a}$	1.2925	1.26419	1.26081	1.30301	1.25372	1.73876	1.39382	1.38601	1.27469	1.37028
	$\hat{b}$	10.2454	5.01094	159.927	2993.	7.79142	0.202211	11.8287	26.2623	$1.78189\times 10^{-11}$	31.9842
	$\hat{c}$	$1.12491\times 10^8$	$6.23194\times 10^6$	$5.24807\times 10^8$	143179	$5.20844\times 10^7$	$6.30247\times 10^6$	$4.45607\times 10^6$	$4.79981\times 10^9$	0.488326	11204
40	$\hat{a}$	1.46389	1.45031	1.45664	1.45613	1.45443	0.850463	1.15967	1.311	1.34815	1.30923
	$\hat{b}$	24.5555	13.3833	17.8271	12.4957	20.3694	7082.3	22.2325	342.471	$1.02178\times 10^-9$	11.9198
	$\hat{c}$	$1.11217\times 10^9$	$3.92682\times 10^7$	329849	$1.27894\times 10^6$	$5.75129\times 10^7$	23558.6	$1.78613\times 10^7$	$1.91837\times 10^9$	1.54045	80517.9
60	$\hat{a}$	1.35325	1.34632	1.38406	1.19084	1.3831	1.12833	1.02833	1.44385	1.06983	1.4409
	$\hat{b}$	1.24509	0.010936	1.3848	1258.93	16.3061	0.100006	0.100001	$8.92162\times 10^{-9}$	0.100107	$1.16053\times 10^{-8}$
	$\hat{c}$	$1.25147\times 10^8$	384756.	$1.34145\times 10^6$	63057.2	$3.3309\times 10^7$	0.937451	0.964921	0.459674	0.979211	0.353558
80	$\hat{a}$	1.18551	1.1801	1.20608	1.13977	1.20551	1.25214	0.999928	1.31201	0.978852	0.201663
	$\hat{b}$	15.8075	15.0351	21.3578	8.12685	2.67734	12.3202	8.96797	7.56202	$3.65314\times 10^{-12}$	$3.93703\times 10^6$
	$\hat{c}$	$3.85208\times 10^9$	$6.8484\times 10^7$	$1.90074\times 10^6$	691676	170598	342311	4842.94	705700	0.904663	$2.57847\times 10^6$
100	$\hat{a}$	1.23088	1.22766	1.30393	1.07103	1.30323	0.236196	0.0571355	1.79332	0.754801	1.79397
	$\hat{b}$	$3.17858\times 10^{-10}$	$2.89103\times 10^{-15}$	$3.58314\times 10^{-17}$	$5.61519\times 10^{-9}$	13.5738	13.3967	3705.6	$7.67335\times 10^{-10}$	0.164378	$1.13057\times 10^{-9}$
	$\hat{c}$	$1.32351\times 10^6$	3.9386	0.581916	0.428129	530667.	2.01181	232.269	0.520082	1.3064	0.0997822

**Table 4:** Parameter estimates and goodness-of-fit measures for the SRS and RSS designs with $s^ \cdot =60$.

Method	Design	$\hat{a}$	$\hat{b}$	$\hat{c}$	AT	WT	KST
ML	SRS	1.08963	0.100069	$2.08944\times 10^6$	2.17557	0.358186	0.150029
	RSS	1.35325	1.24509	$1.25147\times 10^8$	1.22693	0.17796	0.11505
AD	SRS	1.14461	0.04876	325788	2.1395	0.333795	0.157366
	RSS	1.34632	0.010936	384756	1.22646	0.178962	0.116644
CRM	SRS	1.20208	2.21269	$1.69354\times 10^7$	2.1774	0.325384	0.165076
	RSS	1.38406	1.3848	$1.34145\times 10^6$	1.24028	0.175973	0.11425
MXPS	SRS	0.999184	3942.18	183870	2.39583	0.413046	0.152589
	RSS	1.19084	1258.93	63057.2	1.47821	0.240442	0.142101
LS	SRS	1.20225	0.290438	$2.58634\times 10^7$	2.17762	0.325384	0.165099
	RSS	1.3831	16.3061	$3.3309\times 10^7$	1.23958	0.175974	0.114165
SPAD	SRS	1.12833	0.100006	1673.94	2.14302	0.339339	0.155235
	RSS	1.12833	0.100006	0.937451	1.98519	0.305023	0.151386
SPALoD	SRS	1.01889	153509	$1.39044\times 10^7$	2.33543	0.403704	0.152126
	RSS	1.02833	0.100001	0.964921	1.97596	0.317579	0.14366
MSSD	SRS	0.793657	0.405077	$1.34382\times 10^6$	3.78251	0.802906	0.221374
	RSS	1.44385	$8.92162\times 10^{-9}$	0.459674	1.3169	0.183221	0.119541
MSSLD	SRS	1.06579	0.12035	86613.8	2.21424	0.37395	0.146863
	RSS	1.06983	0.100107	0.979211	1.93282	0.303017	0.14248
MSLND	SRS	0.788221	$7.47944\times 10^{-8}$	1.57541	3.83752	0.816582	0.223016
	RSS	1.4409	$1.16053\times 10^{-8}$	0.353558	1.31159	0.18253	0.119279

## Concluding remarks

The accuracy of parameter estimators is considerably influenced by the sampling technique used in statistical parameter estimation problems. In the current work, the parameter estimates of the PLD are examined using both SRS and RSS approaches. The various estimates obtained by RSS were contrasted with those obtained through SRS. Six metrics were used to evaluate the effectiveness of the estimation methods. Based on our simulation findings for the SRS and RSS data sets, the MXPS method seems to be the best choice in terms of accuracy of the estimates. For both the SRS and RSS data sets, our model estimates show consistency. It may be inferred from this consistency that as the sample size increases, the estimates gradually become closer to the actual parameter values. Compared to estimates obtained from RSS data sets, those created from SRS data sets are less efficient.

### Supplementary Information

Supplementary Information.

## Data Availability

Any data that supports the findings of this study is included in the article.
