# Supplementary material for: Estimation methods based on ranked set sampling for the power logarithmic distribution
Source: Sci Rep. 2024 Jul 31;14:17652. doi: 10.1038/s41598-024-67693-4 (PMC11291687; doi:10.1038/s41598-024-67693-4)
Supplement: Supplementary file 1 — Supplementary Information. [file 41598_2024_67693_MOESM1_ESM.pdf]

Table 1: Numerical values for some measures for $a = 1.5,\ b = 0.75,\ c = 2.0$ under SRS.

$s^\bullet$	Measures	Est.	ML	AD	CRM	MXPS	LS	SPAD	SPALoD	MSSD	MSSLD	MSLND
30	BIAS	$\hat{a}$	$0.39778^{\{4\}}$	$0.41425^{\{8\}}$	$0.44189^{\{10\}}$	$0.35191^{\{1\}}$	$0.40494^{\{6\}}$	$0.39874^{\{5\}}$	$0.39041^{\{3\}}$	$0.41319^{\{7\}}$	$0.38283^{\{2\}}$	$0.41959^{\{9\}}$
		$\hat{b}$	$0.36944^{\{5\}}$	$0.38828^{\{9\}}$	$0.39445^{\{10\}}$	$0.34852^{\{1\}}$	$0.37608^{\{7\}}$	$0.3676^{\{2\}}$	$0.36855^{\{3\}}$	$0.38159^{\{8\}}$	$0.36917^{\{4\}}$	$0.37258^{\{6\}}$
		$\hat{c}$	$0.30685^{\{2\}}$	$0.36623^{\{6\}}$	$0.38484^{\{10\}}$	$0.30494^{\{1\}}$	$0.36904^{\{8\}}$	$0.36727^{\{7\}}$	$0.34794^{\{5\}}$	$0.34714^{\{4\}}$	$0.30885^{\{3\}}$	$0.37262^{\{9\}}$
	MSE	$\hat{a}$	$0.25849^{\{5\}}$	$0.27347^{\{7\}}$	$0.30289^{\{10\}}$	$0.19926^{\{1\}}$	$0.2672^{\{6\}}$	$0.25403^{\{4\}}$	$0.24261^{\{3\}}$	$0.27448^{\{8\}}$	$0.23603^{\{2\}}$	$0.27581^{\{9\}}$
		$\hat{b}$	$0.17859^{\{4\}}$	$0.19402^{\{9\}}$	$0.19906^{\{10\}}$	$0.15988^{\{1\}}$	$0.18634^{\{7\}}$	$0.1796^{\{5\}}$	$0.17636^{\{2\}}$	$0.18764^{\{8\}}$	$0.17853^{\{3\}}$	$0.18278^{\{6\}}$
		$\hat{c}$	$0.14655^{\{3\}}$	$0.21847^{\{7\}}$	$0.24543^{\{10\}}$	$0.1427^{\{1\}}$	$0.22364^{\{9\}}$	$0.22281^{\{8\}}$	$0.19301^{\{5\}}$	$0.18328^{\{4\}}$	$0.14627^{\{2\}}$	$0.21103^{\{6\}}$
	MRE	$\hat{a}$	$0.26519^{\{4\}}$	$0.27616^{\{8\}}$	$0.2946^{\{10\}}$	$0.23461^{\{1\}}$	$0.26996^{\{6\}}$	$0.26583^{\{5\}}$	$0.26028^{\{3\}}$	$0.27546^{\{7\}}$	$0.25522^{\{2\}}$	$0.27973^{\{9\}}$
		$\hat{b}$	$0.49259^{\{5\}}$	$0.51771^{\{9\}}$	$0.52594^{\{10\}}$	$0.46469^{\{1\}}$	$0.50144^{\{7\}}$	$0.49014^{\{2\}}$	$0.4914^{\{3\}}$	$0.50879^{\{8\}}$	$0.49223^{\{4\}}$	$0.49677^{\{6\}}$
		$\hat{c}$	$0.15343^{\{2\}}$	$0.18311^{\{6\}}$	$0.19242^{\{10\}}$	$0.15247^{\{1\}}$	$0.18452^{\{8\}}$	$0.18364^{\{7\}}$	$0.17397^{\{5\}}$	$0.17357^{\{4\}}$	$0.15443^{\{3\}}$	$0.18631^{\{9\}}$
	D_{abs}		$0.0428^{\{1\}}$	$0.0434^{\{3\}}$	$0.04474^{\{4\}}$	$0.04309^{\{2\}}$	$0.04487^{\{5\}}$	$0.05229^{\{8\}}$	$0.04918^{\{7\}}$	$0.05285^{\{9\}}$	$0.0458^{\{6\}}$	$0.05488^{\{10\}}$
	$D_{\max}$		$0.06805^{\{2\}}$	$0.06916^{\{3\}}$	$0.07217^{\{6\}}$	$0.06524^{\{1\}}$	$0.07045^{\{5\}}$	$0.07932^{\{8\}}$	$0.07453^{\{7\}}$	$0.07935^{\{9\}}$	$0.07031^{\{4\}}$	$0.08245^{\{10\}}$
	ASAE		$0.0356^{\{5\}}$	$0.03191^{\{2\}}$	$0.03201^{\{3\}}$	$0.03395^{\{4\}}$	$0.03148^{\{1\}}$	$0.04799^{\{9\}}$	$0.04376^{\{7\}}$	$0.04704^{\{8\}}$	$0.03927^{\{6\}}$	$0.04823^{\{10\}}$
	$\sum$ Ranks		$42^{\{3\}}$	$77^{\{7\}}$	$103^{\{10\}}$	$16^{\{1\}}$	$75^{\{6\}}$	$70^{\{5\}}$	$53^{\{4\}}$	$84^{\{8\}}$	$41^{\{2\}}$	$99^{\{9\}}$
75	BIAS	$\hat{a}$	$0.28967^{\{5\}}$	$0.28282^{\{4\}}$	$0.32708^{\{9\}}$	$0.25666^{\{1\}}$	$0.30007^{\{6\}}$	$0.30918^{\{7\}}$	$0.27624^{\{2\}}$	$0.32386^{\{8\}}$	$0.2808^{\{3\}}$	$0.3311^{\{10\}}$
		$\hat{b}$	$0.32826^{\{5\}}$	$0.31433^{\{2\}}$	$0.33928^{\{9\}}$	$0.30382^{\{1\}}$	$0.33325^{\{7\}}$	$0.33372^{\{8\}}$	$0.3213^{\{3\}}$	$0.33126^{\{6\}}$	$0.32272^{\{4\}}$	$0.33971^{\{10\}}$
		$\hat{c}$	$0.24523^{\{2\}}$	$0.26154^{\{4\}}$	$0.3021^{\{10\}}$	$0.23965^{\{1\}}$	$0.29721^{\{9\}}$	$0.2926^{\{8\}}$	$0.27971^{\{6\}}$	$0.2718^{\{5\}}$	$0.24538^{\{3\}}$	$0.29077^{\{7\}}$
	MSE	$\hat{a}$	$0.1392^{\{5\}}$	$0.13305^{\{4\}}$	$0.17708^{\{9\}}$	$0.10764^{\{1\}}$	$0.15124^{\{6\}}$	$0.16016^{\{7\}}$	$0.12478^{\{2\}}$	$0.17377^{\{8\}}$	$0.13107^{\{3\}}$	$0.18549^{\{10\}}$
		$\hat{b}$	$0.14549^{\{5\}}$	$0.13616^{\{2\}}$	$0.15401^{\{9\}}$	$0.12903^{\{1\}}$	$0.14946^{\{6\}}$	$0.15289^{\{8\}}$	$0.14102^{\{3\}}$	$0.1502^{\{7\}}$	$0.14371^{\{4\}}$	$0.15507^{\{10\}}$
		$\hat{c}$	$0.09897^{\{3\}}$	$0.12848^{\{5\}}$	$0.16135^{\{10\}}$	$0.0928^{\{1\}}$	$0.1512^{\{9\}}$	$0.14101^{\{8\}}$	$0.13571^{\{7\}}$	$0.12411^{\{4\}}$	$0.09563^{\{2\}}$	$0.1351^{\{6\}}$
	MRE	$\hat{a}$	$0.19311^{\{5\}}$	$0.18855^{\{4\}}$	$0.21805^{\{9\}}$	$0.1711^{\{1\}}$	$0.20005^{\{6\}}$	$0.20612^{\{7\}}$	$0.18416^{\{2\}}$	$0.2159^{\{8\}}$	$0.1872^{\{3\}}$	$0.22073^{\{10\}}$
		$\hat{b}$	$0.43767^{\{5\}}$	$0.41911^{\{2\}}$	$0.45237^{\{9\}}$	$0.4051^{\{1\}}$	$0.44434^{\{7\}}$	$0.44495^{\{8\}}$	$0.4284^{\{3\}}$	$0.44168^{\{6\}}$	$0.4303^{\{4\}}$	$0.45295^{\{10\}}$
		$\hat{c}$	$0.12262^{\{2\}}$	$0.13077^{\{4\}}$	$0.15105^{\{10\}}$	$0.11982^{\{1\}}$	$0.1486^{\{9\}}$	$0.1463^{\{8\}}$	$0.13985^{\{6\}}$	$0.1359^{\{5\}}$	$0.12269^{\{3\}}$	$0.14539^{\{7\}}$
	D_{abs}		$0.02825^{\{3\}}$	$0.02796^{\{2\}}$	$0.03017^{\{5\}}$	$0.0275^{\{1\}}$	$0.02912^{\{4\}}$	$0.03503^{\{8\}}$	$0.03159^{\{7\}}$	$0.036^{\{9\}}$	$0.03053^{\{6\}}$	$0.03632^{\{10\}}$
	$D_{\max}$		$0.04617^{\{3\}}$	$0.04525^{\{2\}}$	$0.04971^{\{7\}}$	$0.04323^{\{1\}}$	$0.04728^{\{4\}}$	$0.05495^{\{8\}}$	$0.0495^{\{6\}}$	$0.05611^{\{9\}}$	$0.04832^{\{5\}}$	$0.05689^{\{10\}}$
	ASAE		$0.0207^{\{5\}}$	$0.01881^{\{1\}}$	$0.01913^{\{3\}}$	$0.02031^{\{4\}}$	$0.01886^{\{2\}}$	$0.02933^{\{8\}}$	$0.02633^{\{7\}}$	$0.03017^{\{9\}}$	$0.02428^{\{6\}}$	$0.03046^{\{10\}}$
	$\sum$ Ranks		$48^{\{4\}}$	$36^{\{2\}}$	$99^{\{9\}}$	$15^{\{1\}}$	$75^{\{6\}}$	$93^{\{8\}}$	$54^{\{5\}}$	$84^{\{7\}}$	$46^{\{3\}}$	$110^{\{10\}}$
150	BIAS	$\hat{a}$	$0.21515^{\{4\}}$	$0.21168^{\{3\}}$	$0.22962^{\{6\}}$	$0.19639^{\{1\}}$	$0.23456^{\{7\}}$	$0.24599^{\{8\}}$	$0.22519^{\{5\}}$	$0.26124^{\{10\}}$	$0.21139^{\{2\}}$	$0.25385^{\{9\}}$
		$\hat{b}$	$0.28004^{\{5\}}$	$0.27592^{\{2\}}$	$0.27963^{\{4\}}$	$0.27356^{\{1\}}$	$0.27797^{\{3\}}$	$0.30235^{\{9\}}$	$0.28384^{\{7\}}$	$0.30749^{\{10\}}$	$0.28207^{\{6\}}$	$0.29945^{\{8\}}$
		$\hat{c}$	$0.21625^{\{3\}}$	$0.22719^{\{4\}}$	$0.24574^{\{9\}}$	$0.20994^{\{1\}}$	$0.23291^{\{6\}}$	$0.25751^{\{10\}}$	$0.22723^{\{5\}}$	$0.24213^{\{8\}}$	$0.21428^{\{2\}}$	$0.23696^{\{7\}}$
	MSE	$\hat{a}$	$0.07481^{\{4\}}$	$0.07191^{\{3\}}$	$0.08887^{\{7\}}$	$0.06078^{\{1\}}$	$0.08588^{\{6\}}$	$0.09547^{\{8\}}$	$0.07904^{\{5\}}$	$0.10754^{\{10\}}$	$0.07007^{\{2\}}$	$0.10242^{\{9\}}$
		$\hat{b}$	$0.11032^{\{1\}}$	$0.11112^{\{3\}}$	$0.1121^{\{4\}}$	$0.11043^{\{2\}}$	$0.11257^{\{5\}}$	$0.1273^{\{9\}}$	$0.11355^{\{7\}}$	$0.13203^{\{10\}}$	$0.11294^{\{6\}}$	$0.12681^{\{8\}}$
		$\hat{c}$	$0.08028^{\{3\}}$	$0.09349^{\{6\}}$	$0.11281^{\{9\}}$	$0.07554^{\{1\}}$	$0.1024^{\{8\}}$	$0.11802^{\{10\}}$	$0.08694^{\{4\}}$	$0.09961^{\{7\}}$	$0.07661^{\{2\}}$	$0.08856^{\{5\}}$
	MRE	$\hat{a}$	$0.14344^{\{4\}}$	$0.14112^{\{3\}}$	$0.15308^{\{6\}}$	$0.13092^{\{1\}}$	$0.15637^{\{7\}}$	$0.16399^{\{8\}}$	$0.15013^{\{5\}}$	$0.17416^{\{10\}}$	$0.14093^{\{2\}}$	$0.16923^{\{9\}}$
		$\hat{b}$	$0.37339^{\{5\}}$	$0.36789^{\{2\}}$	$0.37284^{\{4\}}$	$0.36475^{\{1\}}$	$0.37063^{\{3\}}$	$0.40314^{\{9\}}$	$0.37846^{\{7\}}$	$0.40998^{\{10\}}$	$0.37609^{\{6\}}$	$0.39927^{\{8\}}$
		$\hat{c}$	$0.10812^{\{3\}}$	$0.11359^{\{4\}}$	$0.12287^{\{9\}}$	$0.10497^{\{1\}}$	$0.11646^{\{6\}}$	$0.12875^{\{10\}}$	$0.11362^{\{5\}}$	$0.12106^{\{8\}}$	$0.10714^{\{2\}}$	$0.11848^{\{7\}}$
	D_{abs}		$0.01962^{\{2\}}$	$0.02066^{\{3\}}$	$0.02122^{\{4\}}$	$0.01928^{\{1\}}$	$0.02135^{\{5\}}$	$0.02537^{\{8\}}$	$0.02217^{\{7\}}$	$0.02555^{\{9\}}$	$0.02157^{\{6\}}$	$0.02673^{\{10\}}$
	$D_{\max}$		$0.03227^{\{2\}}$	$0.03351^{\{3\}}$	$0.03489^{\{6\}}$	$0.03085^{\{1\}}$	$0.03481^{\{5\}}$	$0.04053^{\{8\}}$	$0.03563^{\{7\}}$	$0.04087^{\{9\}}$	$0.03464^{\{4\}}$	$0.04231^{\{10\}}$
	ASAE		$0.01407^{\{4\}}$	$0.01314^{\{2\}}$	$0.01319^{\{3\}}$	$0.01417^{\{5\}}$	$0.01288^{\{1\}}$	$0.02019^{\{8\}}$	$0.01846^{\{7\}}$	$0.0213^{\{9\}}$	$0.01678^{\{6\}}$	$0.02196^{\{10\}}$
	$\sum$ Ranks		$40^{\{3\}}$	$38^{\{2\}}$	$71^{\{6.5\}}$	$17^{\{1\}}$	$62^{\{5\}}$	$105^{\{9\}}$	$71^{\{6.5\}}$	$110^{\{10\}}$	$46^{\{4\}}$	$100^{\{8\}}$
250	BIAS	ˆ	$0.17377^{\{2\}}$	$0.18552^{\{5\}}$	$0.19089^{\{7\}}$	$0.16705^{\{1\}}$	$0.19046^{\{6\}}$	$0.19524^{\{8\}}$	$0.18285^{\{3\}}$	$0.21524^{\{10\}}$	$0.18306^{\{4\}}$	$0.21345^{\{9\}}$
		$\hat{b}$	$0.24892^{\{4\}}$	$0.24971^{\{5\}}$	$0.24653^{\{2\}}$	$0.24422^{\{1\}}$	$0.24745^{\{3\}}$	$0.27167^{\{9\}}$	$0.2597^{\{7\}}$	$0.28753^{\{10\}}$	$0.25801^{\{6\}}$	$0.26964^{\{8\}}$
		$\hat{c}$	$0.17718^{\{1\}}$	$0.20698^{\{6\}}$	$0.20664^{\{5\}}$	$0.18019^{\{2\}}$	$0.20966^{\{7\}}$	$0.22429^{\{10\}}$	$0.20307^{\{4\}}$	$0.21139^{\{9\}}$	$0.19352^{\{3\}}$	$0.20982^{\{8\}}$
	MSE	$\hat{a}$	$0.04655^{\{2\}}$	$0.05189^{\{3\}}$	$0.05746^{\{7\}}$	$0.0435^{\{1\}}$	$0.05715^{\{6\}}$	$0.06063^{\{8\}}$	$0.05261^{\{5\}}$	$0.06952^{\{9\}}$	$0.05192^{\{4\}}$	$0.07333^{\{10\}}$
		$\hat{b}$	$0.09149^{\{3\}}$	$0.09295^{\{5\}}$	$0.09058^{\{2\}}$	$0.08996^{\{1\}}$	$0.09236^{\{4\}}$	$0.10717^{\{9\}}$	$0.0993^{\{7\}}$	$0.1175^{\{10\}}$	$0.09857^{\{6\}}$	$0.10681^{\{8\}}$
		$\hat{c}$	$0.05506^{\{1\}}$	$0.08313^{\{9\}}$	$0.08034^{\{7\}}$	$0.05817^{\{2\}}$	$0.08054^{\{8\}}$	$0.09144^{\{10\}}$	$0.07178^{\{4\}}$	$0.07299^{\{5\}}$	$0.06688^{\{3\}}$	$0.07465^{\{6\}}$
	MRE	$\hat{a}$	$0.11585^{\{2\}}$	$0.12368^{\{5\}}$	$0.12726^{\{7\}}$	$0.11137^{\{1\}}$	$0.12697^{\{6\}}$	$0.13016^{\{8\}}$	$0.1219^{\{3\}}$	$0.14349^{\{10\}}$	$0.12204^{\{4\}}$	$0.1423^{\{9\}}$
		$\hat{b}$	$0.33189^{\{4\}}$	$0.33295^{\{5\}}$	$0.3287^{\{2\}}$	$0.32563^{\{1\}}$	$0.32994^{\{3\}}$	$0.36223^{\{9\}}$	$0.34627^{\{7\}}$	$0.38338^{\{10\}}$	$0.34402^{\{6\}}$	$0.35952^{\{8\}}$
		$\hat{c}$	$0.08859^{\{1\}}$	$0.10349^{\{6\}}$	$0.10332^{\{5\}}$	$0.0901^{\{2\}}$	$0.10483^{\{7\}}$	$0.11215^{\{10\}}$	$0.10153^{\{4\}}$	$0.1057^{\{9\}}$	$0.09676^{\{3\}}$	$0.10491^{\{8\}}$
	D_{abs}		$0.01571^{\{2\}}$	$0.01575^{\{3\}}$	$0.01643^{\{5\}}$	$0.0156^{\{1\}}$	$0.01635^{\{4\}}$	$0.02008^{\{8\}}$	$0.01792^{\{7\}}$	$0.02085^{\{9.5\}}$	$0.0171^{\{6\}}$	$0.02085^{\{9.5\}}$
	$D_{\max}$		$0.02575^{\{2\}}$	$0.02608^{\{3\}}$	$0.02708^{\{5\}}$	$0.02523^{\{1\}}$	$0.02692^{\{4\}}$	$0.03226^{\{8\}}$	$0.02899^{\{7\}}$	$0.03355^{\{10\}}$	$0.02777^{\{6\}}$	$0.03348^{\{9\}}$
	ASAE		$0.0106^{\{5\}}$	$0.01001^{\{3\}}$	$0.00994^{\{2\}}$	$0.01058^{\{4\}}$	$0.00989^{\{1\}}$	$0.01567^{\{8\}}$	$0.01409^{\{7\}}$	$0.01693^{\{9\}}$	$0.01279^{\{6\}}$	$0.01715^{\{10\}}$
	$\sum$ Ranks		$29^{\{2\}}$	$58^{\{5\}}$	$56^{\{3\}}$	$18^{\{1\}}$	$59^{\{6\}}$	$105^{\{9\}}$	$65^{\{7\}}$	$110.5^{\{10\}}$	$57^{\{4\}}$	$102.5^{\{8\}}$
400	BIAS	$\hat{a}$	$0.13402^{\{1\}}$	$0.14575^{\{4\}}$	$0.16341^{\{7\}}$	$0.13785^{\{2\}}$	$0.15372^{\{6\}}$	$0.16579^{\{8\}}$	$0.15277^{\{5\}}$	$0.18453^{\{9\}}$	$0.14464^{\{3\}}$	$0.18509^{\{10\}}$
		$\hat{b}$	$0.20487^{\{1\}}$	$0.21161^{\{2\}}$	$0.22242^{\{6\}}$	$0.21976^{\{3\}}$	$0.222^{\{5\}}$	$0.24548^{\{8\}}$	$0.23599^{\{7\}}$	$0.25714^{\{9\}}$	$0.22091^{\{4\}}$	$0.2646^{\{10\}}$
		$\hat{c}$	$0.14659^{\{1\}}$	$0.164^{\{3\}}$	$0.19796^{\{10\}}$	$0.1525^{\{2\}}$	$0.17524^{\{5\}}$	$0.18438^{\{6\}}$	$0.18457^{\{7\}}$	$0.19109^{\{8\}}$	$0.16962^{\{4\}}$	$0.19364^{\{9\}}$
	MSE	$\hat{a}$	$0.0275^{\{1\}}$	$0.03351^{\{4\}}$	$0.04187^{\{7\}}$	$0.02992^{\{2\}}$	$0.03705^{\{5\}}$	$0.04292^{\{8\}}$	$0.03755^{\{6\}}$	$0.05351^{\{10\}}$	$0.03205^{\{3\}}$	$0.05004^{\{9\}}$
		$\hat{b}$	$0.0655^{\{1\}}$	$0.07193^{\{2\}}$	$0.07626^{\{3\}}$	$0.07845^{\{5\}}$	$0.07865^{\{6\}}$	$0.09182^{\{8\}}$	$0.0871^{\{7\}}$	$0.09991^{\{9\}}$	$0.07695^{\{4\}}$	$0.10294^{\{10\}}$
		$\hat{c}$	$0.03753^{\{1\}}$	$0.04873^{\{3\}}$	$0.08002^{\{10\}}$	$0.04242^{\{2\}}$	$0.06048^{\{5\}}$	$0.06075^{\{6\}}$	$0.06245^{\{7\}}$	$0.06532^{\{8\}}$	$0.05041^{\{4\}}$	$0.06676^{\{9\}}$
	MRE	$\hat{a}$	$0.08935^{\{1\}}$	$0.09717^{\{4\}}$	$0.10894^{\{7\}}$	$0.0919^{\{2\}}$	$0.10248^{\{6\}}$	$0.11053^{\{8\}}$	$0.10185^{\{5\}}$	$0.12302^{\{9\}}$	$0.09643^{\{3\}}$	$0.1234^{\{10\}}$
		$\hat{b}$	$0.27316^{\{1\}}$	$0.28214^{\{2\}}$	$0.29656^{\{6\}}$	$0.29301^{\{3\}}$	$0.296^{\{5\}}$	$0.32731^{\{8\}}$	$0.31465^{\{7\}}$	$0.34285^{\{9\}}$	$0.29454^{\{4\}}$	$0.35279^{\{10\}}$
		$\hat{c}$	$0.07329^{\{1\}}$	$0.082^{\{3\}}$	$0.09898^{\{10\}}$	$0.07625^{\{2\}}$	$0.08762^{\{5\}}$	$0.09219^{\{6\}}$	$0.09228^{\{7\}}$	$0.09555^{\{8\}}$	$0.08481^{\{4\}}$	$0.09682^{\{9\}}$
	D_{abs}		$0.01249^{\{2\}}$	$0.01282^{\{3\}}$	$0.01309^{\{5\}}$	$0.01247^{\{1\}}$	$0.01296^{\{4\}}$	$0.01601^{\{8\}}$	$0.01489^{\{7\}}$	$0.01668^{\{10\}}$	$0.01393^{\{6\}}$	$0.01645^{\{9\}}$
	$D_{\max}$		$0.02035^{\{2\}}$	$0.0211^{\{3\}}$	$0.02174^{\{5\}}$	$0.02024^{\{1\}}$	$0.02146^{\{4\}}$	$0.02604^{\{8\}}$	$0.02409^{\{7\}}$	$0.02704^{\{10\}}$	$0.02248^{\{6\}}$	$0.02701^{\{9\}}$
	ASAE		$0.00841^{\{4.5\}}$	$0.00787^{\{3\}}$	$0.00784^{\{2\}}$	$0.00841^{\{4.5\}}$	$0.00776^{\{1\}}$	$0.01202^{\{8\}}$	$0.011^{\{7\}}$	$0.01341^{\{10\}}$	$0.01018^{\{6\}}$	$0.01325^{\{9\}}$
	$\sum$ Ranks		$17.5^{\{1\}}$	$36^{\{3\}}$	$78^{\{6\}}$	$29.5^{\{2\}}$	$57^{\{5\}}$	$90^{\{8\}}$	$79^{\{7\}}$	$109^{\{9\}}$	$51^{\{4\}}$	$113^{\{10\}}$

Table 2: Numerical values for some measures for $a = 1.5,\ b = 0.75,\ c = 2.0$ under RSS.

$s^{\bullet}$	Measure	Est.	ML	AD	CRM	MXPS	LS	SPAD	SPALoD	MSSD	MSSLD	MSLND
30	BIAS	$\hat{a}$	$0.33337^{\{4\}}$	$0.33778^{\{5\}}$	$0.39439^{\{10\}}$	$0.28152^{\{1\}}$	$0.34652^{\{6\}}$	$0.35433^{\{7\}}$	$0.32815^{\{3\}}$	$0.36562^{\{8\}}$	$0.32787^{\{2\}}$	$0.36564^{\{9\}}$
		$\hat{b}$	$0.35293^{\{5\}}$	$0.35569^{\{8\}}$	$0.37111^{\{10\}}$	$0.32466^{\{1\}}$	$0.34764^{\{4\}}$	$0.36542^{\{9\}}$	$0.33666^{\{2\}}$	$0.35467^{\{7\}}$	$0.35318^{\{6\}}$	$0.34548^{\{3\}}$
		$\hat{c}$	$0.29033^{\{4\}}$	$0.29367^{\{5\}}$	$0.31979^{\{9\}}$	$0.241^{\{1\}}$	$0.3011^{\{6\}}$	$0.33649^{\{10\}}$	$0.29024^{\{3\}}$	$0.31323^{\{8\}}$	$0.27467^{\{2\}}$	$0.30986^{\{7\}}$
	MSE	$\hat{a}$	$0.18139^{\{4\}}$	$0.18547^{\{5\}}$	$0.25664^{\{10\}}$	$0.13044^{\{1\}}$	$0.19938^{\{6\}}$	$0.2082^{\{7\}}$	$0.17142^{\{2\}}$	$0.21409^{\{8\}}$	$0.17634^{\{3\}}$	$0.21874^{\{9\}}$
		$\hat{b}$	$0.16456^{\{5\}}$	$0.16685^{\{7\}}$	$0.18254^{\{10\}}$	$0.14601^{\{1\}}$	$0.16257^{\{4\}}$	$0.17834^{\{9\}}$	$0.15394^{\{2\}}$	$0.1677^{\{8\}}$	$0.16638^{\{6\}}$	$0.15989^{\{3\}}$
		$\hat{c}$	$0.14627^{\{3\}}$	$0.16747^{\{8\}}$	$0.18921^{\{9\}}$	$0.09618^{\{1\}}$	$0.16612^{\{7\}}$	$0.19292^{\{10\}}$	$0.1531^{\{4\}}$	$0.16365^{\{6\}}$	$0.1273^{\{2\}}$	$0.15899^{\{5\}}$
	MRE	$\hat{a}$	$0.22225^{\{4\}}$	$0.22519^{\{5\}}$	$0.26292^{\{10\}}$	$0.18768^{\{1\}}$	$0.23101^{\{6\}}$	$0.23622^{\{7\}}$	$0.21876^{\{3\}}$	$0.24375^{\{8\}}$	$0.21858^{\{2\}}$	$0.24376^{\{9\}}$
		$\hat{b}$	$0.47057^{\{5\}}$	$0.47425^{\{8\}}$	$0.49482^{\{10\}}$	$0.43288^{\{1\}}$	$0.46352^{\{4\}}$	$0.48723^{\{9\}}$	$0.44888^{\{2\}}$	$0.4729^{\{7\}}$	$0.47091^{\{6\}}$	$0.46063^{\{3\}}$
		$\hat{c}$	$0.14516^{\{4\}}$	$0.14683^{\{5\}}$	$0.1599^{\{9\}}$	$0.1205^{\{1\}}$	$0.15055^{\{6\}}$	$0.16825^{\{10\}}$	$0.14512^{\{3\}}$	$0.15661^{\{8\}}$	$0.13734^{\{2\}}$	$0.15493^{\{7\}}$
	D_{abs}		$0.02832^{\{2\}}$	$0.0289^{\{3\}}$	$0.0297^{\{5\}}$	$0.02671^{\{1\}}$	$0.02916^{\{4\}}$	$0.04189^{\{9\}}$	$0.03526^{\{7\}}$	$0.04156^{\{8\}}$	$0.03391^{\{6\}}$	$0.04245^{\{10\}}$
	$D_{\max}$		$0.04677^{\{3\}}$	$0.04651^{\{2\}}$	$0.04884^{\{5\}}$	$0.042^{\{1\}}$	$0.04698^{\{4\}}$	$0.06465^{\{10\}}$	$0.05456^{\{7\}}$	$0.06318^{\{8\}}$	$0.05294^{\{6\}}$	$0.0641^{\{9\}}$
	ASAE		$0.03416^{\{5\}}$	$0.03135^{\{2\}}$	$0.03167^{\{3\}}$	$0.0333^{\{4\}}$	$0.03041^{\{1\}}$	$0.04766^{\{10\}}$	$0.04202^{\{7\}}$	$0.04653^{\{8\}}$	$0.03908^{\{6\}}$	$0.04732^{\{9\}}$
	$\sum$ Ranks		$48^{\{3\}}$	$63^{\{6\}}$	$100^{\{9\}}$	$15^{\{1\}}$	$58^{\{5\}}$	$107^{\{10\}}$	$45^{\{2\}}$	$92^{\{8\}}$	$49^{\{4\}}$	$83^{\{7\}}$
75	BIAS	$\hat{a}$	$0.23394^{\{2\}}$	$0.246^{\{5\}}$	$0.26949^{\{7\}}$	$0.21623^{\{1\}}$	$0.24146^{\{4\}}$	$0.27459^{\{8\}}$	$0.25744^{\{6\}}$	$0.30408^{\{10\}}$	$0.23814^{\{3\}}$	$0.28615^{\{9\}}$
		$\hat{b}$	$0.28863^{\{1\}}$	$0.29517^{\{3\}}$	$0.31175^{\{7\}}$	$0.29545^{\{4\}}$	$0.29057^{\{2\}}$	$0.31854^{\{8\}}$	$0.30855^{\{6\}}$	$0.32916^{\{10\}}$	$0.30387^{\{5\}}$	$0.3233^{\{9\}}$
		$\hat{c}$	$0.21172^{\{2\}}$	$0.24382^{\{5\}}$	$0.25137^{\{7\}}$	$0.20217^{\{1\}}$	$0.23131^{\{4\}}$	$0.27054^{\{10\}}$	$0.25664^{\{8\}}$	$0.25759^{\{9\}}$	$0.21813^{\{3\}}$	$0.24804^{\{6\}}$
	MSE	$\hat{a}$	$0.08818^{\{2\}}$	$0.09729^{\{5\}}$	$0.11918^{\{7\}}$	$0.07094^{\{1\}}$	$0.09476^{\{4\}}$	$0.12577^{\{8\}}$	$0.10151^{\{6\}}$	$0.15164^{\{10\}}$	$0.09309^{\{3\}}$	$0.13361^{\{9\}}$
		$\hat{b}$	$0.11307^{\{1\}}$	$0.12284^{\{3\}}$	$0.13562^{\{7\}}$	$0.12378^{\{4\}}$	$0.12198^{\{2\}}$	$0.14072^{\{8\}}$	$0.1334^{\{6\}}$	$0.1485^{\{10\}}$	$0.12971^{\{5\}}$	$0.14239^{\{9\}}$
		$\hat{c}$	$0.08634^{\{3\}}$	$0.1163^{\{8\}}$	$0.11917^{\{9\}}$	$0.07915^{\{1\}}$	$0.10786^{\{5\}}$	$0.13574^{\{10\}}$	$0.11279^{\{6\}}$	$0.11308^{\{7\}}$	$0.07961^{\{2\}}$	$0.10266^{\{4\}}$
	MRE	$\hat{a}$	$0.15596^{\{2\}}$	$0.164^{\{5\}}$	$0.17966^{\{7\}}$	$0.14416^{\{1\}}$	$0.16097^{\{4\}}$	$0.18306^{\{8\}}$	$0.17163^{\{6\}}$	$0.20272^{\{10\}}$	$0.15876^{\{3\}}$	$0.19077^{\{9\}}$
		$\hat{b}$	$0.38484^{\{1\}}$	$0.39356^{\{3\}}$	$0.41567^{\{7\}}$	$0.39394^{\{4\}}$	$0.38742^{\{2\}}$	$0.42472^{\{8\}}$	$0.4114^{\{6\}}$	$0.43887^{\{10\}}$	$0.40516^{\{5\}}$	$0.43106^{\{9\}}$
		$\hat{c}$	$0.10586^{\{2\}}$	$0.12191^{\{5\}}$	$0.12569^{\{7\}}$	$0.10109^{\{1\}}$	$0.11566^{\{4\}}$	$0.13527^{\{10\}}$	$0.12832^{\{8\}}$	$0.1288^{\{9\}}$	$0.10907^{\{3\}}$	$0.12402^{\{6\}}$
	D_{abs}		$0.0182^{\{2\}}$	$0.01844^{\{4\}}$	$0.01988^{\{5\}}$	$0.01784^{\{1\}}$	$0.0184^{\{3\}}$	$0.02751^{\{8\}}$	$0.02466^{\{7\}}$	$0.0286^{\{10\}}$	$0.02141^{\{6\}}$	$0.02771^{\{9\}}$
	$D_{\max}$		$0.03024^{\{2\}}$	$0.0306^{\{4\}}$	$0.03329^{\{5\}}$	$0.02893^{\{1\}}$	$0.03047^{\{3\}}$	$0.04369^{\{8\}}$	$0.03934^{\{7\}}$	$0.04567^{\{10\}}$	$0.03461^{\{6\}}$	$0.0441^{\{9\}}$
	ASAE		$0.01991^{\{4\}}$	$0.01871^{\{2\}}$	$0.01878^{\{3\}}$	$0.02002^{\{5\}}$	$0.01848^{\{1\}}$	$0.02843^{\{8\}}$	$0.02592^{\{7\}}$	$0.02938^{\{10\}}$	$0.02335^{\{6\}}$	$0.02916^{\{9\}}$
	$\sum$ Ranks		$24^{\{1\}}$	$52^{\{5\}}$	$78^{\{6\}}$	$25^{\{2\}}$	$38^{\{3\}}$	$102^{\{9\}}$	$79^{\{7\}}$	$115^{\{10\}}$	$50^{\{4\}}$	$97^{\{8\}}$
150	BIAS	$\hat{a}$	$0.17391^{\{2\}}$	$0.18434^{\{3\}}$	$0.2133^{\{7\}}$	$0.1732^{\{1\}}$	$0.1914^{\{5\}}$	$0.22029^{\{8\}}$	$0.20182^{\{6\}}$	$0.23291^{\{10\}}$	$0.18897^{\{4\}}$	$0.23041^{\{9\}}$
		$\hat{b}$	$0.23568^{\{1\}}$	$0.24992^{\{2\}}$	$0.26705^{\{5\}}$	$0.258^{\{3\}}$	$0.26165^{\{4\}}$	$0.29215^{\{9\}}$	$0.27413^{\{7\}}$	$0.2914^{\{8\}}$	$0.26812^{\{6\}}$	$0.29228^{\{10\}}$
		$\hat{c}$	$0.17846^{\{1\}}$	$0.18724^{\{3\}}$	$0.22705^{\{9\}}$	$0.18053^{\{2\}}$	$0.21124^{\{5\}}$	$0.2279^{\{10\}}$	$0.21504^{\{6\}}$	$0.21985^{\{7\}}$	$0.19353^{\{4\}}$	$0.22276^{\{8\}}$
	MSE	$\hat{a}$	$0.04693^{\{1\}}$	$0.05255^{\{3\}}$	$0.07086^{\{7\}}$	$0.04729^{\{2\}}$	$0.05845^{\{5\}}$	$0.07588^{\{8\}}$	$0.06482^{\{6\}}$	$0.0861^{\{10\}}$	$0.05487^{\{4\}}$	$0.08382^{\{9\}}$
		$\hat{b}$	$0.08378^{\{1\}}$	$0.09426^{\{2\}}$	$0.10414^{\{5\}}$	$0.10006^{\{3\}}$	$0.10281^{\{4\}}$	$0.12079^{\{8\}}$	$0.11015^{\{7\}}$	$0.12102^{\{9\}}$	$0.10646^{\{6\}}$	$0.12257^{\{10\}}$
		$\hat{c}$	$0.05841^{\{1\}}$	$0.07058^{\{4\}}$	$0.10563^{\{10\}}$	$0.05965^{\{2\}}$	$0.0885^{\{8\}}$	$0.09559^{\{9\}}$	$0.08581^{\{7\}}$	$0.08117^{\{5\}}$	$0.07015^{\{3\}}$	$0.08273^{\{6\}}$
	MRE	$\hat{a}$	$0.11594^{\{2\}}$	$0.12289^{\{3\}}$	$0.1422^{\{7\}}$	$0.11547^{\{1\}}$	$0.1276^{\{5\}}$	$0.14686^{\{8\}}$	$0.13454^{\{6\}}$	$0.15528^{\{10\}}$	$0.12598^{\{4\}}$	$0.15361^{\{9\}}$
		$\hat{b}$	$0.31423^{\{1\}}$	$0.33322^{\{2\}}$	$0.35606^{\{5\}}$	$0.344^{\{3\}}$	$0.34887^{\{4\}}$	$0.38954^{\{9\}}$	$0.3655^{\{7\}}$	$0.38854^{\{8\}}$	$0.35749^{\{6\}}$	$0.38971^{\{10\}}$
		$\hat{c}$	$0.08923^{\{1\}}$	$0.09362^{\{3\}}$	$0.11353^{\{9\}}$	$0.09026^{\{2\}}$	$0.10562^{\{5\}}$	$0.11395^{\{10\}}$	$0.10752^{\{6\}}$	$0.10992^{\{7\}}$	$0.09677^{\{4\}}$	$0.11138^{\{8\}}$
	D_{abs}		$0.01313^{\{4\}}$	$0.0128^{\{2\}}$	$0.01371^{\{5\}}$	$0.01261^{\{1\}}$	$0.01293^{\{3\}}$	$0.01914^{\{8\}}$	$0.01775^{\{7\}}$	$0.02131^{\{10\}}$	$0.01558^{\{6\}}$	$0.02117^{\{9\}}$
	$D_{\max}$		$0.02192^{\{3\}}$	$0.02165^{\{2\}}$	$0.02342^{\{5\}}$	$0.02114^{\{1\}}$	$0.02195^{\{4\}}$	$0.03139^{\{8\}}$	$0.02898^{\{7\}}$	$0.03439^{\{10\}}$	$0.02561^{\{6\}}$	$0.03431^{\{9\}}$
	ASAE		$0.01364^{\{5\}}$	$0.01301^{\{2\}}$	$0.01313^{\{3\}}$	$0.01362^{\{4\}}$	$0.01285^{\{1\}}$	$0.01981^{\{8\}}$	$0.01832^{\{7\}}$	$0.02121^{\{9\}}$	$0.01635^{\{6\}}$	$0.02165^{\{10\}}$
	$\sum$ Ranks		$23^{\{1\}}$	$31^{\{3\}}$	$77^{\{6\}}$	$25^{\{2\}}$	$53^{\{4\}}$	$103^{\{8.5\}}$	$79^{\{7\}}$	$103^{\{8.5\}}$	$59^{\{5\}}$	$107^{\{10\}}$
250	BIAS	$\hat{a}$	$0.13588^{\{1\}}$	$0.14501^{\{2\}}$	$0.16836^{\{7\}}$	$0.15071^{\{3\}}$	$0.16776^{\{6\}}$	$0.18679^{\{8\}}$	$0.16567^{\{5\}}$	$0.19386^{\{10\}}$	$0.15852^{\{4\}}$	$0.18687^{\{9\}}$
		$\hat{b}$	$0.19918^{\{1\}}$	$0.21817^{\{2\}}$	$0.22819^{\{3\}}$	$0.2354^{\{6\}}$	$0.23439^{\{4\}}$	$0.25764^{\{9\}}$	$0.244^{\{7\}}$	$0.26768^{\{10\}}$	$0.235^{\{5\}}$	$0.25763^{\{8\}}$
		$\hat{c}$	$0.15158^{\{1\}}$	$0.1677^{\{3\}}$	$0.19006^{\{8\}}$	$0.15944^{\{2\}}$	$0.18793^{\{7\}}$	$0.19948^{\{9\}}$	$0.1822^{\{5\}}$	$0.20293^{\{10\}}$	$0.16812^{\{4\}}$	$0.18302^{\{6\}}$
	MSE	$\hat{a}$	$0.02841^{\{1\}}$	$0.03296^{\{2\}}$	$0.04329^{\{6\}}$	$0.03494^{\{3\}}$	$0.04309^{\{5\}}$	$0.05568^{\{8\}}$	$0.04331^{\{7\}}$	$0.05896^{\{10\}}$	$0.03927^{\{4\}}$	$0.05573^{\{9\}}$
		$\hat{b}$	$0.0635^{\{1\}}$	$0.07605^{\{2\}}$	$0.08051^{\{3\}}$	$0.08635^{\{6\}}$	$0.08546^{\{5\}}$	$0.10005^{\{9\}}$	$0.09082^{\{7\}}$	$0.1048^{\{10\}}$	$0.08494^{\{4\}}$	$0.09922^{\{8\}}$
		$\hat{c}$	$0.04211^{\{1\}}$	$0.05529^{\{4\}}$	$0.07231^{\{9\}}$	$0.05005^{\{3\}}$	$0.07468^{\{10\}}$	$0.07215^{\{8\}}$	$0.05961^{\{6\}}$	$0.07199^{\{7\}}$	$0.04998^{\{2\}}$	$0.05898^{\{5\}}$
	MRE	$\hat{a}$	$0.09059^{\{1\}}$	$0.09667^{\{2\}}$	$0.11224^{\{7\}}$	$0.10047^{\{3\}}$	$0.11184^{\{6\}}$	$0.12453^{\{8\}}$	$0.11045^{\{5\}}$	$0.12924^{\{10\}}$	$0.10568^{\{4\}}$	$0.12458^{\{9\}}$
		$\hat{b}$	$0.26557^{\{1\}}$	$0.29089^{\{2\}}$	$0.30425^{\{3\}}$	$0.31387^{\{6\}}$	$0.31252^{\{4\}}$	$0.34351^{\{8.5\}}$	$0.32533^{\{7\}}$	$0.35691^{\{10\}}$	$0.31333^{\{5\}}$	$0.34351^{\{8.5\}}$
		$\hat{c}$	$0.07579^{\{1\}}$	$0.08385^{\{3\}}$	$0.09503^{\{8\}}$	$0.07972^{\{2\}}$	$0.09396^{\{7\}}$	$0.09974^{\{9\}}$	$0.0911^{\{5\}}$	$0.10146^{\{10\}}$	$0.08406^{\{4\}}$	$0.09151^{\{6\}}$
	D_{abs}		$0.00994^{\{3\}}$	$0.00986^{\{1\}}$	$0.0106^{\{5\}}$	$0.00987^{\{2\}}$	$0.01044^{\{4\}}$	$0.01567^{\{8\}}$	$0.01336^{\{7\}}$	$0.01684^{\{9\}}$	$0.01246^{\{6\}}$	$0.01685^{\{10\}}$
	$D_{\max}$		$0.01673^{\{1\}}$	$0.01675^{\{2\}}$	$0.01825^{\{5\}}$	$0.01679^{\{3\}}$	$0.01788^{\{4\}}$	$0.02585^{\{8\}}$	$0.02217^{\{7\}}$	$0.02758^{\{10\}}$	$0.0207^{\{6\}}$	$0.0274^{\{9\}}$
	ASAE		$0.0104^{\{5\}}$	$0.00988^{\{3\}}$	$0.00985^{\{2\}}$	$0.01035^{\{4\}}$	$0.00978^{\{1\}}$	$0.01489^{\{8\}}$	$0.01379^{\{7\}}$	$0.01652^{\{10\}}$	$0.01264^{\{6\}}$	$0.0165^{\{9\}}$
	$\sum$ Ranks		$18^{\{1\}}$	$28^{\{2\}}$	$66^{\{6\}}$	$43^{\{3\}}$	$63^{\{5\}}$	$100.5^{\{9\}}$	$75^{\{7\}}$	$116^{\{10\}}$	$54^{\{4\}}$	$96.5^{\{8\}}$
400	BIAS	$\hat{a}$	$0.11045^{\{1\}}$	$0.12333^{\{2\}}$	$0.13908^{\{6\}}$	$0.12745^{\{3\}}$	$0.13254^{\{4\}}$	$0.15651^{\{8\}}$	$0.13695^{\{5\}}$	$0.16368^{\{10\}}$	$0.13977^{\{7\}}$	$0.16228^{\{9\}}$
		$\hat{b}$	$0.16436^{\{1\}}$	$0.18541^{\{2\}}$	$0.19621^{\{4\}}$	$0.2021^{\{5\}}$	$0.19565^{\{3\}}$	$0.22846^{\{8\}}$	$0.21466^{\{7\}}$	$0.24137^{\{9\}}$	$0.2142^{\{6\}}$	$0.24618^{\{10\}}$
		$\hat{c}$	$0.13717^{\{1\}}$	$0.14719^{\{3\}}$	$0.16945^{\{7\}}$	$0.14262^{\{2\}}$	$0.16416^{\{6\}}$	$0.17457^{\{10\}}$	$0.16107^{\{5\}}$	$0.17285^{\{9\}}$	$0.15079^{\{4\}}$	$0.16984^{\{8\}}$
	MSE	$\hat{a}$	$0.01895^{\{1\}}$	$0.02326^{\{2\}}$	$0.03065^{\{7\}}$	$0.02628^{\{3\}}$	$0.02687^{\{4\}}$	$0.03754^{\{8\}}$	$0.02987^{\{5\}}$	$0.04157^{\{9\}}$	$0.03008^{\{6\}}$	$0.04193^{\{10\}}$
		$\hat{b}$	$0.04607^{\{1\}}$	$0.05772^{\{2\}}$	$0.06579^{\{4\}}$	$0.06727^{\{5\}}$	$0.06145^{\{3\}}$	$0.08074^{\{8\}}$	$0.07246^{\{7\}}$	$0.09093^{\{9\}}$	$0.07232^{\{6\}}$	$0.09171^{\{10\}}$
		$\hat{c}$	$0.03248^{\{1\}}$	$0.04044^{\{2\}}$	$0.05947^{\{9\}}$	$0.04113^{\{3\}}$	$0.05404^{\{6\}}$	$0.0607^{\{10\}}$	$0.04848^{\{5\}}$	$0.05447^{\{8\}}$	$0.04196^{\{4\}}$	$0.05416^{\{7\}}$
	MRE	$\hat{a}$	$0.07363^{\{1\}}$	$0.08222^{\{2\}}$	$0.09272^{\{6\}}$	$0.08496^{\{3\}}$	$0.08836^{\{4\}}$	$0.10434^{\{8\}}$	$0.0913^{\{5\}}$	$0.10912^{\{10\}}$	$0.09318^{\{7\}}$	$0.10819^{\{9\}}$
		$\hat{b}$	$0.21914^{\{1\}}$	$0.24721^{\{2\}}$	$0.26161^{\{4\}}$	$0.26946^{\{5\}}$	$0.26087^{\{3\}}$	$0.30462^{\{8\}}$	$0.28622^{\{7\}}$	$0.32183^{\{9\}}$	$0.2856^{\{6\}}$	$0.32824^{\{10\}}$
		$\hat{c}$	$0.06859^{\{1\}}$	$0.07359^{\{3\}}$	$0.08472^{\{7\}}$	$0.07131^{\{2\}}$	$0.08208^{\{6\}}$	$0.08728^{\{10\}}$	$0.08054^{\{5\}}$	$0.08643^{\{9\}}$	$0.07539^{\{4\}}$	$0.08492^{\{8\}}$
	D_{abs}		$0.00802^{\{2.5\}}$	$0.00783^{\{1\}}$	$0.00871^{\{5\}}$	$0.00802^{\{2.5\}}$	$0.00863^{\{4\}}$	$0.01232^{\{8\}}$	$0.01089^{\{7\}}$	$0.0139^{\{10\}}$	$0.0098^{\{6\}}$	$0.01332^{\{9\}}$
	$D_{\max}$		$0.01351^{\{2\}}$	$0.01344^{\{1\}}$	$0.01501^{\{5\}}$	$0.01377^{\{3\}}$	$0.01486^{\{4\}}$	$0.02043^{\{8\}}$	$0.01804^{\{7\}}$	$0.02281^{\{10\}}$	$0.01657^{\{6\}}$	$0.02211^{\{9\}}$
	ASAE		$0.00813^{\{4\}}$	$0.00753^{\{1\}}$	$0.00773^{\{3\}}$	$0.00819^{\{5\}}$	$0.00764^{\{2\}}$	$0.01201^{\{8\}}$	$0.0108^{\{7\}}$	$0.01327^{\{10\}}$	$0.00989^{\{6\}}$	$0.01308^{\{9\}}$
	$\sum$ Ranks		$17.5^{\{1\}}$	$23^{\{2\}}$	$67^{\{5\}}$	$41.5^{\{3\}}$	$49^{\{4\}}$	$102^{\{8\}}$	$72^{\{7\}}$	$112^{\{10\}}$	$68^{\{6\}}$	$108^{\{9\}}$

Table 3: Numerical values for some measures for $a = 0.25,\ b = 2.5,\ c = 1.5$ under SRS.

$s^\bullet$	Measure	Est.	ML	AD	CRM	MXPS	LS	SPAD	SPALoD	MSSD	MSSLD	MSLND
30	BIAS	$\hat{a}$	$0.11575^{\{3\}}$	$0.11804^{\{8\}}$	$0.11581^{\{4\}}$	$0.11451^{\{2\}}$	$0.11664^{\{6\}}$	$0.11445^{\{1\}}$	$0.11677^{\{7\}}$	$0.11831^{\{9\}}$	$0.11619^{\{5\}}$	$0.11919^{\{10\}}$
		$\hat{b}$	$0.73637^{\{7\}}$	$0.72846^{\{4\}}$	$0.72929^{\{5\}}$	$0.67953^{\{1\}}$	$0.74655^{\{9\}}$	$0.72425^{\{3\}}$	$0.76813^{\{10\}}$	$0.73935^{\{8\}}$	$0.72113^{\{2\}}$	$0.73242^{\{6\}}$
		$\hat{c}$	$0.63497^{\{8\}}$	$0.60868^{\{3\}}$	$0.59628^{\{1\}}$	$0.60258^{\{2\}}$	$0.60998^{\{4\}}$	$0.63263^{\{6\}}$	$0.64489^{\{10\}}$	$0.63193^{\{5\}}$	$0.6347^{\{7\}}$	$0.63934^{\{9\}}$
	MSE	$\hat{a}$	$0.01831^{\{3\}}$	$0.019^{\{9\}}$	$0.01834^{\{4\}}$	$0.01775^{\{1\}}$	$0.01843^{\{7\}}$	$0.01778^{\{2\}}$	$0.01841^{\{6\}}$	$0.01898^{\{8\}}$	$0.01836^{\{5\}}$	$0.01935^{\{10\}}$
		$\hat{b}$	$0.81982^{\{4\}}$	$0.83065^{\{5\}}$	$0.84281^{\{6\}}$	$0.71765^{\{1\}}$	$0.87562^{\{8\}}$	$0.88331^{\{9\}}$	$0.92317^{\{10\}}$	$0.84461^{\{7\}}$	$0.81258^{\{2\}}$	$0.81889^{\{3\}}$
		$\hat{c}$	$0.5542^{\{5\}}$	$0.52971^{\{3\}}$	$0.50998^{\{1\}}$	$0.5119^{\{2\}}$	$0.53223^{\{4\}}$	$0.57507^{\{9\}}$	$0.58655^{\{10\}}$	$0.55863^{\{6\}}$	$0.57018^{\{8\}}$	$0.56202^{\{7\}}$
	MRE	$\hat{a}$	$0.46301^{\{3\}}$	$0.47215^{\{8\}}$	$0.46326^{\{4\}}$	$0.45805^{\{2\}}$	$0.46654^{\{6\}}$	$0.45778^{\{1\}}$	$0.46708^{\{7\}}$	$0.47323^{\{9\}}$	$0.46478^{\{5\}}$	$0.47675^{\{10\}}$
		$\hat{b}$	$0.29455^{\{7\}}$	$0.29138^{\{4\}}$	$0.29172^{\{5\}}$	$0.27181^{\{1\}}$	$0.29862^{\{9\}}$	$0.2897^{\{3\}}$	$0.30725^{\{10\}}$	$0.29574^{\{8\}}$	$0.28845^{\{2\}}$	$0.29297^{\{6\}}$
		$\hat{c}$	$0.42331^{\{8\}}$	$0.40579^{\{3\}}$	$0.39752^{\{1\}}$	$0.40172^{\{2\}}$	$0.40665^{\{4\}}$	$0.42176^{\{6\}}$	$0.42993^{\{10\}}$	$0.42129^{\{5\}}$	$0.42313^{\{7\}}$	$0.42623^{\{9\}}$
	D_{abs}		$0.03631^{\{1\}}$	$0.03754^{\{4\}}$	$0.03653^{\{2\}}$	$0.03745^{\{3\}}$	$0.03833^{\{5\}}$	$0.04197^{\{8\}}$	$0.04187^{\{7\}}$	$0.04519^{\{9\}}$	$0.03974^{\{6\}}$	$0.04659^{\{10\}}$
	$D_{\max}$		$0.0563^{\{3\}}$	$0.05674^{\{4\}}$	$0.05585^{\{2\}}$	$0.05565^{\{1\}}$	$0.0572^{\{5\}}$	$0.06243^{\{7.5\}}$	$0.06243^{\{7.5\}}$	$0.06623^{\{9\}}$	$0.05972^{\{6\}}$	$0.0679^{\{10\}}$
	ASAE		$0.03631^{\{5\}}$	$0.03214^{\{3\}}$	$0.03179^{\{2\}}$	$0.03385^{\{4\}}$	$0.03058^{\{1\}}$	$0.04781^{\{10\}}$	$0.045^{\{7\}}$	$0.04775^{\{9\}}$	$0.04085^{\{6\}}$	$0.04739^{\{8\}}$
	$\sum$ Ranks		$57^{\{3\}}$	$58^{\{4\}}$	$37^{\{2\}}$	$22^{\{1\}}$	$68^{\{7\}}$	$65.5^{\{6\}}$	$101.5^{\{10\}}$	$92^{\{8\}}$	$61^{\{5\}}$	$98^{\{9\}}$
75	BIAS	$\hat{a}$	$0.11098^{\{6\}}$	$0.11324^{\{9\}}$	$0.11492^{\{10\}}$	$0.11076^{\{5\}}$	$0.10721^{\{1\}}$	$0.111^{\{7\}}$	$0.11046^{\{4\}}$	$0.11014^{\{2\}}$	$0.11019^{\{3\}}$	$0.1114^{\{8\}}$
		$\hat{b}$	$0.61912^{\{3\}}$	$0.67081^{\{10\}}$	$0.65086^{\{8\}}$	$0.61066^{\{2\}}$	$0.62155^{\{4\}}$	$0.6061^{\{1\}}$	$0.63339^{\{6\}}$	$0.64413^{\{7\}}$	$0.629^{\{5\}}$	$0.66988^{\{9\}}$
		$\hat{c}$	$0.55724^{\{7\}}$	$0.55719^{\{6\}}$	$0.55594^{\{4\}}$	$0.54828^{\{2\}}$	$0.53321^{\{1\}}$	$0.54951^{\{3\}}$	$0.55787^{\{8\}}$	$0.5885^{\{10\}}$	$0.55699^{\{5\}}$	$0.56064^{\{9\}}$
	MSE	$\hat{a}$	$0.0171^{\{5.5\}}$	$0.0176^{\{9\}}$	$0.01829^{\{10\}}$	$0.01681^{\{2\}}$	$0.01615^{\{1\}}$	$0.0171^{\{5.5\}}$	$0.01705^{\{4\}}$	$0.0169^{\{3\}}$	$0.01711^{\{7\}}$	$0.01725^{\{8\}}$
		$\hat{b}$	$0.60061^{\{2\}}$	$0.71688^{\{10\}}$	$0.67918^{\{8\}}$	$0.59578^{\{1\}}$	$0.64537^{\{7\}}$	$0.60268^{\{3\}}$	$0.64092^{\{5\}}$	$0.64262^{\{6\}}$	$0.61979^{\{4\}}$	$0.69586^{\{9\}}$
		$\hat{c}$	$0.44387^{\{3\}}$	$0.45333^{\{6\}}$	$0.45805^{\{8\}}$	$0.4381^{\{2\}}$	$0.42094^{\{1\}}$	$0.45766^{\{7\}}$	$0.45067^{\{4\}}$	$0.48572^{\{10\}}$	$0.45168^{\{5\}}$	$0.46036^{\{9\}}$
	MRE	$\hat{a}$	$0.4439^{\{6\}}$	$0.45298^{\{9\}}$	$0.45966^{\{10\}}$	$0.44303^{\{5\}}$	$0.42883^{\{1\}}$	$0.44398^{\{7\}}$	$0.44183^{\{4\}}$	$0.44055^{\{2\}}$	$0.44074^{\{3\}}$	$0.44561^{\{8\}}$
		$\hat{b}$	$0.24765^{\{3\}}$	$0.26832^{\{10\}}$	$0.26034^{\{8\}}$	$0.24426^{\{2\}}$	$0.24862^{\{4\}}$	$0.24244^{\{1\}}$	$0.25335^{\{6\}}$	$0.25765^{\{7\}}$	$0.2516^{\{5\}}$	$0.26795^{\{9\}}$
		$\hat{c}$	$0.37149^{\{7\}}$	$0.37146^{\{6\}}$	$0.37062^{\{4\}}$	$0.36552^{\{2\}}$	$0.35548^{\{1\}}$	$0.36634^{\{3\}}$	$0.37191^{\{8\}}$	$0.39233^{\{10\}}$	$0.37132^{\{5\}}$	$0.37376^{\{9\}}$
	D_{abs}		$0.0254^{\{1\}}$	$0.02669^{\{3\}}$	$0.02713^{\{5\}}$	$0.02626^{\{2\}}$	$0.0271^{\{4\}}$	$0.03044^{\{7\}}$	$0.03046^{\{8\}}$	$0.03461^{\{9\}}$	$0.02883^{\{6\}}$	$0.03494^{\{10\}}$
	$D_{\max}$		$0.0398^{\{1\}}$	$0.04143^{\{3\}}$	$0.04234^{\{5\}}$	$0.04019^{\{2\}}$	$0.04168^{\{4\}}$	$0.0462^{\{7\}}$	$0.04627^{\{8\}}$	$0.05193^{\{9\}}$	$0.04408^{\{6\}}$	$0.05236^{\{10\}}$
	ASAE		$0.02197^{\{5\}}$	$0.0194^{\{3\}}$	$0.01908^{\{2\}}$	$0.02085^{\{4\}}$	$0.019^{\{1\}}$	$0.03015^{\{8\}}$	$0.02773^{\{7\}}$	$0.0319^{\{10\}}$	$0.02602^{\{6\}}$	$0.03152^{\{9\}}$
	$\sum$ Ranks		$49.5^{\{3\}}$	$84^{\{8\}}$	$82^{\{7\}}$	$31^{\{2\}}$	$30^{\{1\}}$	$59.5^{\{4\}}$	$72^{\{6\}}$	$85^{\{9\}}$	$60^{\{5\}}$	$107^{\{10\}}$
150	BIAS	$\hat{a}$	$0.10796^{\{9\}}$	$0.10716^{\{6\}}$	$0.10902^{\{10\}}$	$0.09898^{\{1\}}$	$0.10525^{\{5\}}$	$0.10324^{\{2\}}$	$0.10457^{\{3\}}$	$0.10769^{\{7\}}$	$0.10514^{\{4\}}$	$0.10795^{\{8\}}$
		$\hat{b}$	$0.55729^{\{2\}}$	$0.58029^{\{8\}}$	$0.57819^{\{7\}}$	$0.53634^{\{1\}}$	$0.58485^{\{9\}}$	$0.57008^{\{4\}}$	$0.57659^{\{5\}}$	$0.60469^{\{10\}}$	$0.56471^{\{3\}}$	$0.57724^{\{6\}}$
		$\hat{c}$	$0.51037^{\{7\}}$	$0.51159^{\{8\}}$	$0.49818^{\{3\}}$	$0.4774^{\{1\}}$	$0.49359^{\{2\}}$	$0.51573^{\{10\}}$	$0.51174^{\{9\}}$	$0.50934^{\{6\}}$	$0.50903^{\{5\}}$	$0.50763^{\{4\}}$
	MSE	$\hat{a}$	$0.01627^{\{7\}}$	$0.01634^{\{8.5\}}$	$0.01646^{\{10\}}$	$0.01421^{\{1\}}$	$0.01561^{\{5\}}$	$0.01523^{\{2\}}$	$0.01542^{\{3\}}$	$0.01634^{\{8.5\}}$	$0.01556^{\{4\}}$	$0.01617^{\{6\}}$
		$\hat{b}$	$0.49952^{\{2\}}$	$0.53992^{\{6\}}$	$0.55045^{\{8\}}$	$0.47735^{\{1\}}$	$0.57014^{\{9\}}$	$0.53101^{\{5\}}$	$0.5454^{\{7\}}$	$0.58431^{\{10\}}$	$0.50157^{\{3\}}$	$0.52626^{\{4\}}$
		$\hat{c}$	$0.3867^{\{7\}}$	$0.38794^{\{8\}}$	$0.37553^{\{3\}}$	$0.34518^{\{1\}}$	$0.3666^{\{2\}}$	$0.39463^{\{10\}}$	$0.39247^{\{9\}}$	$0.38371^{\{5\}}$	$0.37689^{\{4\}}$	$0.38595^{\{6\}}$
	MRE	$\hat{a}$	$0.43185^{\{9\}}$	$0.42865^{\{6\}}$	$0.43608^{\{10\}}$	$0.39593^{\{1\}}$	$0.421^{\{5\}}$	$0.41297^{\{2\}}$	$0.41827^{\{3\}}$	$0.43078^{\{7\}}$	$0.42057^{\{4\}}$	$0.4318^{\{8\}}$
		$\hat{b}$	$0.22292^{\{2\}}$	$0.23212^{\{8\}}$	$0.23128^{\{7\}}$	$0.21454^{\{1\}}$	$0.23394^{\{9\}}$	$0.22803^{\{4\}}$	$0.23063^{\{5\}}$	$0.24187^{\{10\}}$	$0.22588^{\{3\}}$	$0.23089^{\{6\}}$
		$\hat{c}$	$0.34025^{\{7\}}$	$0.34106^{\{8\}}$	$0.33212^{\{3\}}$	$0.31826^{\{1\}}$	$0.32906^{\{2\}}$	$0.34382^{\{10\}}$	$0.34116^{\{9\}}$	$0.33956^{\{6\}}$	$0.33935^{\{5\}}$	$0.33842^{\{4\}}$
	D_{abs}		$0.01791^{\{1\}}$	$0.01865^{\{3\}}$	$0.01869^{\{4\}}$	$0.01813^{\{2\}}$	$0.01899^{\{5\}}$	$0.0222^{\{8\}}$	$0.0217^{\{7\}}$	$0.02499^{\{9\}}$	$0.01983^{\{6\}}$	$0.02508^{\{10\}}$
	$D_{\max}$		$0.02861^{\{2\}}$	$0.0296^{\{3\}}$	$0.02976^{\{4\}}$	$0.02839^{\{1\}}$	$0.0301^{\{5\}}$	$0.03446^{\{8\}}$	$0.03369^{\{7\}}$	$0.0385^{\{9\}}$	$0.03115^{\{6\}}$	$0.0386^{\{10\}}$
	ASAE		$0.01526^{\{5\}}$	$0.01377^{\{3\}}$	$0.01337^{\{1\}}$	$0.01477^{\{4\}}$	$0.01343^{\{2\}}$	$0.02116^{\{8\}}$	$0.01978^{\{7\}}$	$0.02287^{\{9\}}$	$0.01806^{\{6\}}$	$0.0229^{\{10\}}$
	$\sum$ Ranks		$60^{\{3.5\}}$	$75.5^{\{8\}}$	$70^{\{5\}}$	$16^{\{1\}}$	$60^{\{3.5\}}$	$73^{\{6\}}$	$74^{\{7\}}$	$96.5^{\{10\}}$	$53^{\{2\}}$	$82^{\{9\}}$
250	BIAS	$\hat{a}$	$0.09775^{\{3\}}$	$0.10173^{\{8\}}$	$0.10346^{\{9\}}$	$0.09529^{\{1\}}$	$0.09916^{\{5\}}$	$0.09674^{\{2\}}$	$0.09894^{\{4\}}$	$0.10108^{\{7\}}$	$0.10077^{\{6\}}$	$0.10394^{\{10\}}$
		$\hat{b}$	$0.51949^{\{2\}}$	$0.5579^{\{10\}}$	$0.53648^{\{6\}}$	$0.51644^{\{1\}}$	$0.53932^{\{7\}}$	$0.53032^{\{5\}}$	$0.52863^{\{4\}}$	$0.54011^{\{8\}}$	$0.52143^{\{3\}}$	$0.54444^{\{9\}}$
		$\hat{c}$	$0.45477^{\{2\}}$	$0.4835^{\{9\}}$	$0.45834^{\{3\}}$	$0.4512^{\{1\}}$	$0.46726^{\{4\}}$	$0.48173^{\{8\}}$	$0.49108^{\{10\}}$	$0.47584^{\{6\}}$	$0.47046^{\{5\}}$	$0.47678^{\{7\}}$
	MSE	$\hat{a}$	$0.01351^{\{2\}}$	$0.0147^{\{8\}}$	$0.01494^{\{9\}}$	$0.01319^{\{1\}}$	$0.01417^{\{5\}}$	$0.01369^{\{3\}}$	$0.01412^{\{4\}}$	$0.0146^{\{7\}}$	$0.01451^{\{6\}}$	$0.01542^{\{10\}}$
		$\hat{b}$	$0.44505^{\{2\}}$	$0.52336^{\{10\}}$	$0.49389^{\{8\}}$	$0.44866^{\{3\}}$	$0.51457^{\{9\}}$	$0.47772^{\{7\}}$	$0.46868^{\{4\}}$	$0.47604^{\{6\}}$	$0.44231^{\{1\}}$	$0.47194^{\{5\}}$
		$\hat{c}$	$0.30811^{\{1\}}$	$0.35627^{\{9\}}$	$0.33164^{\{4\}}$	$0.32248^{\{2\}}$	$0.33823^{\{6\}}$	$0.35488^{\{8\}}$	$0.361^{\{10\}}$	$0.34246^{\{7\}}$	$0.33043^{\{3\}}$	$0.33624^{\{5\}}$
	MRE	$\hat{a}$	$0.39098^{\{3\}}$	$0.40691^{\{8\}}$	$0.41383^{\{9\}}$	$0.38117^{\{1\}}$	$0.39665^{\{5\}}$	$0.38697^{\{2\}}$	$0.39576^{\{4\}}$	$0.40434^{\{7\}}$	$0.40309^{\{6\}}$	$0.41577^{\{10\}}$
		$\hat{b}$	$0.20779^{\{2\}}$	$0.22316^{\{10\}}$	$0.21459^{\{6\}}$	$0.20658^{\{1\}}$	$0.21573^{\{7\}}$	$0.21213^{\{5\}}$	$0.21145^{\{4\}}$	$0.21604^{\{8\}}$	$0.20857^{\{3\}}$	$0.21778^{\{9\}}$
		$\hat{c}$	$0.30318^{\{2\}}$	$0.32233^{\{9\}}$	$0.30556^{\{3\}}$	$0.3008^{\{1\}}$	$0.3115^{\{4\}}$	$0.32115^{\{8\}}$	$0.32738^{\{10\}}$	$0.31723^{\{6\}}$	$0.31364^{\{5\}}$	$0.31785^{\{7\}}$
	D_{abs}		$0.01508^{\{3\}}$	$0.01474^{\{2\}}$	$0.01465^{\{1\}}$	$0.01558^{\{5\}}$	$0.01514^{\{4\}}$	$0.0186^{\{8\}}$	$0.0172^{\{7\}}$	$0.01999^{\{10\}}$	$0.01608^{\{6\}}$	$0.01977^{\{9\}}$
	$D_{\max}$		$0.0242^{\{3\}}$	$0.02383^{\{1.5\}}$	$0.02383^{\{1.5\}}$	$0.02464^{\{5\}}$	$0.02424^{\{4\}}$	$0.0291^{\{8\}}$	$0.02725^{\{7\}}$	$0.03113^{\{10\}}$	$0.02561^{\{6\}}$	$0.03088^{\{9\}}$
	ASAE		$0.01184^{\{5\}}$	$0.01063^{\{3\}}$	$0.01025^{\{2\}}$	$0.01144^{\{4\}}$	$0.01019^{\{1\}}$	$0.01652^{\{8\}}$	$0.01504^{\{7\}}$	$0.01839^{\{10\}}$	$0.01399^{\{6\}}$	$0.0183^{\{9\}}$
	$\sum$ Ranks		$30^{\{2\}}$	$87.5^{\{8\}}$	$61.5^{\{5\}}$	$26^{\{1\}}$	$61^{\{4\}}$	$72^{\{6\}}$	$75^{\{7\}}$	$92^{\{9\}}$	$56^{\{3\}}$	$99^{\{10\}}$
400	BIAS	$\hat{a}$	$0.08796^{\{1\}}$	$0.09034^{\{3\}}$	$0.09709^{\{7\}}$	$0.08932^{\{2\}}$	$0.09884^{\{8\}}$	$0.09278^{\{6\}}$	$0.09176^{\{5\}}$	$0.10092^{\{9\}}$	$0.09155^{\{4\}}$	$0.1017^{\{10\}}$
		$\hat{b}$	$0.4767^{\{2\}}$	$0.49209^{\{4\}}$	$0.51626^{\{7\}}$	$0.47612^{\{1\}}$	$0.52164^{\{10\}}$	$0.51453^{\{6\}}$	$0.48467^{\{3\}}$	$0.51685^{\{8\}}$	$0.49278^{\{5\}}$	$0.52113^{\{9\}}$
		$\hat{c}$	$0.42594^{\{2\}}$	$0.42951^{\{3\}}$	$0.43301^{\{5\}}$	$0.40926^{\{1\}}$	$0.45444^{\{9\}}$	$0.45285^{\{8\}}$	$0.43029^{\{4\}}$	$0.47236^{\{10\}}$	$0.43607^{\{6\}}$	$0.4504^{\{7\}}$
	MSE	$\hat{a}$	$0.01151^{\{1\}}$	$0.01186^{\{3\}}$	$0.01332^{\{7\}}$	$0.01181^{\{2\}}$	$0.01391^{\{8\}}$	$0.01283^{\{6\}}$	$0.01252^{\{5\}}$	$0.01445^{\{9\}}$	$0.01227^{\{4\}}$	$0.01464^{\{10\}}$
		$\hat{b}$	$0.39176^{\{1\}}$	$0.42778^{\{5\}}$	$0.47648^{\{10\}}$	$0.40636^{\{2\}}$	$0.47588^{\{9\}}$	$0.45343^{\{8\}}$	$0.42085^{\{4\}}$	$0.44038^{\{6\}}$	$0.41893^{\{3\}}$	$0.44717^{\{7\}}$
		$\hat{c}$	$0.2801^{\{2\}}$	$0.29292^{\{3\}}$	$0.30042^{\{6\}}$	$0.26095^{\{1\}}$	$0.32466^{\{9\}}$	$0.31518^{\{8\}}$	$0.29713^{\{5\}}$	$0.32998^{\{10\}}$	$0.29385^{\{4\}}$	$0.30915^{\{7\}}$
	MRE	$\hat{a}$	$0.35185^{\{1\}}$	$0.36136^{\{3\}}$	$0.38835^{\{7\}}$	$0.35729^{\{2\}}$	$0.39535^{\{8\}}$	$0.37112^{\{6\}}$	$0.36704^{\{5\}}$	$0.40367^{\{9\}}$	$0.36619^{\{4\}}$	$0.40679^{\{10\}}$
		$\hat{b}$	$0.19068^{\{2\}}$	$0.19684^{\{4\}}$	$0.2065^{\{7\}}$	$0.19045^{\{1\}}$	$0.20866^{\{10\}}$	$0.20581^{\{6\}}$	$0.19387^{\{3\}}$	$0.20674^{\{8\}}$	$0.19711^{\{5\}}$	$0.20845^{\{9\}}$
		$\hat{c}$	$0.28396^{\{2\}}$	$0.28634^{\{3\}}$	$0.28868^{\{5\}}$	$0.27284^{\{1\}}$	$0.30296^{\{9\}}$	$0.3019^{\{8\}}$	$0.28686^{\{4\}}$	$0.31491^{\{10\}}$	$0.29071^{\{6\}}$	$0.30026^{\{7\}}$
	D_{abs}		$0.01179^{\{1\}}$	$0.01191^{\{3\}}$	$0.01264^{\{5\}}$	$0.01186^{\{2\}}$	$0.01252^{\{4\}}$	$0.01418^{\{8\}}$	$0.01359^{\{7\}}$	$0.01565^{\{9\}}$	$0.01325^{\{6\}}$	$0.01566^{\{10\}}$
	$D_{\max}$		$0.01921^{\{2\}}$	$0.01941^{\{3\}}$	$0.02072^{\{5\}}$	$0.01916^{\{1\}}$	$0.02062^{\{4\}}$	$0.02269^{\{8\}}$	$0.02172^{\{7\}}$	$0.02498^{\{9.5\}}$	$0.0213^{\{6\}}$	$0.02498^{\{9.5\}}$
	ASAE		$0.00905^{\{4\}}$	$0.0085^{\{3\}}$	$0.00819^{\{2\}}$	$0.00912^{\{5\}}$	$0.00815^{\{1\}}$	$0.01304^{\{8\}}$	$0.01185^{\{7\}}$	$0.01445^{\{9.5\}}$	$0.01133^{\{6\}}$	$0.01445^{\{9.5\}}$
	$\sum$ Ranks		$21^{\{1.5\}}$	$40^{\{3\}}$	$73^{\{6\}}$	$21^{\{1.5\}}$	$89^{\{8\}}$	$86^{\{7\}}$	$59^{\{4.5\}}$	$107^{\{10\}}$	$59^{\{4.5\}}$	$105^{\{9\}}$

Table 4: Numerical values for some measures for $a = 0.25,\ b = 2.5,\ c = 1.5$ under RSS.

$s^{\bullet}$	Measure	Est.	ML	AD	CRM	MXPS	LS	SPAD	SPALoD	MSSD	MSSLD	MSLND
30	BIAS	$\hat{a}$	$0.11047^{\{2\}}$	$0.1108^{\{4\}}$	$0.11442^{\{7\}}$	$0.10909^{\{1\}}$	$0.11368^{\{6\}}$	$0.11048^{\{3\}}$	$0.11088^{\{5\}}$	$0.11624^{\{10\}}$	$0.11554^{\{8\}}$	$0.11555^{\{9\}}$
		$\hat{b}$	$0.60901^{\{2\}}$	$0.64892^{\{7\}}$	$0.63236^{\{4\}}$	$0.58154^{\{1\}}$	$0.6286^{\{3\}}$	$0.66384^{\{8\}}$	$0.63387^{\{5\}}$	$0.68667^{\{10\}}$	$0.63627^{\{6\}}$	$0.67321^{\{9\}}$
		$\hat{c}$	$0.54193^{\{5\}}$	$0.53046^{\{4\}}$	$0.52682^{\{3\}}$	$0.51508^{\{2\}}$	$0.50527^{\{1\}}$	$0.57206^{\{8\}}$	$0.55691^{\{6\}}$	$0.5867^{\{9\}}$	$0.5607^{\{7\}}$	$0.59405^{\{10\}}$
	MSE	$\hat{a}$	$0.01713^{\{5\}}$	$0.01673^{\{2\}}$	$0.01816^{\{10\}}$	$0.01645^{\{1\}}$	$0.01744^{\{6\}}$	$0.0169^{\{3\}}$	$0.01711^{\{4\}}$	$0.01814^{\{8\}}$	$0.01815^{\{9\}}$	$0.01808^{\{7\}}$
		$\hat{b}$	$0.5806^{\{2\}}$	$0.66411^{\{6\}}$	$0.64274^{\{5\}}$	$0.53968^{\{1\}}$	$0.64122^{\{4\}}$	$0.71966^{\{9\}}$	$0.66524^{\{7\}}$	$0.74225^{\{10\}}$	$0.62388^{\{3\}}$	$0.71658^{\{8\}}$
		$\hat{c}$	$0.43679^{\{5\}}$	$0.42632^{\{4\}}$	$0.41522^{\{3\}}$	$0.38748^{\{1\}}$	$0.39124^{\{2\}}$	$0.47989^{\{8\}}$	$0.46247^{\{7\}}$	$0.49911^{\{9\}}$	$0.45524^{\{6\}}$	$0.50212^{\{10\}}$
	MRE	$\hat{a}$	$0.4419^{\{2\}}$	$0.44322^{\{4\}}$	$0.4577^{\{7\}}$	$0.43637^{\{1\}}$	$0.45473^{\{6\}}$	$0.44193^{\{3\}}$	$0.44353^{\{5\}}$	$0.46495^{\{10\}}$	$0.46218^{\{8\}}$	$0.4622^{\{9\}}$
		$\hat{b}$	$0.2436^{\{2\}}$	$0.25957^{\{7\}}$	$0.25294^{\{4\}}$	$0.23262^{\{1\}}$	$0.25144^{\{3\}}$	$0.26554^{\{8\}}$	$0.25355^{\{5\}}$	$0.27467^{\{10\}}$	$0.25451^{\{6\}}$	$0.26929^{\{9\}}$
		$\hat{c}$	$0.36129^{\{5\}}$	$0.35364^{\{4\}}$	$0.35122^{\{3\}}$	$0.34339^{\{2\}}$	$0.33684^{\{1\}}$	$0.38137^{\{8\}}$	$0.37127^{\{6\}}$	$0.39113^{\{9\}}$	$0.3738^{\{7\}}$	$0.39603^{\{10\}}$
	D_{abs}		$0.02346^{\{1\}}$	$0.02471^{\{3\}}$	$0.02573^{\{5\}}$	$0.02377^{\{2\}}$	$0.02565^{\{4\}}$	$0.03465^{\{8\}}$	$0.03046^{\{7\}}$	$0.03766^{\{9\}}$	$0.02847^{\{6\}}$	$0.03855^{\{10\}}$
	$D_{\max}$		$0.03729^{\{2\}}$	$0.03818^{\{3\}}$	$0.03985^{\{5\}}$	$0.03632^{\{1\}}$	$0.039^{\{4\}}$	$0.05193^{\{8\}}$	$0.0462^{\{7\}}$	$0.05558^{\{9\}}$	$0.04373^{\{6\}}$	$0.05667^{\{10\}}$
	ASAE		$0.03523^{\{5\}}$	$0.03167^{\{3\}}$	$0.03064^{\{2\}}$	$0.03357^{\{4\}}$	$0.03058^{\{1\}}$	$0.04719^{\{10\}}$	$0.04367^{\{7\}}$	$0.04517^{\{8\}}$	$0.03965^{\{6\}}$	$0.04684^{\{9\}}$
	$\sum$ Ranks		$38^{\{2\}}$	$51^{\{4\}}$	$58^{\{5\}}$	$18^{\{1\}}$	$41^{\{3\}}$	$84^{\{8\}}$	$71^{\{6\}}$	$111^{\{10\}}$	$78^{\{7\}}$	$110^{\{9\}}$
75	BIAS	$\hat{a}$	$0.10791^{\{5\}}$	$0.1085^{\{7\}}$	$0.11036^{\{10\}}$	$0.10626^{\{3\}}$	$0.10605^{\{2\}}$	$0.10587^{\{1\}}$	$0.10815^{\{6\}}$	$0.10998^{\{9\}}$	$0.107^{\{4\}}$	$0.10862^{\{8\}}$
		$\hat{b}$	$0.56962^{\{8\}}$	$0.56477^{\{6\}}$	$0.55825^{\{3\}}$	$0.53221^{\{1\}}$	$0.56554^{\{7\}}$	$0.55891^{\{4\}}$	$0.56117^{\{5\}}$	$0.61718^{\{10\}}$	$0.55579^{\{2\}}$	$0.60509^{\{9\}}$
		$\hat{c}$	$0.49398^{\{4\}}$	$0.4727^{\{1\}}$	$0.48536^{\{3\}}$	$0.48518^{\{2\}}$	$0.49745^{\{5\}}$	$0.52042^{\{10\}}$	$0.51465^{\{7\}}$	$0.51818^{\{9\}}$	$0.49973^{\{6\}}$	$0.51726^{\{8\}}$
	MSE	$\hat{a}$	$0.01614^{\{4\}}$	$0.01616^{\{6\}}$	$0.01689^{\{10\}}$	$0.01589^{\{2\}}$	$0.01601^{\{3\}}$	$0.01582^{\{1\}}$	$0.01628^{\{7\}}$	$0.0168^{\{9\}}$	$0.01615^{\{5\}}$	$0.01649^{\{8\}}$
		$\hat{b}$	$0.5479^{\{7\}}$	$0.53439^{\{6\}}$	$0.52771^{\{5\}}$	$0.45342^{\{1\}}$	$0.5596^{\{8\}}$	$0.52305^{\{4\}}$	$0.51382^{\{3\}}$	$0.58741^{\{10\}}$	$0.50519^{\{2\}}$	$0.57297^{\{9\}}$
		$\hat{c}$	$0.37028^{\{4\}}$	$0.35845^{\{3\}}$	$0.35607^{\{2\}}$	$0.35293^{\{1\}}$	$0.38572^{\{6\}}$	$0.41038^{\{10\}}$	$0.40084^{\{8\}}$	$0.40115^{\{9\}}$	$0.37444^{\{5\}}$	$0.39235^{\{7\}}$
	MRE	$\hat{a}$	$0.43163^{\{5\}}$	$0.43401^{\{7\}}$	$0.44143^{\{10\}}$	$0.42502^{\{3\}}$	$0.42418^{\{2\}}$	$0.42349^{\{1\}}$	$0.43262^{\{6\}}$	$0.43991^{\{9\}}$	$0.42801^{\{4\}}$	$0.43449^{\{8\}}$
		$\hat{b}$	$0.22785^{\{8\}}$	$0.22591^{\{6\}}$	$0.2233^{\{3\}}$	$0.21288^{\{1\}}$	$0.22622^{\{7\}}$	$0.22356^{\{4\}}$	$0.22447^{\{5\}}$	$0.24687^{\{10\}}$	$0.22232^{\{2\}}$	$0.24204^{\{9\}}$
		$\hat{c}$	$0.32932^{\{4\}}$	$0.31513^{\{1\}}$	$0.32357^{\{3\}}$	$0.32345^{\{2\}}$	$0.33164^{\{5\}}$	$0.34694^{\{10\}}$	$0.3431^{\{7\}}$	$0.34545^{\{9\}}$	$0.33316^{\{6\}}$	$0.34484^{\{8\}}$
	D_{abs}		$0.01598^{\{1\}}$	$0.01626^{\{3\}}$	$0.01671^{\{4\}}$	$0.01615^{\{2\}}$	$0.01695^{\{5\}}$	$0.02429^{\{8\}}$	$0.0212^{\{7\}}$	$0.02775^{\{10\}}$	$0.01996^{\{6\}}$	$0.02767^{\{9\}}$
	$D_{\max}$		$0.02588^{\{2.5\}}$	$0.02588^{\{2.5\}}$	$0.02666^{\{4\}}$	$0.02567^{\{1\}}$	$0.0269^{\{5\}}$	$0.03728^{\{8\}}$	$0.03301^{\{7\}}$	$0.04221^{\{10\}}$	$0.03117^{\{6\}}$	$0.04204^{\{9\}}$
	ASAE		$0.02113^{\{5\}}$	$0.01938^{\{3\}}$	$0.01869^{\{2\}}$	$0.02075^{\{4\}}$	$0.01866^{\{1\}}$	$0.02967^{\{8\}}$	$0.02725^{\{7\}}$	$0.03127^{\{10\}}$	$0.02526^{\{6\}}$	$0.03103^{\{9\}}$
	$\sum$ Ranks		$57.5^{\{5\}}$	$51.5^{\{2\}}$	$59^{\{6\}}$	$23^{\{1\}}$	$56^{\{4\}}$	$69^{\{7\}}$	$75^{\{8\}}$	$114^{\{10\}}$	$54^{\{3\}}$	$101^{\{9\}}$
150	BIAS	$\hat{a}$	$0.09783^{\{2\}}$	$0.10212^{\{6\}}$	$0.1035^{\{7\}}$	$0.09773^{\{1\}}$	$0.10436^{\{8\}}$	$0.10016^{\{4\}}$	$0.10053^{\{5\}}$	$0.10491^{\{9\}}$	$0.10008^{\{3\}}$	$0.10508^{\{10\}}$
		$\hat{b}$	$0.50259^{\{2\}}$	$0.53824^{\{7\}}$	$0.53938^{\{8\}}$	$0.49711^{\{1\}}$	$0.55347^{\{10\}}$	$0.51437^{\{3\}}$	$0.53807^{\{6\}}$	$0.55315^{\{9\}}$	$0.51508^{\{4\}}$	$0.53001^{\{5\}}$
		$\hat{c}$	$0.44167^{\{1\}}$	$0.46353^{\{3\}}$	$0.47301^{\{8\}}$	$0.44351^{\{2\}}$	$0.46409^{\{4\}}$	$0.46918^{\{6\}}$	$0.48526^{\{10\}}$	$0.48467^{\{9\}}$	$0.46634^{\{5\}}$	$0.47021^{\{7\}}$
	MSE	$\hat{a}$	$0.0139^{\{2\}}$	$0.01462^{\{5\}}$	$0.01532^{\{8\}}$	$0.01358^{\{1\}}$	$0.01528^{\{7\}}$	$0.01474^{\{6\}}$	$0.01447^{\{4\}}$	$0.01562^{\{10\}}$	$0.01433^{\{3\}}$	$0.01555^{\{9\}}$
		$\hat{b}$	$0.43456^{\{3\}}$	$0.50532^{\{9\}}$	$0.50402^{\{8\}}$	$0.42049^{\{1\}}$	$0.53416^{\{10\}}$	$0.45122^{\{5\}}$	$0.47284^{\{6\}}$	$0.48821^{\{7\}}$	$0.43445^{\{2\}}$	$0.44975^{\{4\}}$
		$\hat{c}$	$0.31211^{\{2\}}$	$0.33268^{\{5\}}$	$0.3536^{\{9\}}$	$0.29733^{\{1\}}$	$0.33642^{\{6\}}$	$0.34383^{\{7\}}$	$0.35766^{\{10\}}$	$0.34973^{\{8\}}$	$0.32458^{\{3\}}$	$0.33243^{\{4\}}$
	MRE	$\hat{a}$	$0.39133^{\{2\}}$	$0.4085^{\{6\}}$	$0.41399^{\{7\}}$	$0.39091^{\{1\}}$	$0.41746^{\{8\}}$	$0.40065^{\{4\}}$	$0.4021^{\{5\}}$	$0.41962^{\{9\}}$	$0.40033^{\{3\}}$	$0.42032^{\{10\}}$
		$\hat{b}$	$0.20103^{\{2\}}$	$0.21529^{\{7\}}$	$0.21575^{\{8\}}$	$0.19885^{\{1\}}$	$0.22139^{\{10\}}$	$0.20575^{\{3\}}$	$0.21523^{\{6\}}$	$0.22126^{\{9\}}$	$0.20603^{\{4\}}$	$0.21201^{\{5\}}$
		$\hat{c}$	$0.29445^{\{1\}}$	$0.30902^{\{3\}}$	$0.31534^{\{8\}}$	$0.29568^{\{2\}}$	$0.3094^{\{4\}}$	$0.31279^{\{6\}}$	$0.32351^{\{10\}}$	$0.32311^{\{9\}}$	$0.31089^{\{5\}}$	$0.31347^{\{7\}}$
	D_{abs}		$0.01145^{\{2\}}$	$0.01104^{\{1\}}$	$0.0123^{\{5\}}$	$0.01166^{\{3\}}$	$0.01201^{\{4\}}$	$0.01827^{\{8\}}$	$0.01623^{\{7\}}$	$0.01994^{\{10\}}$	$0.01467^{\{6\}}$	$0.01939^{\{9\}}$
	$D_{\max}$		$0.01893^{\{2\}}$	$0.01843^{\{1\}}$	$0.02032^{\{5\}}$	$0.01908^{\{3\}}$	$0.01977^{\{4\}}$	$0.02862^{\{8\}}$	$0.02582^{\{7\}}$	$0.03108^{\{10\}}$	$0.02359^{\{6\}}$	$0.03025^{\{9\}}$
	ASAE		$0.01463^{\{5\}}$	$0.01368^{\{3\}}$	$0.01329^{\{2\}}$	$0.01445^{\{4\}}$	$0.01314^{\{1\}}$	$0.0211^{\{8\}}$	$0.01903^{\{7\}}$	$0.02271^{\{10\}}$	$0.01769^{\{6\}}$	$0.02242^{\{9\}}$
	$\sum$ Ranks		$26^{\{2\}}$	$56^{\{4\}}$	$83^{\{7.5\}}$	$21^{\{1\}}$	$76^{\{6\}}$	$68^{\{5\}}$	$83^{\{7.5\}}$	$109^{\{10\}}$	$50^{\{3\}}$	$88^{\{9\}}$
250	BIAS	$\hat{a}$	$0.0882^{\{1\}}$	$0.09474^{\{3\}}$	$0.09491^{\{4\}}$	$0.09071^{\{2\}}$	$0.09625^{\{7\}}$	$0.0964^{\{8\}}$	$0.09623^{\{6\}}$	$0.10031^{\{9\}}$	$0.09506^{\{5\}}$	$0.10051^{\{10\}}$
		$\hat{b}$	$0.49241^{\{4\}}$	$0.52049^{\{9\}}$	$0.51043^{\{7\}}$	$0.4703^{\{1\}}$	$0.5078^{\{6\}}$	$0.50083^{\{5\}}$	$0.49078^{\{3\}}$	$0.52141^{\{10\}}$	$0.48116^{\{2\}}$	$0.51564^{\{8\}}$
		$\hat{c}$	$0.40474^{\{2\}}$	$0.42638^{\{3\}}$	$0.42802^{\{5\}}$	$0.39272^{\{1\}}$	$0.42682^{\{4\}}$	$0.44052^{\{8\}}$	$0.43556^{\{7\}}$	$0.45595^{\{10\}}$	$0.42935^{\{6\}}$	$0.45136^{\{9\}}$
	MSE	$\hat{a}$	$0.01159^{\{1\}}$	$0.013^{\{3\}}$	$0.01302^{\{4\}}$	$0.01213^{\{2\}}$	$0.01336^{\{6\}}$	$0.01361^{\{8\}}$	$0.0134^{\{7\}}$	$0.01446^{\{9\}}$	$0.01312^{\{5\}}$	$0.01452^{\{10\}}$
		$\hat{b}$	$0.43048^{\{6\}}$	$0.48687^{\{10\}}$	$0.47266^{\{9\}}$	$0.39389^{\{1\}}$	$0.47177^{\{8\}}$	$0.41561^{\{4\}}$	$0.4058^{\{3\}}$	$0.44726^{\{7\}}$	$0.39411^{\{2\}}$	$0.42301^{\{5\}}$
		$\hat{c}$	$0.26351^{\{2\}}$	$0.286^{\{4\}}$	$0.29277^{\{5\}}$	$0.23718^{\{1\}}$	$0.29346^{\{6\}}$	$0.3015^{\{8\}}$	$0.29519^{\{7\}}$	$0.31546^{\{10\}}$	$0.28353^{\{3\}}$	$0.30934^{\{9\}}$
	MRE	$\hat{a}$	$0.35279^{\{1\}}$	$0.37895^{\{3\}}$	$0.37964^{\{4\}}$	$0.36284^{\{2\}}$	$0.38501^{\{7\}}$	$0.38561^{\{8\}}$	$0.38493^{\{6\}}$	$0.40126^{\{9\}}$	$0.38025^{\{5\}}$	$0.40202^{\{10\}}$
		$\hat{b}$	$0.19696^{\{4\}}$	$0.2082^{\{9\}}$	$0.20417^{\{7\}}$	$0.18812^{\{1\}}$	$0.20312^{\{6\}}$	$0.20033^{\{5\}}$	$0.19631^{\{3\}}$	$0.20856^{\{10\}}$	$0.19247^{\{2\}}$	$0.20626^{\{8\}}$
		$\hat{c}$	$0.26983^{\{2\}}$	$0.28426^{\{3\}}$	$0.28535^{\{5\}}$	$0.26181^{\{1\}}$	$0.28455^{\{4\}}$	$0.29368^{\{8\}}$	$0.29037^{\{7\}}$	$0.30396^{\{10\}}$	$0.28623^{\{6\}}$	$0.3009^{\{9\}}$
	D_{abs}		$0.00913^{\{1\}}$	$0.00921^{\{2\}}$	$0.00929^{\{3\}}$	$0.00967^{\{5\}}$	$0.00952^{\{4\}}$	$0.01432^{\{8\}}$	$0.01214^{\{7\}}$	$0.01553^{\{9\}}$	$0.01129^{\{6\}}$	$0.01637^{\{10\}}$
	$D_{\max}$		$0.01541^{\{1\}}$	$0.01551^{\{2\}}$	$0.01575^{\{3\}}$	$0.01612^{\{5\}}$	$0.01603^{\{4\}}$	$0.0229^{\{8\}}$	$0.01976^{\{7\}}$	$0.02478^{\{9\}}$	$0.01855^{\{6\}}$	$0.02594^{\{10\}}$
	ASAE		$0.01115^{\{5\}}$	$0.01044^{\{3\}}$	$0.01017^{\{2\}}$	$0.01106^{\{4\}}$	$0.01007^{\{1\}}$	$0.01602^{\{8\}}$	$0.01472^{\{7\}}$	$0.01755^{\{9\}}$	$0.01368^{\{6\}}$	$0.01798^{\{10\}}$
	$\sum$ Ranks		$30^{\{2\}}$	$54^{\{3.5\}}$	$58^{\{5\}}$	$26^{\{1\}}$	$63^{\{6\}}$	$86^{\{8\}}$	$70^{\{7\}}$	$111^{\{10\}}$	$54^{\{3.5\}}$	$108^{\{9\}}$
400	BIAS	$\hat{a}$	$0.0791^{\{1\}}$	$0.08517^{\{2\}}$	$0.09164^{\{8\}}$	$0.08548^{\{3\}}$	$0.09028^{\{7\}}$	$0.08924^{\{6\}}$	$0.08913^{\{5\}}$	$0.0982^{\{10\}}$	$0.08892^{\{4\}}$	$0.09713^{\{9\}}$
		$\hat{b}$	$0.43126^{\{1\}}$	$0.4693^{\{6\}}$	$0.48754^{\{7\}}$	$0.45947^{\{4\}}$	$0.48803^{\{8\}}$	$0.45464^{\{2\}}$	$0.45824^{\{3\}}$	$0.49509^{\{9\}}$	$0.46354^{\{5\}}$	$0.5102^{\{10\}}$
		$\hat{c}$	$0.36632^{\{1\}}$	$0.38868^{\{3\}}$	$0.40722^{\{6\}}$	$0.3772^{\{2\}}$	$0.39878^{\{4\}}$	$0.41384^{\{8\}}$	$0.41112^{\{7\}}$	$0.4383^{\{10\}}$	$0.40684^{\{5\}}$	$0.43277^{\{9\}}$
	MSE	$\hat{a}$	$0.00945^{\{1\}}$	$0.01078^{\{3\}}$	$0.01225^{\{8\}}$	$0.0107^{\{2\}}$	$0.01199^{\{7\}}$	$0.01169^{\{4\}}$	$0.01187^{\{6\}}$	$0.01388^{\{10\}}$	$0.01171^{\{5\}}$	$0.01356^{\{9\}}$
		$\hat{b}$	$0.35035^{\{1\}}$	$0.41278^{\{7\}}$	$0.44828^{\{10\}}$	$0.37421^{\{4\}}$	$0.44826^{\{9\}}$	$0.37153^{\{3\}}$	$0.36381^{\{2\}}$	$0.41224^{\{6\}}$	$0.3763^{\{5\}}$	$0.4228^{\{8\}}$
		$\hat{c}$	$0.21856^{\{2\}}$	$0.25054^{\{3\}}$	$0.27024^{\{7\}}$	$0.21647^{\{1\}}$	$0.26391^{\{5\}}$	$0.27094^{\{8\}}$	$0.26818^{\{6\}}$	$0.29498^{\{10\}}$	$0.25273^{\{4\}}$	$0.28654^{\{9\}}$
	MRE	$\hat{a}$	$0.31642^{\{1\}}$	$0.34068^{\{2\}}$	$0.36654^{\{8\}}$	$0.34191^{\{3\}}$	$0.36112^{\{7\}}$	$0.35695^{\{6\}}$	$0.35652^{\{5\}}$	$0.39281^{\{10\}}$	$0.35568^{\{4\}}$	$0.3885^{\{9\}}$
		$\hat{b}$	$0.1725^{\{1\}}$	$0.18772^{\{6\}}$	$0.19501^{\{7\}}$	$0.18379^{\{4\}}$	$0.19521^{\{8\}}$	$0.18185^{\{2\}}$	$0.18329^{\{3\}}$	$0.19803^{\{9\}}$	$0.18541^{\{5\}}$	$0.20408^{\{10\}}$
		$\hat{c}$	$0.24421^{\{1\}}$	$0.25912^{\{3\}}$	$0.27148^{\{6\}}$	$0.25147^{\{2\}}$	$0.26585^{\{4\}}$	$0.2759^{\{8\}}$	$0.27408^{\{7\}}$	$0.2922^{\{10\}}$	$0.27123^{\{5\}}$	$0.28851^{\{9\}}$
	D_{abs}		$0.00778^{\{3\}}$	$0.00763^{\{2\}}$	$0.00804^{\{4\}}$	$0.00737^{\{1\}}$	$0.00807^{\{5\}}$	$0.01139^{\{8\}}$	$0.0103^{\{7\}}$	$0.01311^{\{10\}}$	$0.00953^{\{6\}}$	$0.01287^{\{9\}}$
	$D_{\max}$		$0.01312^{\{3\}}$	$0.01302^{\{2\}}$	$0.01382^{\{4\}}$	$0.01264^{\{1\}}$	$0.01387^{\{5\}}$	$0.01851^{\{8\}}$	$0.01698^{\{7\}}$	$0.02126^{\{10\}}$	$0.01582^{\{6\}}$	$0.02085^{\{9\}}$
	ASAE		$0.0088^{\{4\}}$	$0.00832^{\{3\}}$	$0.00816^{\{2\}}$	$0.0089^{\{5\}}$	$0.0081^{\{1\}}$	$0.013^{\{8\}}$	$0.01178^{\{7\}}$	$0.01417^{\{9\}}$	$0.01079^{\{6\}}$	$0.01423^{\{10\}}$
	$\sum$ Ranks		$20^{\{1\}}$	$42^{\{3\}}$	$77^{\{8\}}$	$32^{\{2\}}$	$70^{\{6\}}$	$71^{\{7\}}$	$65^{\{5\}}$	$113^{\{10\}}$	$60^{\{4\}}$	$110^{\{9\}}$

Table 5: Numerical values for some measures for $a = 0.9,\ b = 2.0,\ c = 2.5$ under SRS.

$s^{\bullet}$	Measure	Est.	ML	AD	CRM	MXPS	LS	SPAD	SPALoD	MSSD	MSSLD	MSLND
30	BIAS	$\hat{a}$	$0.33296^{\{8\}}$	$0.30521^{\{2\}}$	$0.33492^{\{10\}}$	$0.29293^{\{1\}}$	$0.32197^{\{6\}}$	$0.30578^{\{3\}}$	$0.31511^{\{5\}}$	$0.33489^{\{9\}}$	$0.30772^{\{4\}}$	$0.33199^{\{7\}}$
		$\hat{b}$	$0.96273^{\{10\}}$	$0.93391^{\{8\}}$	$0.93467^{\{9\}}$	$0.85717^{\{1\}}$	$0.88958^{\{3\}}$	$0.87619^{\{2\}}$	$0.90932^{\{6\}}$	$0.89716^{\{5\}}$	$0.91672^{\{7\}}$	$0.89233^{\{4\}}$
		$\hat{c}$	$0.83031^{\{9\}}$	$0.82868^{\{8\}}$	$0.84684^{\{10\}}$	$0.74341^{\{1\}}$	$0.79766^{\{4\}}$	$0.80998^{\{6\}}$	$0.81675^{\{7\}}$	$0.79168^{\{3\}}$	$0.79023^{\{2\}}$	$0.80844^{\{5\}}$
	MSE	$\hat{a}$	$0.16381^{\{9\}}$	$0.14176^{\{4\}}$	$0.16572^{\{10\}}$	$0.12918^{\{1\}}$	$0.15759^{\{6\}}$	$0.14153^{\{3\}}$	$0.14754^{\{5\}}$	$0.16313^{\{8\}}$	$0.14028^{\{2\}}$	$0.16129^{\{7\}}$
		$\hat{b}$	$1.22389^{\{10\}}$	$1.17133^{\{8\}}$	$1.17562^{\{9\}}$	$1.01136^{\{1\}}$	$1.089^{\{4\}}$	$1.07268^{\{3\}}$	$1.11773^{\{7\}}$	$1.10726^{\{5\}}$	$1.11332^{\{6\}}$	$1.0616^{\{2\}}$
		$\hat{c}$	$0.98648^{\{6\}}$	$1.0021^{\{9\}}$	$1.08379^{\{10\}}$	$0.80509^{\{1\}}$	$0.97028^{\{5\}}$	$0.9896^{\{7\}}$	$0.99763^{\{8\}}$	$0.91988^{\{3\}}$	$0.90183^{\{2\}}$	$0.94296^{\{4\}}$
	MRE	$\hat{a}$	$0.36995^{\{8\}}$	$0.33912^{\{2\}}$	$0.37214^{\{10\}}$	$0.32548^{\{1\}}$	$0.35775^{\{6\}}$	$0.33976^{\{3\}}$	$0.35012^{\{5\}}$	$0.3721^{\{9\}}$	$0.34191^{\{4\}}$	$0.36887^{\{7\}}$
		$\hat{b}$	$0.48136^{\{10\}}$	$0.46696^{\{8\}}$	$0.46733^{\{9\}}$	$0.42858^{\{1\}}$	$0.44479^{\{3\}}$	$0.4381^{\{2\}}$	$0.45466^{\{6\}}$	$0.44858^{\{5\}}$	$0.45836^{\{7\}}$	$0.44616^{\{4\}}$
		$\hat{c}$	$0.33212^{\{9\}}$	$0.33147^{\{8\}}$	$0.33874^{\{10\}}$	$0.29736^{\{1\}}$	$0.31906^{\{4\}}$	$0.32399^{\{6\}}$	$0.3267^{\{7\}}$	$0.31667^{\{3\}}$	$0.31609^{\{2\}}$	$0.32338^{\{5\}}$
	D_{abs}		$0.04136^{\{1\}}$	$0.04191^{\{2\}}$	$0.04513^{\{6\}}$	$0.04264^{\{3\}}$	$0.04294^{\{4\}}$	$0.05009^{\{8\}}$	$0.04746^{\{7\}}$	$0.05283^{\{9\}}$	$0.0437^{\{5\}}$	$0.05439^{\{10\}}$
	$D_{\max}$		$0.06723^{\{3\}}$	$0.06675^{\{2\}}$	$0.07198^{\{6\}}$	$0.06519^{\{1\}}$	$0.0675^{\{4\}}$	$0.0762^{\{8\}}$	$0.07281^{\{7\}}$	$0.07985^{\{9\}}$	$0.06809^{\{5\}}$	$0.08205^{\{10\}}$
	ASAE		$0.03555^{\{5\}}$	$0.03179^{\{3\}}$	$0.03121^{\{2\}}$	$0.03347^{\{4\}}$	$0.03093^{\{1\}}$	$0.04793^{\{9\}}$	$0.04338^{\{7\}}$	$0.04789^{\{8\}}$	$0.0398^{\{6\}}$	$0.04903^{\{10\}}$
	$\sum$ Ranks		$88^{\{9\}}$	$64^{\{5\}}$	$101^{\{10\}}$	$17^{\{1\}}$	$50^{\{2\}}$	$60^{\{4\}}$	$77^{\{8\}}$	$76^{\{7\}}$	$52^{\{3\}}$	$75^{\{6\}}$
75	BIAS	$\hat{a}$	$0.25721^{\{4\}}$	$0.26283^{\{5\}}$	$0.28073^{\{10\}}$	$0.23785^{\{1\}}$	$0.26779^{\{7\}}$	$0.26409^{\{6\}}$	$0.24643^{\{3\}}$	$0.27551^{\{8\}}$	$0.24459^{\{2\}}$	$0.28^{\{9\}}$
		$\hat{b}$	$0.80447^{\{7\}}$	$0.78008^{\{2\}}$	$0.82388^{\{10\}}$	$0.76714^{\{1\}}$	$0.80841^{\{9\}}$	$0.80549^{\{8\}}$	$0.78721^{\{4\}}$	$0.80232^{\{6\}}$	$0.78534^{\{3\}}$	$0.80144^{\{5\}}$
		$\hat{c}$	$0.69435^{\{3\}}$	$0.70002^{\{4\}}$	$0.72313^{\{9\}}$	$0.67072^{\{1\}}$	$0.71599^{\{8\}}$	$0.72543^{\{10\}}$	$0.70574^{\{6\}}$	$0.71433^{\{7\}}$	$0.68145^{\{2\}}$	$0.70412^{\{5\}}$
	MSE	$\hat{a}$	$0.10171^{\{4\}}$	$0.10765^{\{5\}}$	$0.11983^{\{9\}}$	$0.08795^{\{1\}}$	$0.11101^{\{7\}}$	$0.10896^{\{6\}}$	$0.09586^{\{3\}}$	$0.11526^{\{8\}}$	$0.09362^{\{2\}}$	$0.12258^{\{10\}}$
		$\hat{b}$	$0.8978^{\{5\}}$	$0.85931^{\{2\}}$	$0.93568^{\{10\}}$	$0.84029^{\{1\}}$	$0.91523^{\{8\}}$	$0.92666^{\{9\}}$	$0.88059^{\{4\}}$	$0.90379^{\{6\}}$	$0.86018^{\{3\}}$	$0.90859^{\{7\}}$
		$\hat{c}$	$0.73297^{\{3\}}$	$0.77366^{\{5\}}$	$0.80572^{\{8\}}$	$0.71332^{\{2\}}$	$0.81132^{\{9\}}$	$0.82922^{\{10\}}$	$0.77988^{\{7\}}$	$0.77672^{\{6\}}$	$0.70937^{\{1\}}$	$0.74671^{\{4\}}$
	MRE	$\hat{a}$	$0.28579^{\{4\}}$	$0.29204^{\{5\}}$	$0.31192^{\{10\}}$	$0.26427^{\{1\}}$	$0.29755^{\{7\}}$	$0.29343^{\{6\}}$	$0.27382^{\{3\}}$	$0.30613^{\{8\}}$	$0.27176^{\{2\}}$	$0.31111^{\{9\}}$
		$\hat{b}$	$0.40224^{\{7\}}$	$0.39004^{\{2\}}$	$0.41194^{\{10\}}$	$0.38357^{\{1\}}$	$0.40421^{\{9\}}$	$0.40275^{\{8\}}$	$0.39361^{\{4\}}$	$0.40116^{\{6\}}$	$0.39267^{\{3\}}$	$0.40072^{\{5\}}$
		$\hat{c}$	$0.27774^{\{3\}}$	$0.28001^{\{4\}}$	$0.28925^{\{9\}}$	$0.26829^{\{1\}}$	$0.28639^{\{8\}}$	$0.29017^{\{10\}}$	$0.28229^{\{6\}}$	$0.28573^{\{7\}}$	$0.27258^{\{2\}}$	$0.28165^{\{5\}}$
	D_{abs}		$0.02728^{\{1\}}$	$0.02762^{\{2\}}$	$0.02957^{\{5\}}$	$0.02786^{\{3\}}$	$0.02811^{\{4\}}$	$0.03312^{\{8\}}$	$0.03102^{\{7\}}$	$0.03607^{\{9\}}$	$0.03086^{\{6\}}$	$0.03626^{\{10\}}$
	$D_{\max}$		$0.04472^{\{2\}}$	$0.04501^{\{3\}}$	$0.04884^{\{5\}}$	$0.04424^{\{1\}}$	$0.04608^{\{4\}}$	$0.05231^{\{8\}}$	$0.04885^{\{6\}}$	$0.05654^{\{9\}}$	$0.04889^{\{7\}}$	$0.05672^{\{10\}}$
	ASAE		$0.02092^{\{5\}}$	$0.01909^{\{1\}}$	$0.01914^{\{2\}}$	$0.02056^{\{4\}}$	$0.0192^{\{3\}}$	$0.03002^{\{8\}}$	$0.02682^{\{7\}}$	$0.03108^{\{9\}}$	$0.0247^{\{6\}}$	$0.03121^{\{10\}}$
	$\sum$ Ranks		$48^{\{4\}}$	$40^{\{3\}}$	$97^{\{9.5\}}$	$18^{\{1\}}$	$83^{\{6\}}$	$97^{\{9.5\}}$	$60^{\{5\}}$	$89^{\{7.5\}}$	$39^{\{2\}}$	$89^{\{7.5\}}$
150	BIAS	$\hat{a}$	$0.20216^{\{3\}}$	$0.20301^{\{4\}}$	$0.21926^{\{7\}}$	$0.18208^{\{1\}}$	$0.21193^{\{6\}}$	$0.2209^{\{8\}}$	$0.20997^{\{5\}}$	$0.23017^{\{10\}}$	$0.19972^{\{2\}}$	$0.22681^{\{9\}}$
		$\hat{b}$	$0.66883^{\{3\}}$	$0.668^{\{2\}}$	$0.69067^{\{7\}}$	$0.6278^{\{1\}}$	$0.67627^{\{4\}}$	$0.71355^{\{8\}}$	$0.67907^{\{6\}}$	$0.7431^{\{9\}}$	$0.67628^{\{5\}}$	$0.74909^{\{10\}}$
		$\hat{c}$	$0.57775^{\{2\}}$	$0.615^{\{4\}}$	$0.62486^{\{5\}}$	$0.57581^{\{1\}}$	$0.6362^{\{6\}}$	$0.64557^{\{8\}}$	$0.64436^{\{7\}}$	$0.6492^{\{9\}}$	$0.60343^{\{3\}}$	$0.65029^{\{10\}}$
	MSE	$\hat{a}$	$0.06436^{\{3\}}$	$0.06472^{\{4\}}$	$0.07621^{\{7\}}$	$0.05149^{\{1\}}$	$0.07049^{\{6\}}$	$0.07801^{\{8\}}$	$0.06867^{\{5\}}$	$0.08034^{\{10\}}$	$0.06372^{\{2\}}$	$0.07884^{\{9\}}$
		$\hat{b}$	$0.64451^{\{2\}}$	$0.66403^{\{3\}}$	$0.69761^{\{7\}}$	$0.58882^{\{1\}}$	$0.68925^{\{6\}}$	$0.74708^{\{8\}}$	$0.68471^{\{5\}}$	$0.79523^{\{10\}}$	$0.67329^{\{4\}}$	$0.7904^{\{9\}}$
		$\hat{c}$	$0.5309^{\{1\}}$	$0.61625^{\{4\}}$	$0.67404^{\{7\}}$	$0.54039^{\{2\}}$	$0.67985^{\{9\}}$	$0.69521^{\{10\}}$	$0.66778^{\{6\}}$	$0.67519^{\{8\}}$	$0.58428^{\{3\}}$	$0.65539^{\{5\}}$
	MRE	$\hat{a}$	$0.22462^{\{3\}}$	$0.22557^{\{4\}}$	$0.24363^{\{7\}}$	$0.20231^{\{1\}}$	$0.23548^{\{6\}}$	$0.24544^{\{8\}}$	$0.2333^{\{5\}}$	$0.25574^{\{10\}}$	$0.22192^{\{2\}}$	$0.25201^{\{9\}}$
		$\hat{b}$	$0.33442^{\{3\}}$	$0.334^{\{2\}}$	$0.34533^{\{7\}}$	$0.3139^{\{1\}}$	$0.33813^{\{4\}}$	$0.35677^{\{8\}}$	$0.33954^{\{6\}}$	$0.37155^{\{9\}}$	$0.33814^{\{5\}}$	$0.37455^{\{10\}}$
		$\hat{c}$	$0.2311^{\{2\}}$	$0.246^{\{4\}}$	$0.24995^{\{5\}}$	$0.23032^{\{1\}}$	$0.25448^{\{6\}}$	$0.25823^{\{8\}}$	$0.25774^{\{7\}}$	$0.25968^{\{9\}}$	$0.24137^{\{3\}}$	$0.26012^{\{10\}}$
	D_{abs}		$0.02016^{\{3\}}$	$0.01953^{\{2\}}$	$0.02107^{\{5\}}$	$0.01921^{\{1\}}$	$0.02066^{\{4\}}$	$0.02458^{\{8\}}$	$0.02395^{\{7\}}$	$0.02464^{\{9\}}$	$0.02213^{\{6\}}$	$0.02616^{\{10\}}$
	$D_{\max}$		$0.03314^{\{3\}}$	$0.03224^{\{2\}}$	$0.03496^{\{5\}}$	$0.03085^{\{1\}}$	$0.03403^{\{4\}}$	$0.0395^{\{8\}}$	$0.0383^{\{7\}}$	$0.03963^{\{9\}}$	$0.03562^{\{6\}}$	$0.0419^{\{10\}}$
	ASAE		$0.0145^{\{5\}}$	$0.01361^{\{3\}}$	$0.01313^{\{2\}}$	$0.01415^{\{4\}}$	$0.01307^{\{1\}}$	$0.02036^{\{8\}}$	$0.01893^{\{7\}}$	$0.02201^{\{10\}}$	$0.01748^{\{6\}}$	$0.02197^{\{9\}}$
	$\sum$ Ranks		$33^{\{2\}}$	$38^{\{3\}}$	$71^{\{6\}}$	$16^{\{1\}}$	$62^{\{5\}}$	$98^{\{8\}}$	$73^{\{7\}}$	$112^{\{10\}}$	$47^{\{4\}}$	$110^{\{9\}}$
250	BIAS	$\hat{a}$	$0.16136^{\{2\}}$	$0.16577^{\{3\}}$	$0.17241^{\{6\}}$	$0.1566^{\{1\}}$	$0.17353^{\{7\}}$	$0.18442^{\{8\}}$	$0.17037^{\{5\}}$	$0.20431^{\{10\}}$	$0.1665^{\{4\}}$	$0.19787^{\{9\}}$
		$\hat{b}$	$0.58532^{\{2\}}$	$0.59035^{\{3\}}$	$0.57741^{\{1\}}$	$0.59375^{\{4\}}$	$0.59996^{\{5\}}$	$0.64327^{\{8\}}$	$0.6149^{\{7\}}$	$0.68371^{\{10\}}$	$0.60426^{\{6\}}$	$0.64987^{\{9\}}$
		$\hat{c}$	$0.50579^{\{1\}}$	$0.55572^{\{5\}}$	$0.55765^{\{6\}}$	$0.51789^{\{2\}}$	$0.54578^{\{3\}}$	$0.59917^{\{10\}}$	$0.5704^{\{7\}}$	$0.59899^{\{9\}}$	$0.54608^{\{4\}}$	$0.57815^{\{8\}}$
	MSE	$\hat{a}$	$0.04019^{\{2\}}$	$0.04274^{\{3\}}$	$0.04836^{\{7\}}$	$0.03777^{\{1\}}$	$0.04594^{\{6\}}$	$0.05392^{\{8\}}$	$0.04471^{\{5\}}$	$0.06515^{\{10\}}$	$0.0429^{\{4\}}$	$0.06116^{\{9\}}$
		$\hat{b}$	$0.51077^{\{1\}}$	$0.55164^{\{4\}}$	$0.5218^{\{2\}}$	$0.54397^{\{3\}}$	$0.56128^{\{6\}}$	$0.62835^{\{8\}}$	$0.584^{\{7\}}$	$0.6837^{\{10\}}$	$0.55557^{\{5\}}$	$0.63817^{\{9\}}$
		$\hat{c}$	$0.4105^{\{1\}}$	$0.52375^{\{5\}}$	$0.57128^{\{8\}}$	$0.43845^{\{2\}}$	$0.5161^{\{4\}}$	$0.61752^{\{10\}}$	$0.54339^{\{6\}}$	$0.57537^{\{9\}}$	$0.49633^{\{3\}}$	$0.54577^{\{7\}}$
	MRE	$\hat{a}$	$0.17929^{\{2\}}$	$0.18419^{\{3\}}$	$0.19156^{\{6\}}$	$0.174^{\{1\}}$	$0.19281^{\{7\}}$	$0.20492^{\{8\}}$	$0.1893^{\{5\}}$	$0.22701^{\{10\}}$	$0.185^{\{4\}}$	$0.21986^{\{9\}}$
		$\hat{b}$	$0.29266^{\{2\}}$	$0.29517^{\{3\}}$	$0.28871^{\{1\}}$	$0.29687^{\{4\}}$	$0.29998^{\{5\}}$	$0.32164^{\{8\}}$	$0.30745^{\{7\}}$	$0.34185^{\{10\}}$	$0.30213^{\{6\}}$	$0.32493^{\{9\}}$
		$\hat{c}$	$0.20232^{\{1\}}$	$0.22229^{\{5\}}$	$0.22306^{\{6\}}$	$0.20716^{\{2\}}$	$0.21831^{\{3\}}$	$0.23967^{\{10\}}$	$0.22816^{\{7\}}$	$0.2396^{\{9\}}$	$0.21843^{\{4\}}$	$0.23126^{\{8\}}$
	D_{abs}		$0.01555^{\{1\}}$	$0.01581^{\{3\}}$	$0.01615^{\{4\}}$	$0.01575^{\{2\}}$	$0.01678^{\{5\}}$	$0.01883^{\{8\}}$	$0.01712^{\{6\}}$	$0.01988^{\{9\}}$	$0.01759^{\{7\}}$	$0.02071^{\{10\}}$
	$D_{\max}$		$0.02564^{\{2\}}$	$0.02605^{\{3\}}$	$0.02676^{\{4\}}$	$0.02554^{\{1\}}$	$0.02764^{\{5\}}$	$0.0305^{\{8\}}$	$0.02782^{\{6\}}$	$0.0324^{\{9\}}$	$0.02845^{\{7\}}$	$0.03341^{\{10\}}$
	ASAE		$0.01104^{\{4\}}$	$0.01038^{\{3\}}$	$0.01012^{\{2\}}$	$0.01107^{\{5\}}$	$0.01007^{\{1\}}$	$0.01583^{\{8\}}$	$0.01442^{\{7\}}$	$0.01761^{\{10\}}$	$0.01327^{\{6\}}$	$0.01736^{\{9\}}$
	$\sum$ Ranks		$21^{\{1\}}$	$43^{\{3\}}$	$53^{\{4\}}$	$28^{\{2\}}$	$57^{\{5\}}$	$102^{\{8\}}$	$75^{\{7\}}$	$115^{\{10\}}$	$60^{\{6\}}$	$106^{\{9\}}$
400	BIAS	$\hat{a}$	$0.13087^{\{1.5\}}$	$0.13638^{\{3\}}$	$0.1519^{\{7\}}$	$0.13087^{\{1.5\}}$	$0.14259^{\{4\}}$	$0.15768^{\{8\}}$	$0.1438^{\{5\}}$	$0.1722^{\{10\}}$	$0.14478^{\{6\}}$	$0.16934^{\{9\}}$
		$\hat{b}$	$0.489^{\{1\}}$	$0.50354^{\{2\}}$	$0.53243^{\{5\}}$	$0.50933^{\{4\}}$	$0.50759^{\{3\}}$	$0.5692^{\{8\}}$	$0.55809^{\{7\}}$	$0.6261^{\{10\}}$	$0.54536^{\{6\}}$	$0.61593^{\{9\}}$
		$\hat{c}$	$0.4321^{\{1\}}$	$0.46831^{\{3\}}$	$0.49154^{\{6\}}$	$0.43703^{\{2\}}$	$0.47141^{\{4\}}$	$0.52045^{\{8\}}$	$0.49328^{\{7\}}$	$0.55148^{\{10\}}$	$0.47746^{\{5\}}$	$0.5465^{\{9\}}$
	MSE	$\hat{a}$	$0.02696^{\{2\}}$	$0.0292^{\{3\}}$	$0.03649^{\{7\}}$	$0.02689^{\{1\}}$	$0.03252^{\{4\}}$	$0.03981^{\{8\}}$	$0.03254^{\{5\}}$	$0.04604^{\{10\}}$	$0.03277^{\{6\}}$	$0.04553^{\{9\}}$
		$\hat{b}$	$0.39046^{\{1\}}$	$0.42857^{\{2\}}$	$0.46864^{\{5\}}$	$0.43508^{\{3\}}$	$0.45322^{\{4\}}$	$0.51766^{\{8\}}$	$0.50875^{\{7\}}$	$0.60105^{\{10\}}$	$0.47531^{\{6\}}$	$0.5678^{\{9\}}$
		$\hat{c}$	$0.32272^{\{1\}}$	$0.40712^{\{4\}}$	$0.42844^{\{7\}}$	$0.33868^{\{2\}}$	$0.40772^{\{5\}}$	$0.47245^{\{8\}}$	$0.41518^{\{6\}}$	$0.50595^{\{10\}}$	$0.3937^{\{3\}}$	$0.49737^{\{9\}}$
	MRE	$\hat{a}$	$0.14541^{\{1.5\}}$	$0.15153^{\{3\}}$	$0.16878^{\{7\}}$	$0.14541^{\{1.5\}}$	$0.15843^{\{4\}}$	$0.1752^{\{8\}}$	$0.15978^{\{5\}}$	$0.19133^{\{10\}}$	$0.16087^{\{6\}}$	$0.18816^{\{9\}}$
		$\hat{b}$	$0.2445^{\{1\}}$	$0.25177^{\{2\}}$	$0.26621^{\{5\}}$	$0.25466^{\{4\}}$	$0.2538^{\{3\}}$	$0.2846^{\{8\}}$	$0.27904^{\{7\}}$	$0.31305^{\{10\}}$	$0.27268^{\{6\}}$	$0.30796^{\{9\}}$
		$\hat{c}$	$0.17284^{\{1\}}$	$0.18732^{\{3\}}$	$0.19661^{\{6\}}$	$0.17481^{\{2\}}$	$0.18856^{\{4\}}$	$0.20818^{\{8\}}$	$0.19731^{\{7\}}$	$0.22059^{\{10\}}$	$0.19098^{\{5\}}$	$0.2186^{\{9\}}$
	D_{abs}		$0.01182^{\{1\}}$	$0.01226^{\{2\}}$	$0.01318^{\{5\}}$	$0.01241^{\{3\}}$	$0.01309^{\{4\}}$	$0.01557^{\{8\}}$	$0.01438^{\{7\}}$	$0.01653^{\{9\}}$	$0.01366^{\{6\}}$	$0.01654^{\{10\}}$
	$D_{\max}$		$0.01948^{\{1\}}$	$0.02031^{\{3\}}$	$0.02206^{\{5\}}$	$0.02028^{\{2\}}$	$0.02162^{\{4\}}$	$0.02543^{\{8\}}$	$0.02339^{\{7\}}$	$0.02694^{\{9\}}$	$0.02236^{\{6\}}$	$0.02698^{\{10\}}$
	ASAE		$0.00856^{\{4\}}$	$0.00804^{\{3\}}$	$0.00798^{\{2\}}$	$0.00867^{\{5\}}$	$0.00797^{\{1\}}$	$0.01273^{\{8\}}$	$0.01128^{\{7\}}$	$0.01385^{\{10\}}$	$0.01052^{\{6\}}$	$0.01377^{\{9\}}$
	$\sum$ Ranks		$17^{\{1\}}$	$33^{\{3\}}$	$67^{\{5.5\}}$	$31^{\{2\}}$	$44^{\{4\}}$	$96^{\{8\}}$	$77^{\{7\}}$	$118^{\{10\}}$	$67^{\{5.5\}}$	$110^{\{9\}}$

Table 6: Numerical values for some measures for $a = 0.9,\ b = 2.0,\ c = 2.5$ under RSS.

$s^{\bullet}$	Measure	Est.	ML	AD	CRM	MXPS	LS	SPAD	SPALoD	MSSD	MSSLD	MSLND
30	BIAS	$\hat{a}$	0.28896$^{\{3\}}$	0.28878$^{\{2\}}$	0.30452$^{\{9\}}$	0.27672$^{\{1\}}$	0.30152$^{\{6\}}$	0.30195$^{\{7\}}$	0.29566$^{\{4\}}$	0.30573$^{\{10\}}$	0.29981$^{\{5\}}$	0.30353$^{\{8\}}$
		$\hat{b}$	0.84356$^{\{5\}}$	0.84079$^{\{4\}}$	0.87433$^{\{10\}}$	0.80975$^{\{1\}}$	0.84558$^{\{6\}}$	0.84834$^{\{7\}}$	0.85965$^{\{9\}}$	0.83739$^{\{2\}}$	0.85922$^{\{8\}}$	0.84038$^{\{3\}}$
		$\hat{c}$	0.767$^{\{8\}}$	0.77612$^{\{9\}}$	0.75898$^{\{7\}}$	0.7012$^{\{1\}}$	0.72018$^{\{4\}}$	0.78011$^{\{10\}}$	0.74329$^{\{6\}}$	0.74098$^{\{5\}}$	0.71724$^{\{3\}}$	0.71221$^{\{2\}}$
	MSE	$\hat{a}$	0.12809$^{\{2\}}$	0.12878$^{\{3\}}$	0.14037$^{\{10\}}$	0.1158$^{\{1\}}$	0.13776$^{\{8\}}$	0.13696$^{\{7\}}$	0.13201$^{\{4\}}$	0.13419$^{\{5\}}$	0.1363$^{\{6\}}$	0.13808$^{\{9\}}$
		$\hat{b}$	0.986$^{\{5\}}$	0.95584$^{\{2\}}$	1.04861$^{\{10\}}$	0.91171$^{\{1\}}$	0.99271$^{\{6\}}$	1.01814$^{\{8\}}$	1.02401$^{\{9\}}$	0.96183$^{\{3\}}$	1.0119$^{\{7\}}$	0.98257$^{\{4\}}$
		$\hat{c}$	0.90152$^{\{9\}}$	0.89424$^{\{8\}}$	0.8751$^{\{7\}}$	0.77921$^{\{2\}}$	0.81477$^{\{4\}}$	0.94492$^{\{10\}}$	0.86935$^{\{6\}}$	0.84408$^{\{5\}}$	0.7952$^{\{3\}}$	0.77466$^{\{1\}}$
	MRE	$\hat{a}$	0.32107$^{\{3\}}$	0.32086$^{\{2\}}$	0.33836$^{\{9\}}$	0.30746$^{\{1\}}$	0.33502$^{\{6\}}$	0.3355$^{\{7\}}$	0.32851$^{\{4\}}$	0.3397$^{\{10\}}$	0.33312$^{\{5\}}$	0.33726$^{\{8\}}$
		$\hat{b}$	0.42178$^{\{5\}}$	0.4204$^{\{4\}}$	0.43717$^{\{10\}}$	0.40488$^{\{1\}}$	0.42279$^{\{6\}}$	0.42417$^{\{7\}}$	0.42983$^{\{9\}}$	0.41869$^{\{2\}}$	0.42961$^{\{8\}}$	0.42019$^{\{3\}}$
		$\hat{c}$	0.3068$^{\{8\}}$	0.31045$^{\{9\}}$	0.30359$^{\{7\}}$	0.28048$^{\{1\}}$	0.28807$^{\{4\}}$	0.31204$^{\{10\}}$	0.29731$^{\{6\}}$	0.29639$^{\{5\}}$	0.2869$^{\{3\}}$	0.28488$^{\{2\}}$
	D_{abs}		0.02714$^{\{1\}}$	0.02761$^{\{3\}}$	0.02822$^{\{4\}}$	0.02717$^{\{2\}}$	0.03012$^{\{5\}}$	0.04052$^{\{8\}}$	0.03542$^{\{7\}}$	0.04182$^{\{9\}}$	0.03261$^{\{6\}}$	0.04202$^{\{10\}}$
	$D_{\max}$		0.04496$^{\{2\}}$	0.04498$^{\{3\}}$	0.04692$^{\{4\}}$	0.04342$^{\{1\}}$	0.04855$^{\{5\}}$	0.06281$^{\{8\}}$	0.05565$^{\{7\}}$	0.064$^{\{10\}}$	0.05189$^{\{6\}}$	0.06399$^{\{9\}}$
	ASAE		0.03454$^{\{5\}}$	0.03159$^{\{3\}}$	0.03102$^{\{2\}}$	0.03334$^{\{4\}}$	0.0307$^{\{1\}}$	0.04777$^{\{9\}}$	0.04324$^{\{7\}}$	0.04729$^{\{8\}}$	0.03926$^{\{6\}}$	0.04842$^{\{10\}}$
	$\sum$ Ranks		56$^{\{3\}}$	52$^{\{2\}}$	89$^{\{9\}}$	17$^{\{1\}}$	61$^{\{4\}}$	98$^{\{10\}}$	78$^{\{8\}}$	74$^{\{7\}}$	66$^{\{5\}}$	69$^{\{6\}}$
75	BIAS	$\hat{a}$	0.21307$^{\{2\}}$	0.22544$^{\{3\}}$	0.23403$^{\{5\}}$	0.20011$^{\{1\}}$	0.23182$^{\{4\}}$	0.25119$^{\{8\}}$	0.23723$^{\{7\}}$	0.25845$^{\{9\}}$	0.2344$^{\{6\}}$	0.26223$^{\{10\}}$
		$\hat{b}$	0.67373$^{\{2\}}$	0.71052$^{\{3\}}$	0.72633$^{\{4\}}$	0.65661$^{\{1\}}$	0.73163$^{\{5\}}$	0.77437$^{\{8\}}$	0.7498$^{\{7\}}$	0.7756$^{\{9\}}$	0.74171$^{\{6\}}$	0.7832$^{\{10\}}$
		$\hat{c}$	0.60804$^{\{2\}}$	0.63979$^{\{6\}}$	0.62301$^{\{3\}}$	0.59663$^{\{1\}}$	0.63176$^{\{5\}}$	0.70824$^{\{10\}}$	0.66849$^{\{7\}}$	0.6742$^{\{9\}}$	0.62693$^{\{4\}}$	0.67151$^{\{8\}}$
	MSE	$\hat{a}$	0.07162$^{\{2\}}$	0.07836$^{\{3\}}$	0.08877$^{\{7\}}$	0.06208$^{\{1\}}$	0.08563$^{\{4\}}$	0.09529$^{\{8\}}$	0.08719$^{\{5\}}$	0.10077$^{\{9\}}$	0.08722$^{\{6\}}$	0.10634$^{\{10\}}$
		$\hat{b}$	0.66212$^{\{2\}}$	0.73024$^{\{3\}}$	0.76496$^{\{4\}}$	0.64508$^{\{1\}}$	0.78379$^{\{5\}}$	0.84451$^{\{8\}}$	0.80494$^{\{7\}}$	0.84946$^{\{9\}}$	0.79224$^{\{6\}}$	0.8646$^{\{10\}}$
		$\hat{c}$	0.58813$^{\{1\}}$	0.67637$^{\{6\}}$	0.63408$^{\{4\}}$	0.5883$^{\{2\}}$	0.66028$^{\{5\}}$	0.80013$^{\{10\}}$	0.71718$^{\{8\}}$	0.70328$^{\{7\}}$	0.63266$^{\{3\}}$	0.71866$^{\{9\}}$
	MRE	$\hat{a}$	0.23674$^{\{2\}}$	0.25049$^{\{3\}}$	0.26003$^{\{5\}}$	0.22235$^{\{1\}}$	0.25758$^{\{4\}}$	0.2791$^{\{8\}}$	0.26358$^{\{7\}}$	0.28717$^{\{9\}}$	0.26045$^{\{6\}}$	0.29137$^{\{10\}}$
		$\hat{b}$	0.33686$^{\{2\}}$	0.35526$^{\{3\}}$	0.36316$^{\{4\}}$	0.32831$^{\{1\}}$	0.36582$^{\{5\}}$	0.38719$^{\{8\}}$	0.3749$^{\{7\}}$	0.3878$^{\{9\}}$	0.37086$^{\{6\}}$	0.3916$^{\{10\}}$
		$\hat{c}$	0.24322$^{\{2\}}$	0.25591$^{\{6\}}$	0.2492$^{\{3\}}$	0.23865$^{\{1\}}$	0.2527$^{\{5\}}$	0.2833$^{\{10\}}$	0.26739$^{\{7\}}$	0.26968$^{\{9\}}$	0.25077$^{\{4\}}$	0.2686$^{\{8\}}$
	D_{abs}		0.01729$^{\{2\}}$	0.01781$^{\{3\}}$	0.01877$^{\{5\}}$	0.01676$^{\{1\}}$	0.01875$^{\{4\}}$	0.02634$^{\{8\}}$	0.02389$^{\{7\}}$	0.02841$^{\{9\}}$	0.02146$^{\{6\}}$	0.02909$^{\{10\}}$
	$D_{\max}$		0.02905$^{\{2\}}$	0.02985$^{\{3\}}$	0.0316$^{\{5\}}$	0.02753$^{\{1\}}$	0.03122$^{\{4\}}$	0.04245$^{\{8\}}$	0.03872$^{\{7\}}$	0.04524$^{\{9\}}$	0.03513$^{\{6\}}$	0.04622$^{\{10\}}$
	ASAE		0.02062$^{\{5\}}$	0.01891$^{\{3\}}$	0.01879$^{\{2\}}$	0.01991$^{\{4\}}$	0.01868$^{\{1\}}$	0.02885$^{\{8\}}$	0.02638$^{\{7\}}$	0.03041$^{\{9\}}$	0.02426$^{\{6\}}$	0.0307$^{\{10\}}$
	$\sum$ Ranks		26$^{\{2\}}$	45$^{\{3\}}$	51$^{\{4.5\}}$	16$^{\{1\}}$	51$^{\{4.5\}}$	102$^{\{8\}}$	83$^{\{7\}}$	106$^{\{9\}}$	65$^{\{6\}}$	115$^{\{10\}}$
150	BIAS	$\hat{a}$	0.16593$^{\{2\}}$	0.17191$^{\{3\}}$	0.19037$^{\{5\}}$	0.16156$^{\{1\}}$	0.19841$^{\{8\}}$	0.19753$^{\{7\}}$	0.19099$^{\{6\}}$	0.21424$^{\{9\}}$	0.17575$^{\{4\}}$	0.216$^{\{10\}}$
		$\hat{b}$	0.57515$^{\{1\}}$	0.59888$^{\{3\}}$	0.64861$^{\{8\}}$	0.57966$^{\{2\}}$	0.63755$^{\{5\}}$	0.6443$^{\{6\}}$	0.64618$^{\{7\}}$	0.69423$^{\{9\}}$	0.63074$^{\{4\}}$	0.72175$^{\{10\}}$
		$\hat{c}$	0.54673$^{\{2\}}$	0.54876$^{\{4\}}$	0.57186$^{\{5\}}$	0.52471$^{\{1\}}$	0.60395$^{\{7\}}$	0.59858$^{\{6\}}$	0.60817$^{\{8\}}$	0.61606$^{\{9\}}$	0.54786$^{\{3\}}$	0.62791$^{\{10\}}$
	MSE	$\hat{a}$	0.0414$^{\{2\}}$	0.04595$^{\{3\}}$	0.05637$^{\{6\}}$	0.04055$^{\{1\}}$	0.06115$^{\{7\}}$	0.06254$^{\{8\}}$	0.05494$^{\{5\}}$	0.07167$^{\{9\}}$	0.04835$^{\{4\}}$	0.07234$^{\{10\}}$
		$\hat{b}$	0.50799$^{\{1\}}$	0.57092$^{\{3\}}$	0.64068$^{\{7\}}$	0.53197$^{\{2\}}$	0.63839$^{\{6\}}$	0.64142$^{\{8\}}$	0.62739$^{\{5\}}$	0.6986$^{\{9\}}$	0.60272$^{\{4\}}$	0.73589$^{\{10\}}$
		$\hat{c}$	0.49394$^{\{2\}}$	0.53734$^{\{4\}}$	0.54904$^{\{5\}}$	0.47214$^{\{1\}}$	0.65284$^{\{10\}}$	0.62391$^{\{7\}}$	0.62269$^{\{6\}}$	0.62578$^{\{8\}}$	0.4964$^{\{3\}}$	0.63129$^{\{9\}}$
	MRE	$\hat{a}$	0.18436$^{\{2\}}$	0.19101$^{\{3\}}$	0.21152$^{\{5\}}$	0.17951$^{\{1\}}$	0.22046$^{\{8\}}$	0.21947$^{\{7\}}$	0.21222$^{\{6\}}$	0.23804$^{\{9\}}$	0.19527$^{\{4\}}$	0.24$^{\{10\}}$
		$\hat{b}$	0.28758$^{\{1\}}$	0.29944$^{\{3\}}$	0.3243$^{\{8\}}$	0.28983$^{\{2\}}$	0.31877$^{\{5\}}$	0.32215$^{\{6\}}$	0.32309$^{\{7\}}$	0.34712$^{\{9\}}$	0.31537$^{\{4\}}$	0.36088$^{\{10\}}$
		$\hat{c}$	0.21869$^{\{2\}}$	0.21951$^{\{4\}}$	0.22874$^{\{5\}}$	0.20988$^{\{1\}}$	0.24158$^{\{7\}}$	0.23943$^{\{6\}}$	0.24327$^{\{8\}}$	0.24642$^{\{9\}}$	0.21914$^{\{3\}}$	0.25116$^{\{10\}}$
	D_{abs}		0.0129$^{\{3\}}$	0.01281$^{\{2\}}$	0.01369$^{\{5\}}$	0.0124$^{\{1\}}$	0.01337$^{\{4\}}$	0.01916$^{\{8\}}$	0.01682$^{\{7\}}$	0.02095$^{\{10\}}$	0.01517$^{\{6\}}$	0.0207$^{\{9\}}$
	$D_{\max}$		0.02181$^{\{3\}}$	0.0216$^{\{2\}}$	0.02341$^{\{5\}}$	0.0207$^{\{1\}}$	0.02301$^{\{4\}}$	0.03113$^{\{8\}}$	0.02776$^{\{7\}}$	0.034$^{\{10\}}$	0.02508$^{\{6\}}$	0.03375$^{\{9\}}$
	ASAE		0.01381$^{\{5\}}$	0.01338$^{\{3\}}$	0.01309$^{\{2\}}$	0.01361$^{\{4\}}$	0.01302$^{\{1\}}$	0.02021$^{\{8\}}$	0.01862$^{\{7\}}$	0.02166$^{\{9\}}$	0.01676$^{\{6\}}$	0.02179$^{\{10\}}$
	$\sum$ Ranks		26$^{\{2\}}$	37$^{\{3\}}$	66$^{\{5\}}$	18$^{\{1\}}$	72$^{\{6\}}$	85$^{\{8\}}$	79$^{\{7\}}$	109$^{\{9\}}$	51$^{\{4\}}$	117$^{\{10\}}$
250	BIAS	$\hat{a}$	0.13087$^{\{1\}}$	0.14752$^{\{3\}}$	0.16646$^{\{8\}}$	0.14446$^{\{2\}}$	0.15875$^{\{5\}}$	0.16524$^{\{7\}}$	0.16198$^{\{6\}}$	0.18795$^{\{10\}}$	0.15402$^{\{4\}}$	0.18491$^{\{9\}}$
		$\hat{b}$	0.49238$^{\{1\}}$	0.53815$^{\{2\}}$	0.54727$^{\{4\}}$	0.54369$^{\{3\}}$	0.56017$^{\{6\}}$	0.59887$^{\{8\}}$	0.58553$^{\{7\}}$	0.64417$^{\{9\}}$	0.55903$^{\{5\}}$	0.64975$^{\{10\}}$
		$\hat{c}$	0.4137$^{\{1\}}$	0.50402$^{\{4\}}$	0.54587$^{\{8\}}$	0.47221$^{\{2\}}$	0.51031$^{\{5\}}$	0.52186$^{\{6\}}$	0.53367$^{\{7\}}$	0.57497$^{\{10\}}$	0.50363$^{\{3\}}$	0.56072$^{\{9\}}$
	MSE	$\hat{a}$	0.02646$^{\{1\}}$	0.03381$^{\{3\}}$	0.04234$^{\{7\}}$	0.03276$^{\{2\}}$	0.03957$^{\{5\}}$	0.04296$^{\{8\}}$	0.04167$^{\{6\}}$	0.05506$^{\{10\}}$	0.03629$^{\{4\}}$	0.05397$^{\{9\}}$
		$\hat{b}$	0.3996$^{\{1\}}$	0.48247$^{\{4\}}$	0.47823$^{\{2\}}$	0.48187$^{\{3\}}$	0.52557$^{\{6\}}$	0.55358$^{\{8\}}$	0.54448$^{\{7\}}$	0.62734$^{\{9\}}$	0.49702$^{\{5\}}$	0.63023$^{\{10\}}$
		$\hat{c}$	0.30773$^{\{1\}}$	0.45333$^{\{4\}}$	0.53129$^{\{8\}}$	0.39996$^{\{2\}}$	0.49535$^{\{6\}}$	0.47163$^{\{5\}}$	0.51551$^{\{7\}}$	0.55789$^{\{10\}}$	0.43413$^{\{3\}}$	0.53316$^{\{9\}}$
	MRE	$\hat{a}$	0.14542$^{\{1\}}$	0.16391$^{\{3\}}$	0.18495$^{\{8\}}$	0.16051$^{\{2\}}$	0.17639$^{\{5\}}$	0.1836$^{\{7\}}$	0.17997$^{\{6\}}$	0.20884$^{\{10\}}$	0.17113$^{\{4\}}$	0.20546$^{\{9\}}$
		$\hat{b}$	0.24619$^{\{1\}}$	0.26908$^{\{2\}}$	0.27364$^{\{4\}}$	0.27184$^{\{3\}}$	0.28008$^{\{6\}}$	0.29944$^{\{8\}}$	0.29276$^{\{7\}}$	0.32208$^{\{9\}}$	0.27951$^{\{5\}}$	0.32487$^{\{10\}}$
		$\hat{c}$	0.16548$^{\{1\}}$	0.20161$^{\{4\}}$	0.21835$^{\{8\}}$	0.18888$^{\{2\}}$	0.20412$^{\{5\}}$	0.20874$^{\{6\}}$	0.21347$^{\{7\}}$	0.22999$^{\{10\}}$	0.20145$^{\{3\}}$	0.22429$^{\{9\}}$
	D_{abs}		0.00988$^{\{2\}}$	0.01029$^{\{3\}}$	0.0108$^{\{5\}}$	0.00986$^{\{1\}}$	0.01031$^{\{4\}}$	0.01534$^{\{8\}}$	0.01395$^{\{7\}}$	0.01696$^{\{10\}}$	0.01252$^{\{6\}}$	0.0165$^{\{9\}}$
	$D_{\max}$		0.01668$^{\{1\}}$	0.01763$^{\{3\}}$	0.01878$^{\{5\}}$	0.01685$^{\{2\}}$	0.01783$^{\{4\}}$	0.02516$^{\{8\}}$	0.02302$^{\{7\}}$	0.02784$^{\{10\}}$	0.02092$^{\{6\}}$	0.02712$^{\{9\}}$
	ASAE		0.01074$^{\{4\}}$	0.01017$^{\{3\}}$	0.00999$^{\{2\}}$	0.01078$^{\{5\}}$	0.00997$^{\{1\}}$	0.0157$^{\{8\}}$	0.0144$^{\{7\}}$	0.01697$^{\{10\}}$	0.01314$^{\{6\}}$	0.01679$^{\{9\}}$
	$\sum$ Ranks		16$^{\{1\}}$	38$^{\{3\}}$	69$^{\{6\}}$	29$^{\{2\}}$	58$^{\{5\}}$	87$^{\{8\}}$	81$^{\{7\}}$	117$^{\{10\}}$	54$^{\{4\}}$	111$^{\{9\}}$
400	BIAS	$\hat{a}$	0.10267$^{\{1\}}$	0.12042$^{\{2\}}$	0.13221$^{\{5\}}$	0.12262$^{\{3\}}$	0.1278$^{\{4\}}$	0.15199$^{\{8\}}$	0.13815$^{\{7\}}$	0.1626$^{\{10\}}$	0.1332$^{\{6\}}$	0.1535$^{\{9\}}$
		$\hat{b}$	0.40333$^{\{1\}}$	0.43574$^{\{2\}}$	0.47907$^{\{5\}}$	0.47551$^{\{4\}}$	0.45058$^{\{3\}}$	0.55539$^{\{8\}}$	0.53598$^{\{7\}}$	0.60106$^{\{10\}}$	0.49636$^{\{6\}}$	0.57185$^{\{9\}}$
		$\hat{c}$	0.34806$^{\{1\}}$	0.4256$^{\{2\}}$	0.4553$^{\{6\}}$	0.42827$^{\{3\}}$	0.45428$^{\{4\}}$	0.4932$^{\{8\}}$	0.47576$^{\{7\}}$	0.51225$^{\{10\}}$	0.45468$^{\{5\}}$	0.4963$^{\{9\}}$
	MSE	$\hat{a}$	0.0174$^{\{1\}}$	0.02235$^{\{2\}}$	0.0264$^{\{5\}}$	0.02389$^{\{3\}}$	0.02567$^{\{4\}}$	0.03549$^{\{8\}}$	0.03008$^{\{7\}}$	0.04042$^{\{10\}}$	0.0279$^{\{6\}}$	0.03761$^{\{9\}}$
		$\hat{b}$	0.30107$^{\{1\}}$	0.3404$^{\{2\}}$	0.40575$^{\{5\}}$	0.40055$^{\{4\}}$	0.36515$^{\{3\}}$	0.50382$^{\{8\}}$	0.47798$^{\{7\}}$	0.56317$^{\{10\}}$	0.41579$^{\{6\}}$	0.52759$^{\{9\}}$
		$\hat{c}$	0.23499$^{\{1\}}$	0.34467$^{\{3\}}$	0.3789$^{\{6\}}$	0.32738$^{\{2\}}$	0.37625$^{\{5\}}$	0.42359$^{\{9\}}$	0.38764$^{\{7\}}$	0.44284$^{\{10\}}$	0.36397$^{\{4\}}$	0.42143$^{\{8\}}$
	MRE	$\hat{a}$	0.11408$^{\{1\}}$	0.1338$^{\{2\}}$	0.1469$^{\{5\}}$	0.13625$^{\{3\}}$	0.142$^{\{4\}}$	0.16887$^{\{8\}}$	0.1535$^{\{7\}}$	0.18067$^{\{10\}}$	0.148$^{\{6\}}$	0.17055$^{\{9\}}$
		$\hat{b}$	0.20167$^{\{1\}}$	0.21787$^{\{2\}}$	0.23954$^{\{5\}}$	0.23776$^{\{4\}}$	0.22529$^{\{3\}}$	0.2777$^{\{8\}}$	0.26799$^{\{7\}}$	0.30053$^{\{10\}}$	0.24818$^{\{6\}}$	0.28592$^{\{9\}}$
		$\hat{c}$	0.13922$^{\{1\}}$	0.17024$^{\{2\}}$	0.18212$^{\{6\}}$	0.17131$^{\{3\}}$	0.18171$^{\{4\}}$	0.19728$^{\{8\}}$	0.1903$^{\{7\}}$	0.2049$^{\{10\}}$	0.18187$^{\{5\}}$	0.19852$^{\{9\}}$
	D_{abs}		0.00751$^{\{1\}}$	0.00804$^{\{3\}}$	0.00838$^{\{5\}}$	0.00793$^{\{2\}}$	0.00811$^{\{4\}}$	0.01171$^{\{8\}}$	0.01078$^{\{7\}}$	0.01372$^{\{10\}}$	0.00999$^{\{6\}}$	0.01337$^{\{9\}}$
	$D_{\max}$		0.0127$^{\{1\}}$	0.0138$^{\{3\}}$	0.01465$^{\{5\}}$	0.01358$^{\{2\}}$	0.0141$^{\{4\}}$	0.01963$^{\{8\}}$	0.01807$^{\{7\}}$	0.02277$^{\{10\}}$	0.01685$^{\{6\}}$	0.02208$^{\{9\}}$
	ASAE		0.00842$^{\{5\}}$	0.00794$^{\{3\}}$	0.00789$^{\{2\}}$	0.0084$^{\{4\}}$	0.00783$^{\{1\}}$	0.01217$^{\{8\}}$	0.01118$^{\{7\}}$	0.01366$^{\{10\}}$	0.01031$^{\{6\}}$	0.01357$^{\{9\}}$
	$\sum$ Ranks		16$^{\{1\}}$	28$^{\{2\}}$	60$^{\{5\}}$	37$^{\{3\}}$	43$^{\{4\}}$	97$^{\{8\}}$	84$^{\{7\}}$	120$^{\{10\}}$	68$^{\{6\}}$	107$^{\{9\}}$

Table 7: Numerical values for some measures for $a = 2.0,\ b = 1.0,\ c = 0.5$ under SRS.

$s^{\bullet}$	Measure	Est.	ML	AD	CRM	MXPS	LS	SPAD	SPALoD	MSSD	MSSLD	MSLND
30	BIAS	$\hat{a}$	0.49861$^{\{4\}}$	0.60312$^{\{10\}}$	0.57383$^{\{9\}}$	0.47502$^{\{2\}}$	0.54976$^{\{8\}}$	0.45893$^{\{1\}}$	0.48737$^{\{3\}}$	0.50399$^{\{5\}}$	0.51607$^{\{7\}}$	0.50876$^{\{6\}}$
		$\hat{b}$	0.46052$^{\{4\}}$	0.53556$^{\{10\}}$	0.53031$^{\{9\}}$	0.44891$^{\{3\}}$	0.51092$^{\{8\}}$	0.42551$^{\{1\}}$	0.44457$^{\{2\}}$	0.4986$^{\{7\}}$	0.49684$^{\{6\}}$	0.49644$^{\{5\}}$
		$\hat{c}$	0.25892$^{\{3\}}$	0.254$^{\{2\}}$	0.25282$^{\{1\}}$	0.27381$^{\{10\}}$	0.26194$^{\{5\}}$	0.26682$^{\{7\}}$	0.26$^{\{4\}}$	0.27133$^{\{8\}}$	0.26482$^{\{6\}}$	0.27224$^{\{9\}}$
	MSE	$\hat{a}$	0.41595$^{\{6\}}$	0.57868$^{\{10\}}$	0.53449$^{\{9\}}$	0.38549$^{\{2\}}$	0.49498$^{\{8\}}$	0.3382$^{\{1\}}$	0.39126$^{\{3\}}$	0.41564$^{\{5\}}$	0.4237$^{\{7\}}$	0.41125$^{\{4\}}$
		$\hat{b}$	0.2939$^{\{4\}}$	0.38541$^{\{10\}}$	0.38123$^{\{9\}}$	0.28352$^{\{2\}}$	0.35757$^{\{8\}}$	0.27345$^{\{1\}}$	0.29118$^{\{3\}}$	0.33842$^{\{7\}}$	0.33549$^{\{6\}}$	0.3352$^{\{5\}}$
		$\hat{c}$	0.08829$^{\{4\}}$	0.08487$^{\{2\}}$	0.08365$^{\{1\}}$	0.09636$^{\{10\}}$	0.08814$^{\{3\}}$	0.0927$^{\{7\}}$	0.09001$^{\{5\}}$	0.09514$^{\{8\}}$	0.09177$^{\{6\}}$	0.09603$^{\{9\}}$
	MRE	$\hat{a}$	0.24931$^{\{4\}}$	0.30156$^{\{10\}}$	0.28691$^{\{9\}}$	0.23751$^{\{2\}}$	0.27488$^{\{8\}}$	0.22946$^{\{1\}}$	0.24368$^{\{3\}}$	0.252$^{\{5\}}$	0.25804$^{\{7\}}$	0.25438$^{\{6\}}$
		$\hat{b}$	0.46052$^{\{4\}}$	0.53556$^{\{10\}}$	0.53031$^{\{9\}}$	0.44891$^{\{3\}}$	0.51092$^{\{8\}}$	0.42551$^{\{1\}}$	0.44457$^{\{2\}}$	0.4986$^{\{7\}}$	0.49684$^{\{6\}}$	0.49644$^{\{5\}}$
		$\hat{c}$	0.51785$^{\{3\}}$	0.50799$^{\{2\}}$	0.50564$^{\{1\}}$	0.54762$^{\{10\}}$	0.52389$^{\{5\}}$	0.53365$^{\{7\}}$	0.52001$^{\{4\}}$	0.54265$^{\{8\}}$	0.52964$^{\{6\}}$	0.54447$^{\{9\}}$
	D_{abs}		0.03394$^{\{1\}}$	0.03896$^{\{3\}}$	0.03915$^{\{4\}}$	0.03794$^{\{2\}}$	0.03976$^{\{6\}}$	0.04171$^{\{8\}}$	0.03924$^{\{5\}}$	0.05034$^{\{10\}}$	0.04143$^{\{7\}}$	0.05018$^{\{9\}}$
	$D_{\max}$		0.05414$^{\{1\}}$	0.06102$^{\{5\}}$	0.06167$^{\{6\}}$	0.05768$^{\{2\}}$	0.06091$^{\{4\}}$	0.06313$^{\{7\}}$	0.05993$^{\{3\}}$	0.07555$^{\{10\}}$	0.06387$^{\{8\}}$	0.07536$^{\{9\}}$
	ASAE		0.03585$^{\{5\}}$	0.0317$^{\{3\}}$	0.03109$^{\{2\}}$	0.03332$^{\{4\}}$	0.03079$^{\{1\}}$	0.04512$^{\{8\}}$	0.04277$^{\{7\}}$	0.04946$^{\{9\}}$	0.03982$^{\{6\}}$	0.04976$^{\{10\}}$
	$\sum$ Ranks		43$^{\{1\}}$	77$^{\{7\}}$	69$^{\{5\}}$	52$^{\{4\}}$	72$^{\{6\}}$	50$^{\{3\}}$	44$^{\{2\}}$	89$^{\{10\}}$	78$^{\{8\}}$	86$^{\{9\}}$
75	BIAS	$\hat{a}$	0.42199$^{\{4\}}$	0.48182$^{\{9\}}$	0.50652$^{\{10\}}$	0.37717$^{\{1\}}$	0.47381$^{\{8\}}$	0.40067$^{\{2\}}$	0.41393$^{\{3\}}$	0.43153$^{\{6\}}$	0.42623$^{\{5\}}$	0.43738$^{\{7\}}$
		$\hat{b}$	0.40469$^{\{4\}}$	0.4588$^{\{8\}}$	0.4676$^{\{10\}}$	0.39924$^{\{2\}}$	0.46381$^{\{9\}}$	0.38811$^{\{1\}}$	0.40419$^{\{3\}}$	0.43908$^{\{6\}}$	0.42364$^{\{5\}}$	0.44798$^{\{7\}}$
		$\hat{c}$	0.25564$^{\{6\}}$	0.24726$^{\{2\}}$	0.24121$^{\{1\}}$	0.24768$^{\{3\}}$	0.24809$^{\{4\}}$	0.25748$^{\{7\}}$	0.25755$^{\{8\}}$	0.26401$^{\{9\}}$	0.25002$^{\{5\}}$	0.267$^{\{10\}}$
	MSE	$\hat{a}$	0.3016$^{\{4\}}$	0.39816$^{\{9\}}$	0.43551$^{\{10\}}$	0.24732$^{\{1\}}$	0.37399$^{\{8\}}$	0.27604$^{\{2\}}$	0.29194$^{\{3\}}$	0.31238$^{\{6\}}$	0.30788$^{\{5\}}$	0.32961$^{\{7\}}$
		$\hat{b}$	0.23376$^{\{3\}}$	0.2951$^{\{8\}}$	0.30722$^{\{10\}}$	0.23174$^{\{2\}}$	0.30073$^{\{9\}}$	0.23134$^{\{1\}}$	0.24077$^{\{4\}}$	0.27215$^{\{6\}}$	0.25497$^{\{5\}}$	0.27716$^{\{7\}}$
		$\hat{c}$	0.086$^{\{6\}}$	0.08048$^{\{2\}}$	0.07856$^{\{1\}}$	0.08168$^{\{4\}}$	0.08115$^{\{3\}}$	0.08837$^{\{8\}}$	0.08653$^{\{7\}}$	0.09154$^{\{10\}}$	0.08327$^{\{5\}}$	0.09093$^{\{9\}}$
	MRE	$\hat{a}$	0.211$^{\{4\}}$	0.24091$^{\{9\}}$	0.25326$^{\{10\}}$	0.18859$^{\{1\}}$	0.23691$^{\{8\}}$	0.20033$^{\{2\}}$	0.20696$^{\{3\}}$	0.21576$^{\{6\}}$	0.21312$^{\{5\}}$	0.21869$^{\{7\}}$
		$\hat{b}$	0.40469$^{\{4\}}$	0.4588$^{\{8\}}$	0.4676$^{\{10\}}$	0.39924$^{\{2\}}$	0.46381$^{\{9\}}$	0.38811$^{\{1\}}$	0.40419$^{\{3\}}$	0.43908$^{\{6\}}$	0.42364$^{\{5\}}$	0.44798$^{\{7\}}$
		$\hat{c}$	0.51128$^{\{6\}}$	0.49451$^{\{2\}}$	0.48243$^{\{1\}}$	0.49537$^{\{3\}}$	0.49617$^{\{4\}}$	0.51496$^{\{7\}}$	0.5151$^{\{8\}}$	0.52803$^{\{9\}}$	0.50004$^{\{5\}}$	0.534$^{\{10\}}$
	D_{abs}		0.02556$^{\{1\}}$	0.0279$^{\{4\}}$	0.02786$^{\{3\}}$	0.02673$^{\{2\}}$	0.02793$^{\{5\}}$	0.03123$^{\{8\}}$	0.0292$^{\{7\}}$	0.03668$^{\{9\}}$	0.02794$^{\{6\}}$	0.03696$^{\{10\}}$
	$D_{\max}$		0.04042$^{\{1\}}$	0.04396$^{\{4.5\}}$	0.0444$^{\{6\}}$	0.0411$^{\{2\}}$	0.04396$^{\{4.5\}}$	0.04785$^{\{8\}}$	0.04493$^{\{7\}}$	0.05576$^{\{9\}}$	0.04346$^{\{3\}}$	0.05632$^{\{10\}}$
	ASAE		0.02055$^{\{5\}}$	0.0183$^{\{2\}}$	0.01848$^{\{3\}}$	0.01966$^{\{4\}}$	0.01826$^{\{1\}}$	0.02795$^{\{8\}}$	0.02564$^{\{7\}}$	0.03055$^{\{10\}}$	0.02368$^{\{6\}}$	0.03045$^{\{9\}}$
	$\sum$ Ranks		48$^{\{2\}}$	67.5$^{\{6\}}$	75$^{\{8\}}$	27$^{\{1\}}$	72.5$^{\{7\}}$	55$^{\{3\}}$	63$^{\{5\}}$	92$^{\{9\}}$	60$^{\{4\}}$	100$^{\{10\}}$
150	BIAS	$\hat{a}$	0.34123$^{\{2\}}$	0.39411$^{\{8\}}$	0.40191$^{\{9\}}$	0.32335$^{\{1\}}$	0.42027$^{\{10\}}$	0.35459$^{\{5\}}$	0.35124$^{\{4\}}$	0.36751$^{\{6\}}$	0.34612$^{\{3\}}$	0.3775$^{\{7\}}$
		$\hat{b}$	0.36888$^{\{2\}}$	0.39595$^{\{6\}}$	0.41433$^{\{10\}}$	0.36113$^{\{1\}}$	0.41267$^{\{9\}}$	0.36949$^{\{3\}}$	0.37658$^{\{4\}}$	0.39907$^{\{7\}}$	0.38652$^{\{5\}}$	0.40423$^{\{8\}}$
		$\hat{c}$	0.23816$^{\{5\}}$	0.23315$^{\{2\}}$	0.22938$^{\{1\}}$	0.23416$^{\{3\}}$	0.2346$^{\{4\}}$	0.24391$^{\{7\}}$	0.24806$^{\{8\}}$	0.25823$^{\{10\}}$	0.24003$^{\{6\}}$	0.25033$^{\{9\}}$
	MSE	$\hat{a}$	0.19029$^{\{2\}}$	0.26556$^{\{8\}}$	0.27586$^{\{9\}}$	0.1672$^{\{1\}}$	0.30409$^{\{10\}}$	0.22151$^{\{5\}}$	0.20748$^{\{4\}}$	0.22704$^{\{6\}}$	0.19079$^{\{3\}}$	0.24778$^{\{7\}}$
		$\hat{b}$	0.19354$^{\{2\}}$	0.22461$^{\{6\}}$	0.25002$^{\{10\}}$	0.18779$^{\{1\}}$	0.24375$^{\{9\}}$	0.20122$^{\{3\}}$	0.20759$^{\{4\}}$	0.22969$^{\{7\}}$	0.21011$^{\{5\}}$	0.23604$^{\{8\}}$
		$\hat{c}$	0.07748$^{\{6\}}$	0.0748$^{\{4\}}$	0.07206$^{\{1\}}$	0.07433$^{\{2\}}$	0.07467$^{\{3\}}$	0.07966$^{\{7\}}$	0.08087$^{\{8\}}$	0.08664$^{\{10\}}$	0.07673$^{\{5\}}$	0.08228$^{\{9\}}$
	MRE	$\hat{a}$	0.17062$^{\{2\}}$	0.19706$^{\{8\}}$	0.20096$^{\{9\}}$	0.16167$^{\{1\}}$	0.21014$^{\{10\}}$	0.17729$^{\{5\}}$	0.17562$^{\{4\}}$	0.18375$^{\{6\}}$	0.17306$^{\{3\}}$	0.18875$^{\{7\}}$
		$\hat{b}$	0.36888$^{\{2\}}$	0.39595$^{\{6\}}$	0.41433$^{\{10\}}$	0.36113$^{\{1\}}$	0.41267$^{\{9\}}$	0.36949$^{\{3\}}$	0.37658$^{\{4\}}$	0.39907$^{\{7\}}$	0.38652$^{\{5\}}$	0.40423$^{\{8\}}$
		$\hat{c}$	0.47631$^{\{5\}}$	0.46629$^{\{2\}}$	0.45877$^{\{1\}}$	0.46832$^{\{3\}}$	0.46919$^{\{4\}}$	0.48782$^{\{7\}}$	0.49612$^{\{8\}}$	0.51645$^{\{10\}}$	0.48006$^{\{6\}}$	0.50066$^{\{9\}}$
	D_{abs}		0.01816$^{\{1\}}$	0.02004$^{\{5\}}$	0.01979$^{\{4\}}$	0.01948$^{\{2\}}$	0.01966$^{\{3\}}$	0.02413$^{\{8\}}$	0.02247$^{\{7\}}$	0.02821$^{\{10\}}$	0.02105$^{\{6\}}$	0.02804$^{\{9\}}$
	$D_{\max}$		0.02869$^{\{1\}}$	0.03183$^{\{5\}}$	0.03159$^{\{4\}}$	0.0302$^{\{2\}}$	0.03154$^{\{3\}}$	0.03714$^{\{8\}}$	0.03481$^{\{7\}}$	0.0431$^{\{10\}}$	0.03272$^{\{6\}}$	0.04289$^{\{9\}}$
	ASAE		0.01364$^{\{5\}}$	0.0125$^{\{1\}}$	0.01262$^{\{2\}}$	0.01344$^{\{4\}}$	0.01263$^{\{3\}}$	0.01921$^{\{8\}}$	0.01768$^{\{7\}}$	0.02132$^{\{9\}}$	0.01596$^{\{6\}}$	0.02156$^{\{10\}}$
	$\sum$ Ranks		35$^{\{2\}}$	61$^{\{4\}}$	70$^{\{7\}}$	22$^{\{1\}}$	77$^{\{8\}}$	69$^{\{5.5\}}$	69$^{\{5.5\}}$	98$^{\{9\}}$	59$^{\{3\}}$	100$^{\{10\}}$
250	BIAS	$\hat{a}$	0.30906$^{\{4\}}$	0.32753$^{\{5\}}$	0.36715$^{\{10\}}$	0.29328$^{\{1\}}$	0.35535$^{\{9\}}$	0.32872$^{\{7\}}$	0.30662$^{\{3\}}$	0.35018$^{\{8\}}$	0.3025$^{\{2\}}$	0.32764$^{\{6\}}$
		$\hat{b}$	0.33455$^{\{2\}}$	0.35072$^{\{5\}}$	0.38027$^{\{9\}}$	0.3287$^{\{1\}}$	0.37119$^{\{7\}}$	0.34877$^{\{4\}}$	0.35748$^{\{6\}}$	0.38823$^{\{10\}}$	0.33712$^{\{3\}}$	0.37617$^{\{8\}}$
		$\hat{c}$	0.22564$^{\{3\}}$	0.22624$^{\{6\}}$	0.21761$^{\{1\}}$	0.22608$^{\{4\}}$	0.22406$^{\{2\}}$	0.23629$^{\{8\}}$	0.22994$^{\{7\}}$	0.24575$^{\{10\}}$	0.2261$^{\{5\}}$	0.241$^{\{9\}}$
	MSE	$\hat{a}$	0.15437$^{\{3\}}$	0.17224$^{\{5\}}$	0.23785$^{\{10\}}$	0.14157$^{\{1\}}$	0.21415$^{\{8\}}$	0.18019$^{\{6\}}$	0.15526$^{\{4\}}$	0.21944$^{\{9\}}$	0.14692$^{\{2\}}$	0.18205$^{\{7\}}$
		$\hat{b}$	0.16699$^{\{2\}}$	0.18087$^{\{4\}}$	0.21717$^{\{9\}}$	0.16069$^{\{1\}}$	0.20293$^{\{7\}}$	0.18372$^{\{5\}}$	0.18706$^{\{6\}}$	0.2183$^{\{10\}}$	0.16804$^{\{3\}}$	0.20514$^{\{8\}}$
		$\hat{c}$	0.07126$^{\{5\}}$	0.07179$^{\{6\}}$	0.0669$^{\{1\}}$	0.07092$^{\{4\}}$	0.0695$^{\{2\}}$	0.07675$^{\{8\}}$	0.07329$^{\{7\}}$	0.08024$^{\{10\}}$	0.07064$^{\{3\}}$	0.07712$^{\{9\}}$
	MRE	$\hat{a}$	0.15453$^{\{4\}}$	0.16376$^{\{5\}}$	0.18357$^{\{10\}}$	0.14664$^{\{1\}}$	0.17767$^{\{9\}}$	0.16436$^{\{7\}}$	0.15331$^{\{3\}}$	0.17509$^{\{8\}}$	0.15125$^{\{2\}}$	0.16382$^{\{6\}}$
		$\hat{b}$	0.33455$^{\{2\}}$	0.35072$^{\{5\}}$	0.38027$^{\{9\}}$	0.3287$^{\{1\}}$	0.37119$^{\{7\}}$	0.34877$^{\{4\}}$	0.35748$^{\{6\}}$	0.38823$^{\{10\}}$	0.33712$^{\{3\}}$	0.37617$^{\{8\}}$
		$\hat{c}$	0.45128$^{\{3\}}$	0.45248$^{\{6\}}$	0.43523$^{\{1\}}$	0.45216$^{\{4\}}$	0.44813$^{\{2\}}$	0.47257$^{\{8\}}$	0.45989$^{\{7\}}$	0.49149$^{\{10\}}$	0.4522$^{\{5\}}$	0.48199$^{\{9\}}$
	D_{abs}		0.01467$^{\{1\}}$	0.01502$^{\{2\}}$	0.01553$^{\{4\}}$	0.0154$^{\{3\}}$	0.01646$^{\{6\}}$	0.01874$^{\{8\}}$	0.01729$^{\{7\}}$	0.02093$^{\{9\}}$	0.01572$^{\{5\}}$	0.02127$^{\{10\}}$
	$D_{\max}$		0.02327$^{\{1\}}$	0.02391$^{\{2\}}$	0.02519$^{\{5\}}$	0.02402$^{\{3\}}$	0.02632$^{\{6\}}$	0.02913$^{\{8\}}$	0.02696$^{\{7\}}$	0.0326$^{\{9\}}$	0.02461$^{\{4\}}$	0.03285$^{\{10\}}$
	ASAE		0.01005$^{\{5\}}$	0.00938$^{\{1\}}$	0.00951$^{\{2\}}$	0.00987$^{\{4\}}$	0.00959$^{\{3\}}$	0.01453$^{\{8\}}$	0.01322$^{\{7\}}$	0.01619$^{\{10\}}$	0.01227$^{\{6\}}$	0.01584$^{\{9\}}$
	$\sum$ Ranks		35$^{\{2\}}$	52$^{\{4\}}$	71$^{\{7\}}$	28$^{\{1\}}$	68$^{\{5\}}$	81$^{\{8\}}$	70$^{\{6\}}$	113$^{\{10\}}$	43$^{\{3\}}$	99$^{\{9\}}$
400	BIAS	$\hat{a}$	0.27207$^{\{2\}}$	0.30177$^{\{7\}}$	0.31449$^{\{9\}}$	0.25382$^{\{1\}}$	0.31824$^{\{10\}}$	0.27917$^{\{4\}}$	0.28374$^{\{5\}}$	0.30337$^{\{8\}}$	0.27209$^{\{3\}}$	0.29956$^{\{6\}}$
		$\hat{b}$	0.31423$^{\{3\}}$	0.32443$^{\{5\}}$	0.34499$^{\{10\}}$	0.2944$^{\{1\}}$	0.34244$^{\{9\}}$	0.32239$^{\{4\}}$	0.327$^{\{6\}}$	0.33725$^{\{7\}}$	0.31216$^{\{2\}}$	0.34107$^{\{8\}}$
		$\hat{c}$	0.21768$^{\{6\}}$	0.2163$^{\{4\}}$	0.21227$^{\{1\}}$	0.21397$^{\{2\}}$	0.21648$^{\{5\}}$	0.2251$^{\{9\}}$	0.21902$^{\{7\}}$	0.22323$^{\{8\}}$	0.21405$^{\{3\}}$	0.2262$^{\{10\}}$
	MSE	$\hat{a}$	0.1219$^{\{3\}}$	0.14552$^{\{6\}}$	0.16263$^{\{9\}}$	0.10203$^{\{1\}}$	0.17025$^{\{10\}}$	0.1277$^{\{4\}}$	0.13151$^{\{5\}}$	0.15533$^{\{8\}}$	0.12001$^{\{2\}}$	0.14796$^{\{7\}}$
		$\hat{b}$	0.15355$^{\{3\}}$	0.15729$^{\{4\}}$	0.17819$^{\{10\}}$	0.13659$^{\{1\}}$	0.1762$^{\{9\}}$	0.1575$^{\{5\}}$	0.1593$^{\{6\}}$	0.17406$^{\{8\}}$	0.14624$^{\{2\}}$	0.17296$^{\{7\}}$
		$\hat{c}$	0.06679$^{\{7\}}$	0.06674$^{\{6\}}$	0.06485$^{\{1\}}$	0.06538$^{\{2\}}$	0.06672$^{\{5\}}$	0.07027$^{\{9\}}$	0.0665$^{\{4\}}$	0.06975$^{\{8\}}$	0.06557$^{\{3\}}$	0.07069$^{\{10\}}$
	MRE	$\hat{a}$	0.13604$^{\{2.5\}}$	0.15089$^{\{7\}}$	0.15724$^{\{9\}}$	0.12691$^{\{1\}}$	0.15912$^{\{10\}}$	0.13958$^{\{4\}}$	0.14187$^{\{5\}}$	0.15168$^{\{8\}}$	0.13604$^{\{2.5\}}$	0.14978$^{\{6\}}$
		$\hat{b}$	0.31423$^{\{3\}}$	0.32443$^{\{5\}}$	0.34499$^{\{10\}}$	0.2944$^{\{1\}}$	0.34244$^{\{9\}}$	0.32239$^{\{4\}}$	0.327$^{\{6\}}$	0.33725$^{\{7\}}$	0.31216$^{\{2\}}$	0.34107$^{\{8\}}$
		$\hat{c}$	0.43536$^{\{6\}}$	0.43259$^{\{4\}}$	0.42453$^{\{1\}}$	0.42795$^{\{2\}}$	0.43295$^{\{5\}}$	0.4502$^{\{9\}}$	0.43804$^{\{7\}}$	0.44645$^{\{8\}}$	0.42811$^{\{3\}}$	0.4524$^{\{10\}}$
	D_{abs}		0.01176$^{\{2\}}$	0.01174$^{\{1\}}$	0.01243$^{\{4\}}$	0.01182$^{\{3\}}$	0.01294$^{\{6\}}$	0.01426$^{\{8\}}$	0.01383$^{\{7\}}$	0.0168$^{\{9\}}$	0.01284$^{\{5\}}$	0.01691$^{\{10\}}$
	$D_{\max}$		0.01875$^{\{2\}}$	0.01891$^{\{3\}}$	0.0201$^{\{4\}}$	0.01854$^{\{1\}}$	0.02082$^{\{6\}}$	0.02237$^{\{8\}}$	0.02172$^{\{7\}}$	0.02612$^{\{9\}}$	0.02026$^{\{5\}}$	0.02634$^{\{10\}}$
	ASAE		0.00795$^{\{5\}}$	0.00723$^{\{1\}}$	0.00739$^{\{2\}}$	0.00764$^{\{4\}}$	0.00741$^{\{3\}}$	0.01112$^{\{8\}}$	0.01019$^{\{7\}}$	0.01268$^{\{10\}}$	0.00936$^{\{6\}}$	0.01239$^{\{9\}}$
	$\sum$ Ranks		44.5$^{\{3\}}$	53$^{\{4\}}$	70$^{\{5\}}$	20$^{\{1\}}$	87$^{\{8\}}$	76$^{\{7\}}$	72$^{\{6\}}$	98$^{\{9\}}$	38.5$^{\{2\}}$	101$^{\{10\}}$

Table 8: Numerical values for some measures for $a = 2.0,\ b = 1.0,\ c = 0.5$ under RSS.

$s^{\bullet}$	Measure	Est.	ML	AD	CRM	MXPS	LS	SPAD	SPALoD	MSSD	MSSLD	MSLND
30	BIAS	$\hat{a}$	$0.49482^{\{7\}}$	$0.50627^{\{8\}}$	$0.52956^{\{10\}}$	$0.42347^{\{1\}}$	$0.5254^{\{9\}}$	$0.43152^{\{2\}}$	$0.46239^{\{4\}}$	$0.46506^{\{5\}}$	$0.45641^{\{3\}}$	$0.47712^{\{6\}}$
		$\hat{b}$	$0.45437^{\{5\}}$	$0.45829^{\{6\}}$	$0.48435^{\{10\}}$	$0.39979^{\{2\}}$	$0.47098^{\{9\}}$	$0.39483^{\{1\}}$	$0.43662^{\{4\}}$	$0.45968^{\{7\}}$	$0.41499^{\{3\}}$	$0.46498^{\{8\}}$
		$\hat{c}$	$0.24149^{\{3\}}$	$0.23914^{\{1\}}$	$0.2459^{\{5\}}$	$0.23931^{\{2\}}$	$0.24553^{\{4\}}$	$0.25467^{\{7\}}$	$0.259^{\{8\}}$	$0.2647^{\{10\}}$	$0.25282^{\{6\}}$	$0.26081^{\{9\}}$
	MSE	$\hat{a}$	$0.40014^{\{7\}}$	$0.44747^{\{8\}}$	$0.48694^{\{10\}}$	$0.30111^{\{1\}}$	$0.48274^{\{9\}}$	$0.31299^{\{2\}}$	$0.36282^{\{4\}}$	$0.36837^{\{5\}}$	$0.34855^{\{3\}}$	$0.37798^{\{6\}}$
		$\hat{b}$	$0.29212^{\{6\}}$	$0.29718^{\{7\}}$	$0.32367^{\{10\}}$	$0.23246^{\{1\}}$	$0.31362^{\{9\}}$	$0.24034^{\{2\}}$	$0.27591^{\{4\}}$	$0.2908^{\{5\}}$	$0.2482^{\{3\}}$	$0.29959^{\{8\}}$
		$\hat{c}$	$0.07938^{\{3\}}$	$0.07786^{\{2\}}$	$0.07998^{\{5\}}$	$0.07757^{\{1\}}$	$0.07996^{\{4\}}$	$0.08664^{\{7\}}$	$0.08873^{\{9\}}$	$0.09007^{\{10\}}$	$0.08483^{\{6\}}$	$0.08827^{\{8\}}$
	MRE	$\hat{a}$	$0.24741^{\{7\}}$	$0.25314^{\{8\}}$	$0.26478^{\{10\}}$	$0.21174^{\{1\}}$	$0.2627^{\{9\}}$	$0.21576^{\{2\}}$	$0.23119^{\{4\}}$	$0.23253^{\{5\}}$	$0.2282^{\{3\}}$	$0.23856^{\{6\}}$
		$\hat{b}$	$0.45437^{\{5\}}$	$0.45829^{\{6\}}$	$0.48435^{\{10\}}$	$0.39979^{\{2\}}$	$0.47098^{\{9\}}$	$0.39483^{\{1\}}$	$0.43662^{\{4\}}$	$0.45968^{\{7\}}$	$0.41499^{\{3\}}$	$0.46498^{\{8\}}$
		$\hat{c}$	$0.48298^{\{3\}}$	$0.47828^{\{1\}}$	$0.49179^{\{5\}}$	$0.47862^{\{2\}}$	$0.49106^{\{4\}}$	$0.50933^{\{7\}}$	$0.518^{\{8\}}$	$0.5294^{\{10\}}$	$0.50565^{\{6\}}$	$0.52163^{\{9\}}$
	D_{abs}		$0.02467^{\{1\}}$	$0.02649^{\{3\}}$	$0.02737^{\{4\}}$	$0.02594^{\{2\}}$	$0.02921^{\{6\}}$	$0.03345^{\{8\}}$	$0.03138^{\{7\}}$	$0.04193^{\{9\}}$	$0.02828^{\{5\}}$	$0.04315^{\{10\}}$
	$D_{\max}$		$0.03968^{\{2\}}$	$0.04131^{\{3\}}$	$0.04309^{\{4\}}$	$0.03952^{\{1\}}$	$0.0452^{\{6\}}$	$0.05095^{\{8\}}$	$0.04842^{\{7\}}$	$0.06259^{\{9\}}$	$0.04366^{\{5\}}$	$0.06429^{\{10\}}$
	ASAE		$0.03476^{\{5\}}$	$0.03106^{\{3\}}$	$0.03044^{\{1\}}$	$0.03291^{\{4\}}$	$0.03054^{\{2\}}$	$0.04309^{\{8\}}$	$0.04201^{\{7\}}$	$0.04885^{\{10\}}$	$0.03923^{\{6\}}$	$0.0487^{\{9\}}$
	$\sum$ Ranks		$54^{\{3\}}$	$56^{\{5\}}$	$84^{\{8\}}$	$20^{\{1\}}$	$80^{\{7\}}$	$55^{\{4\}}$	$70^{\{6\}}$	$92^{\{9\}}$	$52^{\{2\}}$	$97^{\{10\}}$
75	BIAS	$\hat{a}$	$0.39912^{\{7\}}$	$0.42259^{\{9\}}$	$0.45154^{\{10\}}$	$0.35171^{\{1\}}$	$0.41056^{\{8\}}$	$0.39064^{\{5\}}$	$0.37412^{\{2\}}$	$0.38519^{\{4\}}$	$0.3832^{\{3\}}$	$0.39425^{\{6\}}$
		$\hat{b}$	$0.39748^{\{5\}}$	$0.39968^{\{6\}}$	$0.42908^{\{10\}}$	$0.37133^{\{1\}}$	$0.40688^{\{7\}}$	$0.38423^{\{3\}}$	$0.38675^{\{4\}}$	$0.41481^{\{8\}}$	$0.38351^{\{2\}}$	$0.42009^{\{9\}}$
		$\hat{c}$	$0.22241^{\{2\}}$	$0.23738^{\{7\}}$	$0.231^{\{3\}}$	$0.23368^{\{4\}}$	$0.22087^{\{1\}}$	$0.24659^{\{8\}}$	$0.23495^{\{6\}}$	$0.2553^{\{9\}}$	$0.23402^{\{5\}}$	$0.25903^{\{10\}}$
	MSE	$\hat{a}$	$0.26625^{\{6\}}$	$0.31659^{\{9\}}$	$0.35907^{\{10\}}$	$0.20839^{\{1\}}$	$0.2995^{\{8\}}$	$0.25533^{\{4\}}$	$0.2392^{\{2\}}$	$0.26287^{\{5\}}$	$0.24495^{\{3\}}$	$0.2681^{\{7\}}$
		$\hat{b}$	$0.23228^{\{6\}}$	$0.22998^{\{5\}}$	$0.26455^{\{10\}}$	$0.20293^{\{1\}}$	$0.24466^{\{7\}}$	$0.2213^{\{4\}}$	$0.22107^{\{3\}}$	$0.2449^{\{8\}}$	$0.21427^{\{2\}}$	$0.24814^{\{9\}}$
		$\hat{c}$	$0.06952^{\{2\}}$	$0.07755^{\{7\}}$	$0.07354^{\{4\}}$	$0.07349^{\{3\}}$	$0.06815^{\{1\}}$	$0.08261^{\{8\}}$	$0.07659^{\{6\}}$	$0.08591^{\{10\}}$	$0.07521^{\{5\}}$	$0.08539^{\{9\}}$
	MRE	$\hat{a}$	$0.19956^{\{7\}}$	$0.2113^{\{9\}}$	$0.22577^{\{10\}}$	$0.17585^{\{1\}}$	$0.20528^{\{8\}}$	$0.19532^{\{5\}}$	$0.18706^{\{2\}}$	$0.19259^{\{4\}}$	$0.1916^{\{3\}}$	$0.19713^{\{6\}}$
		$\hat{b}$	$0.39748^{\{5\}}$	$0.39968^{\{6\}}$	$0.42908^{\{10\}}$	$0.37133^{\{1\}}$	$0.40688^{\{7\}}$	$0.38423^{\{3\}}$	$0.38675^{\{4\}}$	$0.41481^{\{8\}}$	$0.38351^{\{2\}}$	$0.42009^{\{9\}}$
		$\hat{c}$	$0.44483^{\{2\}}$	$0.47475^{\{7\}}$	$0.46199^{\{3\}}$	$0.46737^{\{4\}}$	$0.44174^{\{1\}}$	$0.49317^{\{8\}}$	$0.4699^{\{6\}}$	$0.51059^{\{9\}}$	$0.46804^{\{5\}}$	$0.51806^{\{10\}}$
	D_{abs}		$0.01607^{\{1\}}$	$0.01681^{\{3\}}$	$0.01862^{\{5\}}$	$0.01643^{\{2\}}$	$0.01762^{\{4\}}$	$0.0248^{\{8\}}$	$0.02216^{\{7\}}$	$0.03091^{\{9\}}$	$0.02058^{\{6\}}$	$0.03145^{\{10\}}$
	$D_{\max}$		$0.02594^{\{2\}}$	$0.02689^{\{3\}}$	$0.02991^{\{5\}}$	$0.02576^{\{1\}}$	$0.02794^{\{4\}}$	$0.03829^{\{8\}}$	$0.03431^{\{7\}}$	$0.04681^{\{9\}}$	$0.03209^{\{6\}}$	$0.04756^{\{10\}}$
	ASAE		$0.02032^{\{5\}}$	$0.01822^{\{2\}}$	$0.01841^{\{3\}}$	$0.01931^{\{4\}}$	$0.01818^{\{1\}}$	$0.02733^{\{8\}}$	$0.02524^{\{7\}}$	$0.02976^{\{9\}}$	$0.0234^{\{6\}}$	$0.03027^{\{10\}}$
	$\sum$ Ranks		$50^{\{3\}}$	$73^{\{7\}}$	$83^{\{8\}}$	$24^{\{1\}}$	$57^{\{5\}}$	$72^{\{6\}}$	$56^{\{4\}}$	$92^{\{9\}}$	$48^{\{2\}}$	$105^{\{10\}}$
150	BIAS	$\hat{a}$	$0.32925^{\{4\}}$	$0.34679^{\{7\}}$	$0.37667^{\{10\}}$	$0.30382^{\{1\}}$	$0.35962^{\{9\}}$	$0.3268^{\{3\}}$	$0.33642^{\{6\}}$	$0.33278^{\{5\}}$	$0.32672^{\{2\}}$	$0.35143^{\{8\}}$
		$\hat{b}$	$0.3555^{\{4\}}$	$0.35502^{\{3\}}$	$0.37539^{\{9\}}$	$0.33043^{\{1\}}$	$0.36552^{\{7\}}$	$0.35666^{\{5\}}$	$0.35728^{\{6\}}$	$0.37323^{\{8\}}$	$0.35034^{\{2\}}$	$0.39805^{\{10\}}$
		$\hat{c}$	$0.214^{\{4\}}$	$0.21084^{\{2\}}$	$0.21111^{\{3\}}$	$0.21685^{\{5\}}$	$0.20301^{\{1\}}$	$0.23172^{\{8\}}$	$0.22856^{\{7\}}$	$0.24223^{\{10\}}$	$0.22819^{\{6\}}$	$0.24205^{\{9\}}$
	MSE	$\hat{a}$	$0.17658^{\{3\}}$	$0.21163^{\{8\}}$	$0.24678^{\{10\}}$	$0.14745^{\{1\}}$	$0.22908^{\{9\}}$	$0.18165^{\{4\}}$	$0.18708^{\{5\}}$	$0.1904^{\{6\}}$	$0.1681^{\{2\}}$	$0.20879^{\{7\}}$
		$\hat{b}$	$0.18721^{\{3\}}$	$0.18993^{\{6\}}$	$0.2116^{\{9\}}$	$0.1639^{\{1\}}$	$0.20335^{\{8\}}$	$0.1878^{\{4\}}$	$0.18809^{\{5\}}$	$0.20286^{\{7\}}$	$0.17778^{\{2\}}$	$0.22474^{\{10\}}$
		$\hat{c}$	$0.06587^{\{4\}}$	$0.06465^{\{3\}}$	$0.06451^{\{2\}}$	$0.06689^{\{5\}}$	$0.06071^{\{1\}}$	$0.07338^{\{8\}}$	$0.07249^{\{6\}}$	$0.07821^{\{9\}}$	$0.07298^{\{7\}}$	$0.07871^{\{10\}}$
	MRE	$\hat{a}$	$0.16462^{\{4\}}$	$0.17339^{\{7\}}$	$0.18834^{\{10\}}$	$0.15191^{\{1\}}$	$0.17981^{\{9\}}$	$0.1634^{\{3\}}$	$0.16821^{\{6\}}$	$0.16639^{\{5\}}$	$0.16336^{\{2\}}$	$0.17572^{\{8\}}$
		$\hat{b}$	$0.3555^{\{4\}}$	$0.35502^{\{3\}}$	$0.37539^{\{9\}}$	$0.33043^{\{1\}}$	$0.36552^{\{7\}}$	$0.35666^{\{5\}}$	$0.35728^{\{6\}}$	$0.37323^{\{8\}}$	$0.35034^{\{2\}}$	$0.39805^{\{10\}}$
		$\hat{c}$	$0.42801^{\{4\}}$	$0.42167^{\{2\}}$	$0.42222^{\{3\}}$	$0.43369^{\{5\}}$	$0.40601^{\{1\}}$	$0.46345^{\{8\}}$	$0.45712^{\{7\}}$	$0.48445^{\{10\}}$	$0.45638^{\{6\}}$	$0.48409^{\{9\}}$
	D_{abs}		$0.01158^{\{1\}}$	$0.01252^{\{4\}}$	$0.01326^{\{5\}}$	$0.01166^{\{2\}}$	$0.0125^{\{3\}}$	$0.01792^{\{8\}}$	$0.01595^{\{7\}}$	$0.02227^{\{9\}}$	$0.01539^{\{6\}}$	$0.02234^{\{10\}}$
	$D_{\max}$		$0.01895^{\{2\}}$	$0.02019^{\{3\}}$	$0.02164^{\{5\}}$	$0.01851^{\{1\}}$	$0.02035^{\{4\}}$	$0.02779^{\{8\}}$	$0.02497^{\{7\}}$	$0.03409^{\{9\}}$	$0.02419^{\{6\}}$	$0.0344^{\{10\}}$
	ASAE		$0.01322^{\{5\}}$	$0.01223^{\{1.5\}}$	$0.01247^{\{3\}}$	$0.0129^{\{4\}}$	$0.01223^{\{1.5\}}$	$0.01872^{\{8\}}$	$0.01706^{\{7\}}$	$0.02102^{\{9\}}$	$0.01593^{\{6\}}$	$0.02135^{\{10\}}$
	$\sum$ Ranks		$42^{\{2\}}$	$49.5^{\{4\}}$	$78^{\{8\}}$	$28^{\{1\}}$	$60.5^{\{5\}}$	$72^{\{6\}}$	$75^{\{7\}}$	$95^{\{9\}}$	$49^{\{3\}}$	$111^{\{10\}}$
250	BIAS	$\hat{a}$	$0.29295^{\{2\}}$	$0.29661^{\{4\}}$	$0.3162^{\{10\}}$	$0.25523^{\{1\}}$	$0.31446^{\{9\}}$	$0.30305^{\{6\}}$	$0.30026^{\{5\}}$	$0.30714^{\{7\}}$	$0.2963^{\{3\}}$	$0.31115^{\{8\}}$
		$\hat{b}$	$0.33211^{\{6\}}$	$0.31624^{\{2\}}$	$0.33058^{\{5\}}$	$0.30031^{\{1\}}$	$0.33877^{\{8\}}$	$0.32324^{\{3\}}$	$0.33372^{\{7\}}$	$0.34609^{\{10\}}$	$0.32682^{\{4\}}$	$0.34553^{\{9\}}$
		$\hat{c}$	$0.2042^{\{5\}}$	$0.2014^{\{2\}}$	$0.20355^{\{4\}}$	$0.2013^{\{1\}}$	$0.20161^{\{3\}}$	$0.22442^{\{8\}}$	$0.22332^{\{7\}}$	$0.23446^{\{10\}}$	$0.21228^{\{6\}}$	$0.23285^{\{9\}}$
	MSE	$\hat{a}$	$0.14001^{\{3\}}$	$0.15749^{\{6\}}$	$0.17487^{\{10\}}$	$0.10384^{\{1\}}$	$0.17354^{\{9\}}$	$0.15712^{\{5\}}$	$0.14191^{\{4\}}$	$0.16958^{\{8\}}$	$0.13864^{\{2\}}$	$0.16536^{\{7\}}$
		$\hat{b}$	$0.16675^{\{6\}}$	$0.15826^{\{2\}}$	$0.17338^{\{7\}}$	$0.13787^{\{1\}}$	$0.17585^{\{8\}}$	$0.16042^{\{3\}}$	$0.16546^{\{5\}}$	$0.17877^{\{9\}}$	$0.16114^{\{4\}}$	$0.18002^{\{10\}}$
		$\hat{c}$	$0.06123^{\{4\}}$	$0.06021^{\{1\}}$	$0.06035^{\{2\}}$	$0.06125^{\{5\}}$	$0.06061^{\{3\}}$	$0.07152^{\{8\}}$	$0.07038^{\{7\}}$	$0.07443^{\{9\}}$	$0.06442^{\{6\}}$	$0.07453^{\{10\}}$
	MRE	$\hat{a}$	$0.14648^{\{2\}}$	$0.14831^{\{4\}}$	$0.1581^{\{10\}}$	$0.12762^{\{1\}}$	$0.15723^{\{9\}}$	$0.15153^{\{6\}}$	$0.15013^{\{5\}}$	$0.15357^{\{7\}}$	$0.14815^{\{3\}}$	$0.15557^{\{8\}}$
		$\hat{b}$	$0.33211^{\{6\}}$	$0.31624^{\{2\}}$	$0.33058^{\{5\}}$	$0.30031^{\{1\}}$	$0.33877^{\{8\}}$	$0.32324^{\{3\}}$	$0.33372^{\{7\}}$	$0.34609^{\{10\}}$	$0.32682^{\{4\}}$	$0.34553^{\{9\}}$
		$\hat{c}$	$0.4084^{\{5\}}$	$0.40279^{\{2\}}$	$0.4071^{\{4\}}$	$0.40259^{\{1\}}$	$0.40323^{\{3\}}$	$0.44884^{\{8\}}$	$0.44664^{\{7\}}$	$0.46892^{\{10\}}$	$0.42456^{\{6\}}$	$0.4657^{\{9\}}$
	D_{abs}		$0.00908^{\{2\}}$	$0.00929^{\{3\}}$	$0.00993^{\{5\}}$	$0.009^{\{1\}}$	$0.00955^{\{4\}}$	$0.01445^{\{8\}}$	$0.01254^{\{7\}}$	$0.01743^{\{9\}}$	$0.01184^{\{6\}}$	$0.0177^{\{10\}}$
	$D_{\max}$		$0.01494^{\{2\}}$	$0.01525^{\{3\}}$	$0.01633^{\{5\}}$	$0.01436^{\{1\}}$	$0.01582^{\{4\}}$	$0.02261^{\{8\}}$	$0.01985^{\{7\}}$	$0.027^{\{9\}}$	$0.01883^{\{6\}}$	$0.0274^{\{10\}}$
	ASAE		$0.00991^{\{5\}}$	$0.00928^{\{1\}}$	$0.00947^{\{3\}}$	$0.00963^{\{4\}}$	$0.00938^{\{2\}}$	$0.01387^{\{8\}}$	$0.0129^{\{7\}}$	$0.01606^{\{10\}}$	$0.01184^{\{6\}}$	$0.0157^{\{9\}}$
	$\sum$ Ranks		$48^{\{3\}}$	$32^{\{2\}}$	$70^{\{5.5\}}$	$19^{\{1\}}$	$70^{\{5.5\}}$	$74^{\{7\}}$	$75^{\{8\}}$	$108^{\{9.5\}}$	$56^{\{4\}}$	$108^{\{9.5\}}$
400	BIAS	$\hat{a}$	$0.24576^{\{2\}}$	$0.25244^{\{4\}}$	$0.29074^{\{10\}}$	$0.22859^{\{1\}}$	$0.27637^{\{7\}}$	$0.26718^{\{6\}}$	$0.26002^{\{5\}}$	$0.28409^{\{8\}}$	$0.24763^{\{3\}}$	$0.28628^{\{9\}}$
		$\hat{b}$	$0.29685^{\{4\}}$	$0.292^{\{3\}}$	$0.31961^{\{8\}}$	$0.28002^{\{1\}}$	$0.31725^{\{7\}}$	$0.30901^{\{6\}}$	$0.30643^{\{5\}}$	$0.32179^{\{9\}}$	$0.28445^{\{2\}}$	$0.32943^{\{10\}}$
		$\hat{c}$	$0.19274^{\{3\}}$	$0.19029^{\{2\}}$	$0.1951^{\{5\}}$	$0.18839^{\{1\}}$	$0.19342^{\{4\}}$	$0.20707^{\{8\}}$	$0.20224^{\{7\}}$	$0.2203^{\{10\}}$	$0.19523^{\{6\}}$	$0.22002^{\{9\}}$
	MSE	$\hat{a}$	$0.09839^{\{2\}}$	$0.10479^{\{4\}}$	$0.14189^{\{10\}}$	$0.08474^{\{1\}}$	$0.12876^{\{7\}}$	$0.11767^{\{6\}}$	$0.1073^{\{5\}}$	$0.13225^{\{8\}}$	$0.09871^{\{3\}}$	$0.1349^{\{9\}}$
		$\hat{b}$	$0.13844^{\{4\}}$	$0.13345^{\{3\}}$	$0.16465^{\{10\}}$	$0.1263^{\{1\}}$	$0.15751^{\{8\}}$	$0.14641^{\{6\}}$	$0.14555^{\{5\}}$	$0.15635^{\{7\}}$	$0.12686^{\{2\}}$	$0.16129^{\{9\}}$
		$\hat{c}$	$0.05614^{\{3\}}$	$0.05551^{\{2\}}$	$0.05826^{\{6\}}$	$0.05461^{\{1\}}$	$0.05736^{\{5\}}$	$0.06266^{\{8\}}$	$0.06012^{\{7\}}$	$0.06806^{\{9\}}$	$0.05621^{\{4\}}$	$0.06839^{\{10\}}$
	MRE	$\hat{a}$	$0.12288^{\{2\}}$	$0.12622^{\{4\}}$	$0.14537^{\{10\}}$	$0.1143^{\{1\}}$	$0.13819^{\{7\}}$	$0.13359^{\{6\}}$	$0.13001^{\{5\}}$	$0.14205^{\{8\}}$	$0.12381^{\{3\}}$	$0.14314^{\{9\}}$
		$\hat{b}$	$0.29685^{\{4\}}$	$0.292^{\{3\}}$	$0.31961^{\{8\}}$	$0.28002^{\{1\}}$	$0.31725^{\{7\}}$	$0.30901^{\{6\}}$	$0.30643^{\{5\}}$	$0.32179^{\{9\}}$	$0.28445^{\{2\}}$	$0.32943^{\{10\}}$
		$\hat{c}$	$0.38548^{\{3\}}$	$0.38059^{\{2\}}$	$0.39021^{\{5\}}$	$0.37678^{\{1\}}$	$0.38683^{\{4\}}$	$0.41413^{\{8\}}$	$0.40448^{\{7\}}$	$0.44061^{\{10\}}$	$0.39046^{\{6\}}$	$0.44005^{\{9\}}$
	D_{abs}		$0.00718^{\{2\}}$	$0.00738^{\{3\}}$	$0.00767^{\{4\}}$	$0.00692^{\{1\}}$	$0.00779^{\{5\}}$	$0.01134^{\{8\}}$	$0.01039^{\{7\}}$	$0.01405^{\{10\}}$	$0.00893^{\{6\}}$	$0.01404^{\{9\}}$
	$D_{\max}$		$0.01187^{\{2\}}$	$0.01222^{\{3\}}$	$0.0129^{\{5\}}$	$0.01126^{\{1\}}$	$0.01289^{\{4\}}$	$0.01798^{\{8\}}$	$0.01648^{\{7\}}$	$0.022^{\{10\}}$	$0.01427^{\{6\}}$	$0.02194^{\{9\}}$
	ASAE		$0.00762^{\{5\}}$	$0.00718^{\{1\}}$	$0.00734^{\{3\}}$	$0.00753^{\{4\}}$	$0.0073^{\{2\}}$	$0.01107^{\{8\}}$	$0.01007^{\{7\}}$	$0.01254^{\{10\}}$	$0.00927^{\{6\}}$	$0.01223^{\{9\}}$
	$\sum$ Ranks		$36^{\{3\}}$	$34^{\{2\}}$	$84^{\{7.5\}}$	$15^{\{1\}}$	$67^{\{5\}}$	$84^{\{7.5\}}$	$72^{\{6\}}$	$108^{\{9\}}$	$49^{\{4\}}$	$111^{\{10\}}$

Table 9: Numerical values for some measures for $a = 1.0,\ b = 0.25,\ c = 0.9$ under SRS.

$s^{\bullet}$	Measure	Est.	ML	AD	CRM	MXPS	LS	SPAD	SPALoD	MSSD	MSSLD	MSLND
30	BIAS	$\hat{a}$	0.25729$^{\{4\}}$	0.26676$^{\{5\}}$	0.27323$^{\{8\}}$	0.23892$^{\{1\}}$	0.27058$^{\{6\}}$	0.27723$^{\{10\}}$	0.25524$^{\{3\}}$	0.27416$^{\{9\}}$	0.24956$^{\{2\}}$	0.27106$^{\{7\}}$
		$\hat{b}$	0.1213$^{\{4\}}$	0.12661$^{\{9\}}$	0.12782$^{\{10\}}$	0.11972$^{\{1\}}$	0.12614$^{\{8\}}$	0.12436$^{\{7\}}$	0.12018$^{\{2\}}$	0.12311$^{\{6\}}$	0.12229$^{\{5\}}$	0.12113$^{\{3\}}$
		$\hat{c}$	0.14396$^{\{1\}}$	0.15152$^{\{4\}}$	0.1579$^{\{6\}}$	0.14601$^{\{3\}}$	0.15513$^{\{5\}}$	0.17604$^{\{10\}}$	0.1586$^{\{7\}}$	0.16599$^{\{8\}}$	0.14485$^{\{2\}}$	0.17095$^{\{9\}}$
	MSE	$\hat{a}$	0.11112$^{\{4\}}$	0.11629$^{\{6\}}$	0.11989$^{\{9\}}$	0.09407$^{\{1\}}$	0.11721$^{\{7\}}$	0.12361$^{\{10\}}$	0.10534$^{\{3\}}$	0.11857$^{\{8\}}$	0.09934$^{\{2\}}$	0.11424$^{\{5\}}$
		$\hat{b}$	0.01929$^{\{2\}}$	0.02076$^{\{8\}}$	0.02164$^{\{10\}}$	0.01935$^{\{3\}}$	0.02084$^{\{9\}}$	0.02055$^{\{7\}}$	0.01904$^{\{1\}}$	0.01992$^{\{6\}}$	0.0198$^{\{5\}}$	0.01971$^{\{4\}}$
		$\hat{c}$	0.03207$^{\{2\}}$	0.03656$^{\{4\}}$	0.04126$^{\{6\}}$	0.03332$^{\{3\}}$	0.04113$^{\{5\}}$	0.05118$^{\{10\}}$	0.04133$^{\{7\}}$	0.04375$^{\{8\}}$	0.03158$^{\{1\}}$	0.04439$^{\{9\}}$
	MRE	$\hat{a}$	0.25729$^{\{4\}}$	0.26676$^{\{5\}}$	0.27323$^{\{8\}}$	0.23892$^{\{1\}}$	0.27058$^{\{6\}}$	0.27723$^{\{10\}}$	0.25524$^{\{3\}}$	0.27416$^{\{9\}}$	0.24956$^{\{2\}}$	0.27106$^{\{7\}}$
		$\hat{b}$	0.48521$^{\{4\}}$	0.50644$^{\{9\}}$	0.51129$^{\{10\}}$	0.47888$^{\{1\}}$	0.50457$^{\{8\}}$	0.49746$^{\{7\}}$	0.48073$^{\{2\}}$	0.49243$^{\{6\}}$	0.48917$^{\{5\}}$	0.48453$^{\{3\}}$
		$\hat{c}$	0.15996$^{\{1\}}$	0.16836$^{\{4\}}$	0.17544$^{\{6\}}$	0.16223$^{\{3\}}$	0.17237$^{\{5\}}$	0.1956$^{\{10\}}$	0.17622$^{\{7\}}$	0.18444$^{\{8\}}$	0.16095$^{\{2\}}$	0.18994$^{\{9\}}$
	D_{abs}		0.04027$^{\{1\}}$	0.0432$^{\{3\}}$	0.04179$^{\{2\}}$	0.0434$^{\{4\}}$	0.04503$^{\{7\}}$	0.04901$^{\{9\}}$	0.04446$^{\{6\}}$	0.04834$^{\{8\}}$	0.04442$^{\{5\}}$	0.05086$^{\{10\}}$
	$D_{\max}$		0.06256$^{\{1\}}$	0.06644$^{\{5\}}$	0.06561$^{\{3\}}$	0.06412$^{\{2\}}$	0.06847$^{\{7\}}$	0.07339$^{\{9\}}$	0.06628$^{\{4\}}$	0.07127$^{\{8\}}$	0.06652$^{\{6\}}$	0.0745$^{\{10\}}$
	ASAE		0.03558$^{\{5\}}$	0.03224$^{\{3\}}$	0.03188$^{\{2\}}$	0.03481$^{\{4\}}$	0.03154$^{\{1\}}$	0.04739$^{\{10\}}$	0.04364$^{\{7\}}$	0.04522$^{\{8\}}$	0.03956$^{\{6\}}$	0.0462$^{\{9\}}$
	$\sum$ Ranks		33$^{\{2\}}$	65$^{\{5\}}$	80$^{\{7\}}$	27$^{\{1\}}$	74$^{\{6\}}$	109$^{\{10\}}$	52$^{\{4\}}$	92$^{\{9\}}$	43$^{\{3\}}$	85$^{\{8\}}$
75	BIAS	$\hat{a}$	0.18822$^{\{4\}}$	0.18761$^{\{3\}}$	0.19928$^{\{7\}}$	0.16563$^{\{1\}}$	0.19423$^{\{6\}}$	0.20331$^{\{8\}}$	0.19364$^{\{5\}}$	0.21085$^{\{9\}}$	0.18399$^{\{2\}}$	0.21267$^{\{10\}}$
		$\hat{b}$	0.11067$^{\{3\}}$	0.11252$^{\{6\}}$	0.11159$^{\{4\}}$	0.1012$^{\{1\}}$	0.11586$^{\{9\}}$	0.11238$^{\{5\}}$	0.11329$^{\{8\}}$	0.11267$^{\{7\}}$	0.1075$^{\{2\}}$	0.11619$^{\{10\}}$
		$\hat{c}$	0.10565$^{\{2\}}$	0.11044$^{\{3\}}$	0.11433$^{\{5\}}$	0.10508$^{\{1\}}$	0.11784$^{\{6\}}$	0.12753$^{\{9\}}$	0.12259$^{\{7\}}$	0.12656$^{\{8\}}$	0.11052$^{\{4\}}$	0.13171$^{\{10\}}$
	MSE	$\hat{a}$	0.05807$^{\{3\}}$	0.05875$^{\{4\}}$	0.06472$^{\{7\}}$	0.04384$^{\{1\}}$	0.06116$^{\{6\}}$	0.07008$^{\{8\}}$	0.0605$^{\{5\}}$	0.07391$^{\{10\}}$	0.05566$^{\{2\}}$	0.0729$^{\{9\}}$
		$\hat{b}$	0.01652$^{\{3\}}$	0.01707$^{\{5\}}$	0.01681$^{\{4\}}$	0.01455$^{\{1\}}$	0.01812$^{\{10\}}$	0.01729$^{\{6\}}$	0.01752$^{\{8\}}$	0.01746$^{\{7\}}$	0.01594$^{\{2\}}$	0.01799$^{\{9\}}$
		$\hat{c}$	0.01816$^{\{2\}}$	0.01998$^{\{4\}}$	0.02136$^{\{5\}}$	0.01733$^{\{1\}}$	0.02261$^{\{6\}}$	0.02689$^{\{9\}}$	0.02427$^{\{7\}}$	0.02534$^{\{8\}}$	0.01868$^{\{3\}}$	0.02716$^{\{10\}}$
	MRE	$\hat{a}$	0.18822$^{\{4\}}$	0.18761$^{\{3\}}$	0.19928$^{\{7\}}$	0.16563$^{\{1\}}$	0.19423$^{\{6\}}$	0.20331$^{\{8\}}$	0.19364$^{\{5\}}$	0.21085$^{\{9\}}$	0.18399$^{\{2\}}$	0.21267$^{\{10\}}$
		$\hat{b}$	0.44267$^{\{3\}}$	0.45007$^{\{6\}}$	0.44638$^{\{4\}}$	0.40478$^{\{1\}}$	0.46345$^{\{9\}}$	0.44954$^{\{5\}}$	0.45317$^{\{8\}}$	0.45067$^{\{7\}}$	0.43001$^{\{2\}}$	0.46475$^{\{10\}}$
		$\hat{c}$	0.11739$^{\{2\}}$	0.12271$^{\{3\}}$	0.12703$^{\{5\}}$	0.11675$^{\{1\}}$	0.13093$^{\{6\}}$	0.1417$^{\{9\}}$	0.13621$^{\{7\}}$	0.14062$^{\{8\}}$	0.1228$^{\{4\}}$	0.14635$^{\{10\}}$
	D_{abs}		0.0279$^{\{2\}}$	0.02796$^{\{3\}}$	0.02989$^{\{5\}}$	0.02782$^{\{1\}}$	0.02939$^{\{4\}}$	0.03337$^{\{9\}}$	0.03272$^{\{7\}}$	0.03329$^{\{8\}}$	0.03047$^{\{6\}}$	0.03531$^{\{10\}}$
	$D_{\max}$		0.04451$^{\{3\}}$	0.04442$^{\{2\}}$	0.04741$^{\{6\}}$	0.04262$^{\{1\}}$	0.04661$^{\{4\}}$	0.05163$^{\{9\}}$	0.05042$^{\{7\}}$	0.05133$^{\{8\}}$	0.04722$^{\{5\}}$	0.05382$^{\{10\}}$
	ASAE		0.02097$^{\{5\}}$	0.01935$^{\{3\}}$	0.01932$^{\{2\}}$	0.02092$^{\{4\}}$	0.01897$^{\{1\}}$	0.03019$^{\{10\}}$	0.02668$^{\{7\}}$	0.02996$^{\{9\}}$	0.02459$^{\{6\}}$	0.0298$^{\{8\}}$
	$\sum$ Ranks		36$^{\{2\}}$	45$^{\{4\}}$	61$^{\{5\}}$	15$^{\{1\}}$	73$^{\{6\}}$	95$^{\{8\}}$	81$^{\{7\}}$	98$^{\{9\}}$	40$^{\{3\}}$	116$^{\{10\}}$
150	BIAS	$\hat{a}$	0.14225$^{\{2\}}$	0.14403$^{\{3\}}$	0.15692$^{\{7\}}$	0.13112$^{\{1\}}$	0.15001$^{\{5\}}$	0.15775$^{\{8\}}$	0.15033$^{\{6\}}$	0.16659$^{\{10\}}$	0.14779$^{\{4\}}$	0.16378$^{\{9\}}$
		$\hat{b}$	0.09452$^{\{1\}}$	0.09666$^{\{3\}}$	0.10078$^{\{5\}}$	0.09535$^{\{2\}}$	0.10337$^{\{8\}}$	0.10324$^{\{7\}}$	0.10184$^{\{6\}}$	0.1057$^{\{10\}}$	0.09803$^{\{4\}}$	0.10479$^{\{9\}}$
		$\hat{c}$	0.08538$^{\{4\}}$	0.08409$^{\{3\}}$	0.09358$^{\{7\}}$	0.0783$^{\{1\}}$	0.08935$^{\{5\}}$	0.10499$^{\{10\}}$	0.09189$^{\{6\}}$	0.10185$^{\{9\}}$	0.08384$^{\{2\}}$	0.09784$^{\{8\}}$
	MSE	$\hat{a}$	0.03345$^{\{3\}}$	0.03328$^{\{2\}}$	0.03909$^{\{7\}}$	0.02583$^{\{1\}}$	0.03614$^{\{6\}}$	0.0397$^{\{8\}}$	0.03593$^{\{5\}}$	0.04544$^{\{10\}}$	0.03504$^{\{4\}}$	0.0445$^{\{9\}}$
		$\hat{b}$	0.01247$^{\{1\}}$	0.0133$^{\{3\}}$	0.01428$^{\{5\}}$	0.0131$^{\{2\}}$	0.01513$^{\{8\}}$	0.01485$^{\{7\}}$	0.01454$^{\{6\}}$	0.0156$^{\{10\}}$	0.01359$^{\{4\}}$	0.01538$^{\{9\}}$
		$\hat{c}$	0.01191$^{\{4\}}$	0.01183$^{\{3\}}$	0.01485$^{\{7\}}$	0.0093$^{\{1\}}$	0.01389$^{\{6\}}$	0.0176$^{\{10\}}$	0.01333$^{\{5\}}$	0.0165$^{\{9\}}$	0.01103$^{\{2\}}$	0.01568$^{\{8\}}$
	MRE	$\hat{a}$	0.14225$^{\{2\}}$	0.14403$^{\{3\}}$	0.15692$^{\{7\}}$	0.13112$^{\{1\}}$	0.15001$^{\{5\}}$	0.15775$^{\{8\}}$	0.15033$^{\{6\}}$	0.16659$^{\{10\}}$	0.14779$^{\{4\}}$	0.16378$^{\{9\}}$
		$\hat{b}$	0.37807$^{\{1\}}$	0.38663$^{\{3\}}$	0.40313$^{\{5\}}$	0.38141$^{\{2\}}$	0.4135$^{\{8\}}$	0.41296$^{\{7\}}$	0.40737$^{\{6\}}$	0.4228$^{\{10\}}$	0.3921$^{\{4\}}$	0.41916$^{\{9\}}$
		$\hat{c}$	0.09486$^{\{4\}}$	0.09343$^{\{3\}}$	0.10397$^{\{7\}}$	0.087$^{\{1\}}$	0.09928$^{\{5\}}$	0.11665$^{\{10\}}$	0.1021$^{\{6\}}$	0.11317$^{\{9\}}$	0.09315$^{\{2\}}$	0.10871$^{\{8\}}$
	D_{abs}		0.02004$^{\{3\}}$	0.01986$^{\{2\}}$	0.02175$^{\{6\}}$	0.01968$^{\{1\}}$	0.02048$^{\{4\}}$	0.02482$^{\{8\}}$	0.02331$^{\{7\}}$	0.02588$^{\{10\}}$	0.02132$^{\{5\}}$	0.02516$^{\{9\}}$
	$D_{\max}$		0.03236$^{\{3\}}$	0.03214$^{\{2\}}$	0.03534$^{\{6\}}$	0.03111$^{\{1\}}$	0.03328$^{\{4\}}$	0.03912$^{\{8\}}$	0.03677$^{\{7\}}$	0.04053$^{\{10\}}$	0.03391$^{\{5\}}$	0.03956$^{\{9\}}$
	ASAE		0.01479$^{\{5\}}$	0.01327$^{\{2\}}$	0.0134$^{\{3\}}$	0.01436$^{\{4\}}$	0.01309$^{\{1\}}$	0.02049$^{\{8\}}$	0.01861$^{\{7\}}$	0.02128$^{\{9\}}$	0.01712$^{\{6\}}$	0.02162$^{\{10\}}$
	$\sum$ Ranks		33$^{\{3\}}$	32$^{\{2\}}$	72$^{\{6\}}$	18$^{\{1\}}$	65$^{\{5\}}$	99$^{\{8\}}$	73$^{\{7\}}$	116$^{\{10\}}$	46$^{\{4\}}$	106$^{\{9\}}$
250	BIAS	$\hat{a}$	0.11318$^{\{3\}}$	0.10923$^{\{1\}}$	0.12619$^{\{7\}}$	0.11005$^{\{2\}}$	0.12217$^{\{6\}}$	0.12947$^{\{8\}}$	0.11768$^{\{4\}}$	0.14418$^{\{10\}}$	0.11909$^{\{5\}}$	0.13909$^{\{9\}}$
		$\hat{b}$	0.0822$^{\{1.5\}}$	0.0822$^{\{1.5\}}$	0.08917$^{\{6\}}$	0.08595$^{\{3\}}$	0.08704$^{\{4\}}$	0.09255$^{\{8\}}$	0.09016$^{\{7\}}$	0.09708$^{\{10\}}$	0.08896$^{\{5\}}$	0.09391$^{\{9\}}$
		$\hat{c}$	0.07008$^{\{2\}}$	0.07012$^{\{3\}}$	0.07933$^{\{5\}}$	0.06709$^{\{1\}}$	0.07968$^{\{6\}}$	0.08523$^{\{9\}}$	0.08047$^{\{7\}}$	0.08548$^{\{10\}}$	0.07327$^{\{4\}}$	0.08332$^{\{8\}}$
	MSE	$\hat{a}$	0.02011$^{\{3\}}$	0.01852$^{\{2\}}$	0.02507$^{\{7\}}$	0.0184$^{\{1\}}$	0.02312$^{\{6\}}$	0.0275$^{\{8\}}$	0.02147$^{\{4\}}$	0.03266$^{\{10\}}$	0.02226$^{\{5\}}$	0.03226$^{\{9\}}$
		$\hat{b}$	0.01004$^{\{1\}}$	0.01043$^{\{2\}}$	0.01163$^{\{5\}}$	0.01127$^{\{3\}}$	0.01137$^{\{4\}}$	0.01252$^{\{8\}}$	0.01219$^{\{7\}}$	0.01349$^{\{10\}}$	0.01181$^{\{6\}}$	0.01275$^{\{9\}}$
		$\hat{c}$	0.00754$^{\{2\}}$	0.00827$^{\{4\}}$	0.01176$^{\{8\}}$	0.00694$^{\{1\}}$	0.0115$^{\{6\}}$	0.01252$^{\{10\}}$	0.01004$^{\{5\}}$	0.01175$^{\{7\}}$	0.00819$^{\{3\}}$	0.01203$^{\{9\}}$
	MRE	$\hat{a}$	0.11318$^{\{3\}}$	0.10923$^{\{1\}}$	0.12619$^{\{7\}}$	0.11005$^{\{2\}}$	0.12217$^{\{6\}}$	0.12947$^{\{8\}}$	0.11768$^{\{4\}}$	0.14418$^{\{10\}}$	0.11909$^{\{5\}}$	0.13909$^{\{9\}}$
		$\hat{b}$	0.32878$^{\{1\}}$	0.32881$^{\{2\}}$	0.35668$^{\{6\}}$	0.34379$^{\{3\}}$	0.34817$^{\{4\}}$	0.3702$^{\{8\}}$	0.36065$^{\{7\}}$	0.38832$^{\{10\}}$	0.35582$^{\{5\}}$	0.37565$^{\{9\}}$
		$\hat{c}$	0.07787$^{\{2\}}$	0.07791$^{\{3\}}$	0.08814$^{\{5\}}$	0.07455$^{\{1\}}$	0.08853$^{\{6\}}$	0.0947$^{\{9\}}$	0.08941$^{\{7\}}$	0.09498$^{\{10\}}$	0.08141$^{\{4\}}$	0.09258$^{\{8\}}$
	D_{abs}		0.01557$^{\{2\}}$	0.01593$^{\{3\}}$	0.01678$^{\{4\}}$	0.01526$^{\{1\}}$	0.01679$^{\{5\}}$	0.01989$^{\{8\}}$	0.01795$^{\{7\}}$	0.02099$^{\{10\}}$	0.01751$^{\{6\}}$	0.02032$^{\{9\}}$
	$D_{\max}$		0.02522$^{\{2\}}$	0.02564$^{\{3\}}$	0.02749$^{\{5\}}$	0.02457$^{\{1\}}$	0.02728$^{\{4\}}$	0.03153$^{\{8\}}$	0.02864$^{\{7\}}$	0.03333$^{\{10\}}$	0.02787$^{\{6\}}$	0.03224$^{\{9\}}$
	ASAE		0.01091$^{\{4\}}$	0.01038$^{\{3\}}$	0.01026$^{\{2\}}$	0.01103$^{\{5\}}$	0.01013$^{\{1\}}$	0.0162$^{\{8\}}$	0.01448$^{\{7\}}$	0.01691$^{\{9\}}$	0.01351$^{\{6\}}$	0.01696$^{\{10\}}$
	$\sum$ Ranks		26.5$^{\{2\}}$	28.5$^{\{3\}}$	67$^{\{6\}}$	24$^{\{1\}}$	58$^{\{4\}}$	100$^{\{8\}}$	73$^{\{7\}}$	116$^{\{10\}}$	60$^{\{5\}}$	107$^{\{9\}}$
400	BIAS	$\hat{a}$	0.08959$^{\{1\}}$	0.09463$^{\{3\}}$	0.10214$^{\{6\}}$	0.09023$^{\{2\}}$	0.10371$^{\{7\}}$	0.10955$^{\{8\}}$	0.10095$^{\{5\}}$	0.11673$^{\{9\}}$	0.09802$^{\{4\}}$	0.12013$^{\{10\}}$
		$\hat{b}$	0.06853$^{\{1\}}$	0.07409$^{\{2\}}$	0.07604$^{\{3.5\}}$	0.07604$^{\{3.5\}}$	0.07672$^{\{5\}}$	0.08258$^{\{8\}}$	0.08079$^{\{7\}}$	0.08872$^{\{9\}}$	0.07791$^{\{6\}}$	0.09071$^{\{10\}}$
		$\hat{c}$	0.05885$^{\{1\}}$	0.06099$^{\{3\}}$	0.06728$^{\{6\}}$	0.05948$^{\{2\}}$	0.06863$^{\{7\}}$	0.07199$^{\{9\}}$	0.06686$^{\{5\}}$	0.06941$^{\{8\}}$	0.06508$^{\{4\}}$	0.07402$^{\{10\}}$
	MSE	$\hat{a}$	0.01262$^{\{2\}}$	0.01395$^{\{3\}}$	0.01652$^{\{7\}}$	0.01224$^{\{1\}}$	0.01624$^{\{6\}}$	0.01861$^{\{8\}}$	0.01612$^{\{5\}}$	0.02107$^{\{9\}}$	0.01479$^{\{4\}}$	0.02298$^{\{10\}}$
		$\hat{b}$	0.00747$^{\{1\}}$	0.00873$^{\{2\}}$	0.00911$^{\{3\}}$	0.00921$^{\{4\}}$	0.00935$^{\{5\}}$	0.01041$^{\{8\}}$	0.01013$^{\{7\}}$	0.01184$^{\{9\}}$	0.00943$^{\{6\}}$	0.01215$^{\{10\}}$
		$\hat{c}$	0.00517$^{\{1\}}$	0.00656$^{\{4\}}$	0.00858$^{\{8\}}$	0.00557$^{\{2\}}$	0.00851$^{\{7\}}$	0.00921$^{\{10\}}$	0.00708$^{\{5\}}$	0.00767$^{\{6\}}$	0.00635$^{\{3\}}$	0.0088$^{\{9\}}$
	MRE	$\hat{a}$	0.08959$^{\{1\}}$	0.09463$^{\{3\}}$	0.10214$^{\{6\}}$	0.09023$^{\{2\}}$	0.10371$^{\{7\}}$	0.10955$^{\{8\}}$	0.10095$^{\{5\}}$	0.11673$^{\{9\}}$	0.09802$^{\{4\}}$	0.12013$^{\{10\}}$
		$\hat{b}$	0.27413$^{\{1\}}$	0.29637$^{\{2\}}$	0.30418$^{\{4\}}$	0.30416$^{\{3\}}$	0.30686$^{\{5\}}$	0.33031$^{\{8\}}$	0.32317$^{\{7\}}$	0.35488$^{\{9\}}$	0.31164$^{\{6\}}$	0.36283$^{\{10\}}$
		$\hat{c}$	0.06539$^{\{1\}}$	0.06777$^{\{3\}}$	0.07475$^{\{6\}}$	0.06609$^{\{2\}}$	0.07626$^{\{7\}}$	0.07999$^{\{9\}}$	0.07429$^{\{5\}}$	0.07712$^{\{8\}}$	0.07231$^{\{4\}}$	0.08225$^{\{10\}}$
	D_{abs}		0.01241$^{\{1\}}$	0.01308$^{\{4\}}$	0.01319$^{\{5\}}$	0.01258$^{\{2\}}$	0.013$^{\{3\}}$	0.01524$^{\{8\}}$	0.01426$^{\{7\}}$	0.01656$^{\{9\}}$	0.01419$^{\{6\}}$	0.01703$^{\{10\}}$
	$D_{\max}$		0.02014$^{\{1\}}$	0.02113$^{\{3\}}$	0.02164$^{\{5\}}$	0.02025$^{\{2\}}$	0.02131$^{\{4\}}$	0.02464$^{\{8\}}$	0.02295$^{\{7\}}$	0.0266$^{\{9\}}$	0.02281$^{\{6\}}$	0.02732$^{\{10\}}$
	ASAE		0.00877$^{\{5\}}$	0.00805$^{\{3\}}$	0.00794$^{\{2\}}$	0.0086$^{\{4\}}$	0.00783$^{\{1\}}$	0.01252$^{\{8\}}$	0.01149$^{\{7\}}$	0.01367$^{\{9\}}$	0.01076$^{\{6\}}$	0.01376$^{\{10\}}$
	$\sum$ Ranks		17$^{\{1\}}$	35$^{\{3\}}$	61.5$^{\{5\}}$	29.5$^{\{2\}}$	64$^{\{6\}}$	100$^{\{8\}}$	72$^{\{7\}}$	103$^{\{9\}}$	59$^{\{4\}}$	119$^{\{10\}}$

Table 10: Numerical values for some measures for $a = 1.0,\ b = 0.25,\ c = 0.9$ under RSS.

$s^{\bullet}$	Measure	Est.	ML	AD	CRM	MXPS	LS	SPAD	SPALoD	MSSD	MSSLD	MSLND
30	BIAS	$\hat{a}$	0.20097$^{\{4\}}$	0.20079$^{\{3\}}$	0.22385$^{\{7\}}$	0.18526$^{\{1\}}$	0.19866$^{\{2\}}$	0.23255$^{\{8\}}$	0.22213$^{\{6\}}$	0.23917$^{\{10\}}$	0.21391$^{\{5\}}$	0.23385$^{\{9\}}$
		$\hat{b}$	0.11729$^{\{2\}}$	0.11962$^{\{5\}}$	0.12209$^{\{10\}}$	0.11558$^{\{1\}}$	0.12139$^{\{8\}}$	0.11983$^{\{7\}}$	0.11783$^{\{3\}}$	0.11963$^{\{6\}}$	0.12179$^{\{9\}}$	0.11841$^{\{4\}}$
		$\hat{c}$	0.10712$^{\{2\}}$	0.11019$^{\{4\}}$	0.11429$^{\{5\}}$	0.10063$^{\{1\}}$	0.10961$^{\{3\}}$	0.14709$^{\{10\}}$	0.13239$^{\{7\}}$	0.1415$^{\{9\}}$	0.12049$^{\{6\}}$	0.13844$^{\{8\}}$
	MSE	$\hat{a}$	0.06715$^{\{4\}}$	0.06661$^{\{3\}}$	0.08008$^{\{6\}}$	0.05563$^{\{1\}}$	0.06498$^{\{2\}}$	0.08954$^{\{8\}}$	0.08045$^{\{7\}}$	0.09217$^{\{10\}}$	0.07643$^{\{5\}}$	0.09039$^{\{9\}}$
		$\hat{b}$	0.01847$^{\{2\}}$	0.0191$^{\{7\}}$	0.01994$^{\{10\}}$	0.01817$^{\{1\}}$	0.01946$^{\{8\}}$	0.01899$^{\{5\}}$	0.01853$^{\{3\}}$	0.01902$^{\{6\}}$	0.01964$^{\{9\}}$	0.01874$^{\{4\}}$
		$\hat{c}$	0.01929$^{\{2\}}$	0.02031$^{\{3\}}$	0.02354$^{\{5\}}$	0.01635$^{\{1\}}$	0.02131$^{\{4\}}$	0.03511$^{\{10\}}$	0.02901$^{\{7\}}$	0.03241$^{\{9\}}$	0.02463$^{\{6\}}$	0.03011$^{\{8\}}$
	MRE	$\hat{a}$	0.20097$^{\{4\}}$	0.20079$^{\{3\}}$	0.22385$^{\{7\}}$	0.18526$^{\{1\}}$	0.19866$^{\{2\}}$	0.23255$^{\{8\}}$	0.22213$^{\{6\}}$	0.23917$^{\{10\}}$	0.21391$^{\{5\}}$	0.23385$^{\{9\}}$
		$\hat{b}$	0.46917$^{\{2\}}$	0.47846$^{\{5\}}$	0.48837$^{\{10\}}$	0.46233$^{\{1\}}$	0.48554$^{\{8\}}$	0.47933$^{\{7\}}$	0.47133$^{\{3\}}$	0.4785$^{\{6\}}$	0.48716$^{\{9\}}$	0.47365$^{\{4\}}$
		$\hat{c}$	0.11903$^{\{2\}}$	0.12243$^{\{4\}}$	0.12699$^{\{5\}}$	0.11181$^{\{1\}}$	0.12179$^{\{3\}}$	0.16343$^{\{10\}}$	0.1471$^{\{7\}}$	0.15723$^{\{9\}}$	0.13387$^{\{6\}}$	0.15382$^{\{8\}}$
	D_{abs}		0.02698$^{\{2\}}$	0.02729$^{\{3\}}$	0.02823$^{\{5\}}$	0.02696$^{\{1\}}$	0.02799$^{\{4\}}$	0.03999$^{\{10\}}$	0.03547$^{\{7\}}$	0.03882$^{\{8\}}$	0.03228$^{\{6\}}$	0.03914$^{\{9\}}$
	$D_{\max}$		0.04327$^{\{3\}}$	0.04306$^{\{2\}}$	0.04545$^{\{5\}}$	0.04165$^{\{1\}}$	0.04418$^{\{4\}}$	0.06072$^{\{10\}}$	0.05389$^{\{7\}}$	0.05765$^{\{8\}}$	0.04983$^{\{6\}}$	0.05836$^{\{9\}}$
	ASAE		0.03402$^{\{5\}}$	0.0312$^{\{2\}}$	0.03156$^{\{3\}}$	0.03336$^{\{4\}}$	0.03045$^{\{1\}}$	0.04666$^{\{10\}}$	0.04216$^{\{7\}}$	0.04476$^{\{8\}}$	0.03877$^{\{6\}}$	0.04593$^{\{9\}}$
	$\sum$ Ranks		34$^{\{2\}}$	44$^{\{3\}}$	78$^{\{6.5\}}$	15$^{\{1\}}$	49$^{\{4\}}$	103$^{\{10\}}$	70$^{\{5\}}$	99$^{\{9\}}$	78$^{\{6.5\}}$	90$^{\{8\}}$
75	BIAS	$\hat{a}$	0.14489$^{\{2\}}$	0.1615$^{\{5\}}$	0.16669$^{\{6\}}$	0.13614$^{\{1\}}$	0.15665$^{\{4\}}$	0.17722$^{\{8\}}$	0.1709$^{\{7\}}$	0.18575$^{\{10\}}$	0.15593$^{\{3\}}$	0.18527$^{\{9\}}$
		$\hat{b}$	0.09998$^{\{2\}}$	0.10834$^{\{8\}}$	0.10582$^{\{7\}}$	0.09963$^{\{1\}}$	0.10382$^{\{5\}}$	0.10434$^{\{6\}}$	0.10313$^{\{3\}}$	0.10967$^{\{9\}}$	0.10323$^{\{4\}}$	0.1127$^{\{10\}}$
		$\hat{c}$	0.07618$^{\{2\}}$	0.08364$^{\{3\}}$	0.08972$^{\{6\}}$	0.07537$^{\{1\}}$	0.08451$^{\{4\}}$	0.11523$^{\{10\}}$	0.10446$^{\{7\}}$	0.10831$^{\{9\}}$	0.08734$^{\{5\}}$	0.10802$^{\{8\}}$
	MSE	$\hat{a}$	0.03343$^{\{2\}}$	0.04107$^{\{5\}}$	0.04415$^{\{6\}}$	0.02916$^{\{1\}}$	0.04043$^{\{4\}}$	0.05192$^{\{8\}}$	0.04809$^{\{7\}}$	0.05506$^{\{9\}}$	0.03886$^{\{3\}}$	0.05519$^{\{10\}}$
		$\hat{b}$	0.01399$^{\{1\}}$	0.01637$^{\{8\}}$	0.01545$^{\{6\}}$	0.01431$^{\{2\}}$	0.01512$^{\{5\}}$	0.01548$^{\{7\}}$	0.015$^{\{3\}}$	0.01658$^{\{9\}}$	0.01511$^{\{4\}}$	0.01739$^{\{10\}}$
		$\hat{c}$	0.00993$^{\{2\}}$	0.0127$^{\{4\}}$	0.01699$^{\{6\}}$	0.00925$^{\{1\}}$	0.0135$^{\{5\}}$	0.02185$^{\{10\}}$	0.01857$^{\{7\}}$	0.01949$^{\{8\}}$	0.01214$^{\{3\}}$	0.01957$^{\{9\}}$
	MRE	$\hat{a}$	0.14489$^{\{2\}}$	0.1615$^{\{5\}}$	0.16669$^{\{6\}}$	0.13614$^{\{1\}}$	0.15665$^{\{4\}}$	0.17722$^{\{8\}}$	0.1709$^{\{7\}}$	0.18575$^{\{10\}}$	0.15593$^{\{3\}}$	0.18527$^{\{9\}}$
		$\hat{b}$	0.39991$^{\{2\}}$	0.43335$^{\{8\}}$	0.4233$^{\{7\}}$	0.39852$^{\{1\}}$	0.41527$^{\{5\}}$	0.41736$^{\{6\}}$	0.41253$^{\{3\}}$	0.43868$^{\{9\}}$	0.41292$^{\{4\}}$	0.45082$^{\{10\}}$
		$\hat{c}$	0.08465$^{\{2\}}$	0.09294$^{\{3\}}$	0.09969$^{\{6\}}$	0.08374$^{\{1\}}$	0.0939$^{\{4\}}$	0.12803$^{\{10\}}$	0.11607$^{\{7\}}$	0.12034$^{\{9\}}$	0.09705$^{\{5\}}$	0.12002$^{\{8\}}$
	D_{abs}		0.01766$^{\{2\}}$	0.01821$^{\{3\}}$	0.01831$^{\{4\}}$	0.01744$^{\{1\}}$	0.01878$^{\{5\}}$	0.02727$^{\{9\}}$	0.0249$^{\{7\}}$	0.02718$^{\{8\}}$	0.02234$^{\{6\}}$	0.02758$^{\{10\}}$
	$D_{\max}$		0.02921$^{\{2\}}$	0.03005$^{\{3\}}$	0.03059$^{\{4\}}$	0.02812$^{\{1\}}$	0.03078$^{\{5\}}$	0.0426$^{\{9\}}$	0.03908$^{\{7\}}$	0.04228$^{\{8\}}$	0.03534$^{\{6\}}$	0.04287$^{\{10\}}$
	ASAE		0.02036$^{\{5\}}$	0.01865$^{\{2\}}$	0.01884$^{\{3\}}$	0.02022$^{\{4\}}$	0.01849$^{\{1\}}$	0.02932$^{\{9\}}$	0.02651$^{\{7\}}$	0.02907$^{\{8\}}$	0.02458$^{\{6\}}$	0.02964$^{\{10\}}$
	$\sum$ Ranks		26$^{\{2\}}$	57$^{\{5\}}$	67$^{\{6\}}$	16$^{\{1\}}$	51$^{\{3\}}$	100$^{\{8\}}$	72$^{\{7\}}$	106$^{\{9\}}$	52$^{\{4\}}$	113$^{\{10\}}$
150	BIAS	$\hat{a}$	0.11071$^{\{1\}}$	0.11432$^{\{3\}}$	0.13016$^{\{6\}}$	0.11136$^{\{2\}}$	0.12903$^{\{5\}}$	0.13896$^{\{8\}}$	0.1356$^{\{7\}}$	0.15061$^{\{10\}}$	0.1274$^{\{4\}}$	0.1457$^{\{9\}}$
		$\hat{b}$	0.08149$^{\{1\}}$	0.08453$^{\{2\}}$	0.08551$^{\{3\}}$	0.09062$^{\{5\}}$	0.09207$^{\{6\}}$	0.09628$^{\{8\}}$	0.09481$^{\{7\}}$	0.10032$^{\{9\}}$	0.08969$^{\{4\}}$	0.10061$^{\{10\}}$
		$\hat{c}$	0.06202$^{\{2\}}$	0.06587$^{\{3\}}$	0.07332$^{\{6\}}$	0.06056$^{\{1\}}$	0.07326$^{\{5\}}$	0.08709$^{\{10\}}$	0.08002$^{\{7\}}$	0.08708$^{\{9\}}$	0.07139$^{\{4\}}$	0.08497$^{\{8\}}$
	MSE	$\hat{a}$	0.01929$^{\{2\}}$	0.02093$^{\{3\}}$	0.02709$^{\{6\}}$	0.0187$^{\{1\}}$	0.02613$^{\{5\}}$	0.02998$^{\{8\}}$	0.02832$^{\{7\}}$	0.03551$^{\{10\}}$	0.02483$^{\{4\}}$	0.0336$^{\{9\}}$
		$\hat{b}$	0.00996$^{\{1\}}$	0.0108$^{\{2\}}$	0.01118$^{\{3\}}$	0.0123$^{\{5\}}$	0.01258$^{\{6\}}$	0.01361$^{\{8\}}$	0.01307$^{\{7\}}$	0.01438$^{\{9\}}$	0.01194$^{\{4\}}$	0.01453$^{\{10\}}$
		$\hat{c}$	0.00655$^{\{2\}}$	0.00874$^{\{4\}}$	0.01056$^{\{6\}}$	0.00594$^{\{1\}}$	0.01049$^{\{5\}}$	0.01278$^{\{9\}}$	0.01065$^{\{7\}}$	0.01292$^{\{10\}}$	0.00833$^{\{3\}}$	0.01154$^{\{8\}}$
	MRE	$\hat{a}$	0.11071$^{\{1\}}$	0.11432$^{\{3\}}$	0.13016$^{\{6\}}$	0.11136$^{\{2\}}$	0.12903$^{\{5\}}$	0.13896$^{\{8\}}$	0.1356$^{\{7\}}$	0.15061$^{\{10\}}$	0.1274$^{\{4\}}$	0.1457$^{\{9\}}$
		$\hat{b}$	0.32597$^{\{1\}}$	0.33812$^{\{2\}}$	0.34203$^{\{3\}}$	0.36246$^{\{5\}}$	0.36829$^{\{6\}}$	0.38512$^{\{8\}}$	0.37922$^{\{7\}}$	0.40128$^{\{9\}}$	0.35876$^{\{4\}}$	0.40243$^{\{10\}}$
		$\hat{c}$	0.06891$^{\{2\}}$	0.07319$^{\{3\}}$	0.08146$^{\{6\}}$	0.06729$^{\{1\}}$	0.0814$^{\{5\}}$	0.09676$^{\{10\}}$	0.08891$^{\{7\}}$	0.09675$^{\{9\}}$	0.07933$^{\{4\}}$	0.09441$^{\{8\}}$
	D_{abs}		0.01278$^{\{2\}}$	0.01262$^{\{1\}}$	0.01394$^{\{5\}}$	0.01282$^{\{3\}}$	0.01362$^{\{4\}}$	0.01901$^{\{8\}}$	0.01739$^{\{7\}}$	0.02081$^{\{10\}}$	0.01618$^{\{6\}}$	0.0208$^{\{9\}}$
	$D_{\max}$		0.02118$^{\{2\}}$	0.02104$^{\{1\}}$	0.02324$^{\{5\}}$	0.02126$^{\{3\}}$	0.02281$^{\{4\}}$	0.03054$^{\{8\}}$	0.02808$^{\{7\}}$	0.0332$^{\{10\}}$	0.02626$^{\{6\}}$	0.03303$^{\{9\}}$
	ASAE		0.01374$^{\{4\}}$	0.01312$^{\{2.5\}}$	0.01312$^{\{2.5\}}$	0.01404$^{\{5\}}$	0.01304$^{\{1\}}$	0.02031$^{\{8\}}$	0.01851$^{\{7\}}$	0.02115$^{\{9\}}$	0.01711$^{\{6\}}$	0.02121$^{\{10\}}$
	$\sum$ Ranks		21$^{\{1\}}$	29.5$^{\{2\}}$	57.5$^{\{6\}}$	34$^{\{3\}}$	57$^{\{5\}}$	101$^{\{8\}}$	84$^{\{7\}}$	114$^{\{10\}}$	53$^{\{4\}}$	109$^{\{9\}}$
250	BIAS	$\hat{a}$	0.09058$^{\{1\}}$	0.09185$^{\{2\}}$	0.10628$^{\{6\}}$	0.09857$^{\{3\}}$	0.1002$^{\{4\}}$	0.12137$^{\{8\}}$	0.10928$^{\{7\}}$	0.12454$^{\{10\}}$	0.10357$^{\{5\}}$	0.12394$^{\{9\}}$
		$\hat{b}$	0.07055$^{\{1\}}$	0.07353$^{\{2\}}$	0.07857$^{\{4\}}$	0.08346$^{\{5\}}$	0.07662$^{\{3\}}$	0.08888$^{\{8\}}$	0.08404$^{\{6\}}$	0.0956$^{\{10\}}$	0.08474$^{\{7\}}$	0.09173$^{\{9\}}$
		$\hat{c}$	0.05392$^{\{2\}}$	0.05953$^{\{4\}}$	0.06246$^{\{5\}}$	0.05311$^{\{1\}}$	0.06335$^{\{6\}}$	0.07222$^{\{10\}}$	0.06832$^{\{7\}}$	0.06987$^{\{8\}}$	0.05846$^{\{3\}}$	0.07048$^{\{9\}}$
	MSE	$\hat{a}$	0.01243$^{\{1\}}$	0.01302$^{\{2\}}$	0.01793$^{\{6\}}$	0.01442$^{\{3\}}$	0.01613$^{\{4\}}$	0.02284$^{\{8\}}$	0.01835$^{\{7\}}$	0.0244$^{\{10\}}$	0.01691$^{\{5\}}$	0.02359$^{\{9\}}$
		$\hat{b}$	0.00776$^{\{1\}}$	0.00851$^{\{2\}}$	0.00961$^{\{4\}}$	0.01088$^{\{6\}}$	0.00947$^{\{3\}}$	0.01152$^{\{8\}}$	0.01076$^{\{5\}}$	0.01329$^{\{10\}}$	0.01091$^{\{7\}}$	0.01251$^{\{9\}}$
		$\hat{c}$	0.00479$^{\{1\}}$	0.00615$^{\{4\}}$	0.00755$^{\{5.5\}}$	0.00501$^{\{2\}}$	0.00773$^{\{8\}}$	0.00855$^{\{10\}}$	0.0077$^{\{7\}}$	0.00755$^{\{5.5\}}$	0.00517$^{\{3\}}$	0.00813$^{\{9\}}$
	MRE	$\hat{a}$	0.09058$^{\{1\}}$	0.09185$^{\{2\}}$	0.10628$^{\{6\}}$	0.09857$^{\{3\}}$	0.1002$^{\{4\}}$	0.12137$^{\{8\}}$	0.10928$^{\{7\}}$	0.12454$^{\{10\}}$	0.10357$^{\{5\}}$	0.12394$^{\{9\}}$
		$\hat{b}$	0.28219$^{\{1\}}$	0.29412$^{\{2\}}$	0.31428$^{\{4\}}$	0.33386$^{\{5\}}$	0.30647$^{\{3\}}$	0.35553$^{\{8\}}$	0.33616$^{\{6\}}$	0.38239$^{\{10\}}$	0.33895$^{\{7\}}$	0.36692$^{\{9\}}$
		$\hat{c}$	0.05991$^{\{2\}}$	0.06614$^{\{4\}}$	0.0694$^{\{5\}}$	0.05901$^{\{1\}}$	0.07038$^{\{6\}}$	0.08024$^{\{10\}}$	0.07591$^{\{7\}}$	0.07763$^{\{8\}}$	0.06495$^{\{3\}}$	0.07831$^{\{9\}}$
	D_{abs}		0.00988$^{\{1\}}$	0.01038$^{\{3\}}$	0.01056$^{\{5\}}$	0.01009$^{\{2\}}$	0.01046$^{\{4\}}$	0.01483$^{\{8\}}$	0.01335$^{\{7\}}$	0.01657$^{\{9\}}$	0.01214$^{\{6\}}$	0.01671$^{\{10\}}$
	$D_{\max}$		0.01661$^{\{1\}}$	0.01736$^{\{3\}}$	0.01796$^{\{5\}}$	0.017$^{\{2\}}$	0.0176$^{\{4\}}$	0.02443$^{\{8\}}$	0.02198$^{\{7\}}$	0.02678$^{\{9\}}$	0.02015$^{\{6\}}$	0.02692$^{\{10\}}$
	ASAE		0.01061$^{\{4\}}$	0.01019$^{\{3\}}$	0.00985$^{\{1\}}$	0.01093$^{\{5\}}$	0.00997$^{\{2\}}$	0.01539$^{\{8\}}$	0.01409$^{\{7\}}$	0.01659$^{\{9\}}$	0.0132$^{\{6\}}$	0.01672$^{\{10\}}$
	$\sum$ Ranks		17$^{\{1\}}$	33$^{\{2\}}$	56.5$^{\{5\}}$	38$^{\{3\}}$	51$^{\{4\}}$	102$^{\{8\}}$	80$^{\{7\}}$	108.5$^{\{9\}}$	63$^{\{6\}}$	111$^{\{10\}}$
400	BIAS	$\hat{a}$	0.07044$^{\{1\}}$	0.07738$^{\{2\}}$	0.08431$^{\{4\}}$	0.0777$^{\{3\}}$	0.08804$^{\{5\}}$	0.10094$^{\{8\}}$	0.09681$^{\{7\}}$	0.10586$^{\{10\}}$	0.09054$^{\{6\}}$	0.10514$^{\{9\}}$
		$\hat{b}$	0.0582$^{\{1\}}$	0.06129$^{\{2\}}$	0.06343$^{\{3\}}$	0.06823$^{\{5\}}$	0.06778$^{\{4\}}$	0.0789$^{\{8\}}$	0.07846$^{\{7\}}$	0.08384$^{\{9\}}$	0.07229$^{\{6\}}$	0.08462$^{\{10\}}$
		$\hat{c}$	0.05239$^{\{2\}}$	0.05485$^{\{3\}}$	0.06068$^{\{6\}}$	0.04835$^{\{1\}}$	0.0617$^{\{10\}}$	0.06111$^{\{8\}}$	0.05985$^{\{5\}}$	0.06143$^{\{9\}}$	0.05644$^{\{4\}}$	0.06073$^{\{7\}}$
	MSE	$\hat{a}$	0.00768$^{\{1\}}$	0.00922$^{\{3\}}$	0.01098$^{\{4\}}$	0.00918$^{\{2\}}$	0.01179$^{\{5\}}$	0.01583$^{\{8\}}$	0.01446$^{\{7\}}$	0.0177$^{\{10\}}$	0.01246$^{\{6\}}$	0.01678$^{\{9\}}$
		$\hat{b}$	0.0056$^{\{1\}}$	0.00622$^{\{2\}}$	0.00685$^{\{3\}}$	0.00765$^{\{4\}}$	0.0077$^{\{5\}}$	0.00992$^{\{8\}}$	0.00979$^{\{7\}}$	0.01095$^{\{9\}}$	0.00828$^{\{6\}}$	0.01098$^{\{10\}}$
		$\hat{c}$	0.00433$^{\{2\}}$	0.00502$^{\{4\}}$	0.00735$^{\{9\}}$	0.00357$^{\{1\}}$	0.00744$^{\{10\}}$	0.00621$^{\{8\}}$	0.00561$^{\{5\}}$	0.00583$^{\{7\}}$	0.00497$^{\{3\}}$	0.00578$^{\{6\}}$
	MRE	$\hat{a}$	0.07044$^{\{1\}}$	0.07738$^{\{2\}}$	0.08431$^{\{4\}}$	0.0777$^{\{3\}}$	0.08804$^{\{5\}}$	0.10094$^{\{8\}}$	0.09681$^{\{7\}}$	0.10586$^{\{10\}}$	0.09054$^{\{6\}}$	0.10514$^{\{9\}}$
		$\hat{b}$	0.23279$^{\{1\}}$	0.24515$^{\{2\}}$	0.25373$^{\{3\}}$	0.27293$^{\{5\}}$	0.27111$^{\{4\}}$	0.31561$^{\{8\}}$	0.31384$^{\{7\}}$	0.33535$^{\{9\}}$	0.28917$^{\{6\}}$	0.33849$^{\{10\}}$
		$\hat{c}$	0.05821$^{\{2\}}$	0.06094$^{\{3\}}$	0.06743$^{\{6\}}$	0.05372$^{\{1\}}$	0.06855$^{\{10\}}$	0.0679$^{\{8\}}$	0.0665$^{\{5\}}$	0.06826$^{\{9\}}$	0.06271$^{\{4\}}$	0.06748$^{\{7\}}$
	D_{abs}		0.00812$^{\{2\}}$	0.00832$^{\{3\}}$	0.00839$^{\{4\}}$	0.00809$^{\{1\}}$	0.00847$^{\{5\}}$	0.01202$^{\{8\}}$	0.01125$^{\{7\}}$	0.01364$^{\{10\}}$	0.01019$^{\{6\}}$	0.0133$^{\{9\}}$
	$D_{\max}$		0.01356$^{\{1\}}$	0.01397$^{\{3\}}$	0.0143$^{\{4\}}$	0.01369$^{\{2\}}$	0.01444$^{\{5\}}$	0.01992$^{\{8\}}$	0.01861$^{\{7\}}$	0.02224$^{\{10\}}$	0.01707$^{\{6\}}$	0.02188$^{\{9\}}$
	ASAE		0.00838$^{\{4\}}$	0.00789$^{\{3\}}$	0.00782$^{\{2\}}$	0.00844$^{\{5\}}$	0.00771$^{\{1\}}$	0.01211$^{\{8\}}$	0.01112$^{\{7\}}$	0.01346$^{\{9.5\}}$	0.01038$^{\{6\}}$	0.01346$^{\{9.5\}}$
	$\sum$ Ranks		19$^{\{1\}}$	32$^{\{2\}}$	52$^{\{4\}}$	33$^{\{3\}}$	69$^{\{6\}}$	96$^{\{8\}}$	78$^{\{7\}}$	111.5$^{\{10\}}$	65$^{\{5\}}$	104.5$^{\{9\}}$

Table 11: Numerical values for MSE of SRS divided by MSE of RSS for all estimators.

$s^\bullet$	Par.	ML	AD	CRM	MXPS	LS	SPAD	SPALoD	MSSD	MSSLD	MSLND
						$a = 1.5, b = 0.75, c = 2.0$					
30	$\hat{a}$	1.42505	1.47447	1.18021	1.52760	1.34015	1.22012	1.41530	1.28208	1.33849	1.26090
	$\hat{b}$	1.08526	1.16284	1.09050	1.09499	1.14621	1.00707	1.14564	1.11890	1.07303	1.14316
	$\hat{c}$	1.00191	1.30453	1.29713	1.48368	1.34626	1.15493	1.26068	1.11995	1.14902	1.32732
75	$\hat{a}$	1.57859	1.36756	1.48582	1.51734	1.59603	1.27344	1.22924	1.14594	1.40799	1.38829
	$\hat{b}$	1.28673	1.10843	1.13560	1.04241	1.22528	1.08648	1.05712	1.01145	1.10793	1.08905
	$\hat{c}$	1.14628	1.10473	1.35395	1.17246	1.40182	1.03882	1.20321	1.09754	1.20123	1.31599
150	$\hat{a}$	1.59408	1.36841	1.25416	1.28526	1.46929	1.25817	1.21938	1.24901	1.27702	1.22190
	$\hat{b}$	1.31678	1.17887	1.07644	1.10364	1.09493	1.05390	1.03087	1.09098	1.06087	1.03459
	$\hat{c}$	1.37442	1.32460	1.06797	1.26639	1.15706	1.23465	1.01317	1.22718	1.09209	1.07047
250	$\hat{a}$	1.63851	1.57433	1.32733	1.24499	1.32629	1.08890	1.21473	1.17910	1.32213	1.31581
	$\hat{b}$	1.44079	1.22222	1.12508	1.04181	1.08074	1.07116	1.09337	1.12118	1.16047	1.07650
	$\hat{c}$	1.30753	1.50353	1.11105	1.16224	1.07847	1.26736	1.20416	1.01389	1.33814	1.26568
400	$\hat{a}$	1.45119	1.44067	1.36607	1.13851	1.37886	1.14331	1.25711	1.28723	1.06549	1.19342
	$\hat{b}$	1.42175	1.24619	1.15914	1.16620	1.27990	1.13723	1.20204	1.09876	1.06402	1.12245
	$\hat{c}$	1.15548	1.20500	1.34555	1.03136	1.11917	1.00082	1.28816	1.19919	1.20138	1.23264
						$a = 0.25, b = 2.5, c = 1.5$					
30	$\hat{a}$	1.06888	1.13568	1.00991	1.07903	1.05677	1.05207	1.07598	1.04631	1.01157	1.07024
	$\hat{b}$	1.41202	1.25077	1.31128	1.32977	1.36555	1.22740	1.38772	1.13791	1.30246	1.14278
	$\hat{c}$	1.26880	1.24252	1.22822	1.32110	1.36037	1.19834	1.26830	1.11925	1.25248	1.11929
75	$\hat{a}$	1.05948	1.08911	1.08289	1.05790	1.00874	1.08091	1.04730	1.00595	1.05944	1.04609
	$\hat{b}$	1.09620	1.34149	1.28703	1.31397	1.15327	1.15224	1.24736	1.09399	1.22685	1.21448
	$\hat{c}$	1.19874	1.26470	1.28640	1.24132	1.09131	1.11521	1.12431	1.21082	1.20628	1.17334
150	$\hat{a}$	1.17050	1.11765	1.07441	1.04639	1.02160	1.03324	1.06565	1.04609	1.08583	1.03987
	$\hat{b}$	1.14948	1.06847	1.09212	1.13522	1.06736	1.17683	1.15346	1.19684	1.15449	1.17012
	$\hat{c}$	1.23899	1.16611	1.06202	1.16093	1.08971	1.14775	1.09733	1.09716	1.16116	1.16100
250	$\hat{a}$	1.16566	1.13077	1.14747	1.08739	1.06063	1.00588	1.05373	1.00968	1.10595	1.06198
	$\hat{b}$	1.03385	1.07495	1.04492	1.13905	1.09072	1.14944	1.15495	1.06435	1.12230	1.11567
	$\hat{c}$	1.16925	1.24570	1.13277	1.35964	1.15256	1.17705	1.22294	1.08559	1.16541	1.08696
400	$\hat{a}$	1.21799	1.10019	1.08735	1.10374	1.16013	1.09752	1.05476	1.04107	1.04782	1.07965
	$\hat{b}$	1.11820	1.03634	1.06291	1.08591	1.06162	1.22044	1.15679	1.06826	1.11329	1.05764
	$\hat{c}$	1.28157	1.16915	1.11168	1.20548	1.23019	1.16328	1.10795	1.11865	1.16270	1.07891
						$a = 0.9, b = 2.0, c = 2.5$					
30	$\hat{a}$	1.27887	1.10079	1.18059	1.11554	1.14395	1.03337	1.11764	1.21566	1.02920	1.16809
	$\hat{b}$	1.24127	1.22545	1.12112	1.10930	1.09700	1.05357	1.09152	1.15120	1.10023	1.08043
	$\hat{c}$	1.09424	1.12062	1.23848	1.03321	1.19086	1.04728	1.14756	1.08980	1.13409	1.21726
75	$\hat{a}$	1.42013	1.37379	1.34989	1.41672	1.29639	1.14346	1.09944	1.14379	1.07338	1.15272
	$\hat{b}$	1.35595	1.17675	1.22318	1.30261	1.16770	1.09728	1.09398	1.06396	1.08576	1.05088
	$\hat{c}$	1.24627	1.14384	1.27069	1.21251	1.22875	1.03636	1.08743	1.10442	1.12125	1.03903
150	$\hat{a}$	1.55459	1.40849	1.35196	1.26979	1.15274	1.24736	1.24991	1.12097	1.31789	1.08985
	$\hat{b}$	1.26875	1.16309	1.08886	1.10687	1.07967	1.16473	1.09136	1.13832	1.11709	1.07407
	$\hat{c}$	1.07483	1.14685	1.22767	1.14455	1.04137	1.11428	1.07241	1.07896	1.17703	1.03818
250	$\hat{a}$	1.51890	1.26412	1.14218	1.15293	1.16098	1.25512	1.07295	1.18325	1.18214	1.13322
	$\hat{b}$	1.27820	1.14337	1.09111	1.12887	1.06795	1.13507	1.07258	1.08984	1.11780	1.01260
	$\hat{c}$	1.33396	1.15534	1.07527	1.09623	1.04189	1.30933	1.05408	1.03133	1.14328	1.02365
400	$\hat{a}$	1.54943	1.30649	1.38220	1.12558	1.26685	1.12172	1.08178	1.13904	1.17455	1.21058
	$\hat{b}$	1.29691	1.25902	1.15500	1.08621	1.24119	1.02747	1.06438	1.06726	1.14315	1.07621
	$\hat{c}$	1.37334	1.18119	1.13075	1.03452	1.08364	1.11535	1.07105	1.14251	1.08168	1.18020
						$a = 2.0, b = 1.0, c = 0.5$					
30	$\hat{a}$	1.03951	1.29323	1.09765	1.28023	1.02536	1.08055	1.07839	1.12832	1.21561	1.08802
	$\hat{b}$	1.00609	1.29689	1.17784	1.21965	1.14014	1.13776	1.05534	1.16376	1.35169	1.11886
	$\hat{c}$	1.11224	1.09003	1.04589	1.24223	1.10230	1.06994	1.01443	1.05629	1.08181	1.08791
75	$\hat{a}$	1.13277	1.25765	1.21288	1.18681	1.24871	1.08111	1.22048	1.18834	1.25691	1.22943
	$\hat{b}$	1.00637	1.28316	1.16129	1.14197	1.22918	1.04537	1.08911	1.11127	1.18995	1.11695
	$\hat{c}$	1.23705	1.03778	1.06826	1.11144	1.19076	1.06973	1.12978	1.06553	1.10717	1.06488
150	$\hat{a}$	1.07764	1.25483	1.11784	1.13394	1.32744	1.21943	1.10904	1.19244	1.13498	1.18674
	$\hat{b}$	1.03381	1.18259	1.18157	1.14576	1.19867	1.07146	1.10367	1.13226	1.18185	1.05028
	$\hat{c}$	1.17626	1.15700	1.11704	1.11123	1.22995	1.08558	1.11560	1.10779	1.05138	1.04536
250	$\hat{a}$	1.10256	1.09366	1.36015	1.36335	1.23401	1.14683	1.09407	1.29402	1.05972	1.10093
	$\hat{b}$	1.00144	1.14287	1.25257	1.16552	1.15399	1.14524	1.13055	1.22112	1.04282	1.13954
	$\hat{c}$	1.16381	1.19233	1.10853	1.15788	1.14668	1.07313	1.04135	1.07806	1.09655	1.03475
400	$\hat{a}$	1.23895	1.38868	1.14617	1.20404	1.32223	1.08524	1.22563	1.17452	1.21578	1.09681
	$\hat{b}$	1.10914	1.17864	1.08224	1.08147	1.11866	1.07575	1.09447	1.11327	1.15277	1.07235
	$\hat{c}$	1.18970	1.20231	1.11311	1.19722	1.16318	1.12145	1.10612	1.02483	1.16652	1.03363
						$a = 1.0, b = 0.25, c = 0.9$					
30	$\hat{a}$	1.65480	1.74583	1.49713	1.69099	1.80379	1.38050	1.30938	1.28643	1.29975	1.26386
	$\hat{b}$	1.04440	1.08691	1.08526	1.06494	1.07091	1.08215	1.02752	1.04732	1.00815	1.05176
	$\hat{c}$	1.66252	1.80010	1.75276	2.03792	1.93008	1.45770	1.42468	1.34989	1.28218	1.47426
75	$\hat{a}$	1.73706	1.43048	1.46591	1.50343	1.51274	1.34977	1.25806	1.34235	1.43232	1.32089
	$\hat{b}$	1.18084	1.04276	1.08803	1.01677	1.19841	1.11693	1.16800	1.05308	1.05493	1.03450
	$\hat{c}$	1.82880	1.57323	1.25721	1.87351	1.67481	1.23066	1.30695	1.30015	1.53871	1.38784
150	$\hat{a}$	1.73406	1.59006	1.44297	1.38128	1.38308	1.32422	1.26871	1.27964	1.41120	1.32440
	$\hat{b}$	1.25201	1.23148	1.27728	1.06504	1.20270	1.09111	1.11247	1.08484	1.13819	1.05850
	$\hat{c}$	1.81832	1.35355	1.40625	1.56566	1.32412	1.37715	1.25164	1.27709	1.32413	1.35875
250	$\hat{a}$	1.61786	1.42243	1.39822	1.27601	1.43335	1.20403	1.17003	1.33852	1.31638	1.36753
	$\hat{b}$	1.29381	1.22562	1.21020	1.03585	1.20063	1.08681	1.13290	1.01505	1.08249	1.01918
	$\hat{c}$	1.57411	1.34472	1.55762	1.38523	1.48771	1.46433	1.30390	1.55629	1.58414	1.47970
400	$\hat{a}$	1.64323	1.51302	1.50455	1.33333	1.37744	1.17562	1.11480	1.19040	1.18700	1.36949
	$\hat{b}$	1.33393	1.40354	1.32993	1.20392	1.21429	1.04940	1.03473	1.08128	1.13889	1.10656
	$\hat{c}$	1.19400	1.30677	1.16735	1.56022	1.14382	1.48309	1.26203	1.31561	1.27767	1.52249

Table 12: Partial and overall ranks for all estimation methods using SRS.

Parameter	$s^{\bullet}$	ML	AD	CRM	MXPS	LS	SPAD	SPALoD	MSSD	MSSLD	MSLND
$a = 1.5, b = 0.75, c = 2.0$	30	3.0	7.0	10.0	1.0	6.0	5.0	4.0	8.0	2.0	9.0
	75	4.0	2.0	9.0	1.0	6.0	8.0	5.0	7.0	3.0	10.0
	150	3.0	2.0	6.5	1.0	5.0	9.0	6.5	10.0	4.0	8.0
	250	2.0	5.0	3.0	1.0	6.0	9.0	7.0	10.0	4.0	8.0
	400	1.0	3.0	6.0	2.0	5.0	8.0	7.0	9.0	4.0	10.0
$a = 0.25, b = 2.5, c = 1.5$	30	3.0	4.0	2.0	1.0	7.0	6.0	10.0	8.0	5.0	9.0
	75	3.0	8.0	7.0	2.0	1.0	4.0	6.0	9.0	5.0	10.0
	150	3.5	8.0	5.0	1.0	3.5	6.0	7.0	10.0	2.0	9.0
	250	2.0	8.0	5.0	1.0	4.0	6.0	7.0	9.0	3.0	10.0
	400	1.5	3.0	6.0	1.5	8.0	7.0	4.5	10.0	4.5	9.0
$a = 0.9, b = 2.0, c = 2.5$	30	9.0	5.0	10.0	1.0	2.0	4.0	8.0	7.0	3.0	6.0
	75	4.0	3.0	9.5	1.0	6.0	9.5	5.0	7.5	2.0	7.5
	150	2.0	3.0	6.0	1.0	5.0	8.0	7.0	10.0	4.0	9.0
	250	1.0	3.0	4.0	2.0	5.0	8.0	7.0	10.0	6.0	9.0
	400	1.0	3.0	5.5	2.0	4.0	8.0	7.0	10.0	5.5	9.0
$a = 2.0, b = 1.0, c = 0.5$	30	1.0	7.0	5.0	4.0	6.0	3.0	2.0	10.0	8.0	9.0
	75	2.0	6.0	8.0	1.0	7.0	3.0	5.0	9.0	4.0	10.0
	150	2.0	4.0	7.0	1.0	8.0	5.5	5.5	9.0	3.0	10.0
	250	2.0	4.0	7.0	1.0	5.0	8.0	6.0	10.0	3.0	9.0
	400	3.0	4.0	5.0	1.0	8.0	7.0	6.0	9.0	2.0	10.0
$a = 1.0, b = 0.25, c = 0.9$	30	2.0	5.0	7.0	1.0	6.0	10.0	4.0	9.0	3.0	8.0
	75	2.0	4.0	5.0	1.0	6.0	8.0	7.0	9.0	3.0	10.0
	150	3.0	2.0	6.0	1.0	5.0	8.0	7.0	10.0	4.0	9.0
	250	2.0	3.0	6.0	1.0	4.0	8.0	7.0	10.0	5.0	9.0
	400	1.0	3.0	5.0	2.0	6.0	8.0	7.0	9.0	4.0	10.0
$\sum$ Ranks		63.0	109.0	155.5	33.5	134.5	174.0	154.5	228.5	96.0	226.5
Overall Rank		2	4	7	1	5	8	6	10	3	9

Table 13: Partial and overall ranks for all estimation methods using RSS.

Parameter	$s^{\bullet}$	ML	AD	CRM	MXPS	LS	SPAD	SPALoD	MSSD	MSSLD	MSLND
$a = 1.5, b = 0.75, c = 2.0$	30	3.0	6.0	9.0	1.0	5.0	10.0	2.0	8.0	4.0	7.0
	75	1.0	5.0	6.0	2.0	3.0	9.0	7.0	10.0	4.0	8.0
	150	1.0	3.0	6.0	2.0	4.0	8.5	7.0	8.5	5.0	10.0
	250	1.0	2.0	6.0	3.0	5.0	9.0	7.0	10.0	4.0	8.0
	400	1.0	2.0	5.0	3.0	4.0	8.0	7.0	10.0	6.0	9.0
$a = 1.5, b = 0.75, c = 2.0$	30	2.0	4.0	5.0	1.0	3.0	8.0	6.0	10.0	7.0	9.0
	75	5.0	2.0	6.0	1.0	4.0	7.0	8.0	10.0	3.0	9.0
	150	2.0	4.0	7.5	1.0	6.0	5.0	7.5	10.0	3.0	9.0
	250	2.0	3.5	5.0	1.0	6.0	8.0	7.0	10.0	3.5	9.0
	400	1.0	3.0	8.0	2.0	6.0	7.0	5.0	10.0	4.0	9.0
$a = 1.5, b = 0.75, c = 2.0$	30	3.0	2.0	9.0	1.0	4.0	10.0	8.0	7.0	5.0	6.0
	75	2.0	3.0	4.5	1.0	4.5	8.0	7.0	9.0	6.0	10.0
	150	2.0	3.0	5.0	1.0	6.0	8.0	7.0	9.0	4.0	10.0
	250	1.0	3.0	6.0	2.0	5.0	8.0	7.0	10.0	4.0	9.0
	400	1.0	2.0	5.0	3.0	4.0	8.0	7.0	10.0	6.0	9.0
$a = 1.5, b = 0.75, c = 2.0$	30	3.0	5.0	8.0	1.0	7.0	4.0	6.0	9.0	2.0	10.0
	75	3.0	7.0	8.0	1.0	5.0	6.0	4.0	9.0	2.0	10.0
	150	2.0	4.0	8.0	1.0	5.0	6.0	7.0	9.0	3.0	10.0
	250	3.0	2.0	5.5	1.0	5.5	7.0	8.0	9.5	4.0	9.5
	400	3.0	2.0	7.5	1.0	5.0	7.5	6.0	9.0	4.0	10.0
$a = 1.5, b = 0.75, c = 2.0$	30	2.0	3.0	6.5	1.0	4.0	10.0	5.0	9.0	6.5	8.0
	75	2.0	5.0	6.0	1.0	3.0	8.0	7.0	9.0	4.0	10.0
	150	1.0	2.0	6.0	3.0	5.0	8.0	7.0	10.0	4.0	9.0
	250	1.0	2.0	5.0	3.0	4.0	8.0	7.0	9.0	6.0	10.0
	400	1.0	2.0	4.0	3.0	6.0	8.0	7.0	10.0	5.0	9.0
$\sum$ Ranks		49.0	81.5	157.5	41.0	119.0	194.0	163.5	234.0	109.0	226.5
Overall Rank		2	3	6	1	5	8	7	10	4	9
